# Transcriptome analysis during ripening of table grape berry cv. Thompson Seedless

**DOI:** 10.1371/journal.pone.0190087

**Published:** 2018-01-10

**Authors:** Iván Balic, Paula Vizoso, Ricardo Nilo-Poyanco, Dayan Sanhueza, Patricio Olmedo, Pablo Sepúlveda, Cesar Arriagada, Bruno G. Defilippi, Claudio Meneses, Reinaldo Campos-Vargas

**Affiliations:** 1 Universidad Andrés Bello, Facultad Ciencias Biológicas, Centro de Biotecnología Vegetal, Santiago, Chile; 2 Universidad de Los Lagos, Departamento de Acuicultura y Recursos Agroalimentarios, Osorno, Chile; 3 Center of Plant Propagation and Conservation (CEPROVEG), Faculty of Sciences, Universidad Mayor, Santiago, Chile; 4 FONDAP Center for Genome Regulation, Santiago, Chile; 5 Laboratorio Biorremediación, Departamento de Ciencias Forestales, Facultad de Ciencias Agropecuarias y Forestales, Universidad de La Frontera, Temuco, Chile; 6 Instituto de Investigaciones Agropecuarias, INIA La Platina, Santiago, Chile; Mediterranean Agronomic Institute of Chania, GREECE

## Abstract

Ripening is one of the key processes associated with the development of major organoleptic characteristics of the fruit. This process has been extensively characterized in climacteric fruit, in contrast with non-climacteric fruit such as grape, where the process is less understood. With the aim of studying changes in gene expression during ripening of non-climacteric fruit, an Illumina based RNA-Seq transcriptome analysis was performed on four developmental stages, between veraison and harvest, on table grapes berries cv Thompson Seedless. Functional analysis showed a transcriptional increase in genes related with degradation processes of chlorophyll, lipids, macromolecules recycling and nucleosomes organization; accompanied by a decrease in genes related with chloroplasts integrity and amino acid synthesis pathways. It was possible to identify several processes described during leaf senescence, particularly close to harvest. Before this point, the results suggest a high transcriptional activity associated with the regulation of gene expression, cytoskeletal organization and cell wall metabolism, which can be related to growth of berries and firmness loss characteristic to this stage of development. This high metabolic activity could be associated with an increase in the transcription of genes related with glycolysis and respiration, unexpected for a non-climacteric fruit ripening.

## Introduction

Fruit ripening is a complex, finely coordinated process, that involves a series of important physiological changes that affect the fruit palatability [1,2]. Changes occurring during fruit ripening include epidermis discoloration, increase in soluble sugars concentration and acidity reduction, pulp softening and aroma intensification [3]. In climacteric fruits these changes are associated with a characteristic increasing in respiration, accompanied by an increase in ethylene production [4]. Given the economic relevance of many climacteric fruits, where these changes are associated with a characteristic increasing in respiration and ethylene production, several studies have been performed in order to characterize the metabolic and transcriptional changes associated to changes in the perception of fruit sensory attributes [5–7].

Grapes belong to the group of non-climacteric fruit, commonly associated with a lack of a marked increase in respiration or ethylene generation during ripening, despite that recent findings pointed to a differential ethylene response during grape development, and increased expression at veraison of ethylene biosynthesis related genes in Thompson Seedless [8–10]. Grape berry development shows a double-sigmoidal growth curve defining three growth phases, two fast-growing (phase I and phase III), separated by a slower lag-growth stage (phase-II) known as veraison [11,12]. During this phase, the accumulation of organic acids reaches its maximum level and chlorophyll is degraded giving rise to the beginning of color change characteristic of each cultivar [13,14]. This process marks the beginning of ripening characterized among other by berry softening and cell expansion due to the active accumulation of water in the vacuole [12].

Recently there has been an increase in the amount of transcriptome studies which have helped to unravel some of the main molecular mechanisms involved in the ripening of grape berries [15–18]. Most of these studies have been conducted in wine grape cultivars and focused to answer questions related to biological processes of interest to the winery industry. However, quality attributes of table grape berries differ from wine grapes in several aspects, some of them in an opposite way. One example is the higher firmness in table grape berries which contribute to desirable crunchy texture, while in wine grapes is correlated with lower phenolic compounds extraction [19,20]. Therefore, the application of the knowledge derived from wine berries into table berries is not straightforward. The present study aims to increase the knowledge gap among table and wine berries ripening by means of a transcriptome analysis using the white-green table grapes cultivar Thompson Seedless.

## Material and methods

### Plant material and maturity parameters

Grape bunches of table grapes (*Vitis vinifera*) cultivar Thompson Seedless samples were obtained from a commercial vineyard located in Llay-Llay (Valparaiso Region, Chile) during the 2012 season. The grape bunches were obtained and evaluated weekly from veraison to harvest. The latter was determined based on total soluble solids (18–19% w/w sucrose), according to commercial standard for Thompson Seedless cultivar. Each sampling was performed collecting five grape bunches from different individuals, considering a total of six evaluated points (Table 1). Firmness was determined in 25 grape berries, with five berries from five bunches picked from five different plants, using a Texture Analyser TA-XT plus equipped with the Volodkevich Bite Jaw probe (Stable Micro Systems Ltd., Surrey, UK) by penetration test of whole grape berry (including skin) Complete force-distance curve was obtained at 1 mm s^-1^ of penetration to 15 mm and firmness was calculated by area under the curve (N ● mm) [21,22].

**Table 1:** Maturity parameters of Thompson Seedless table grape cultivar.

TA^a^	DPA^b^	Firmness (N mm)	Diameter (mm)	Soluble Solids (w/w sucrose)	Acidity (g L^-1^)
A1	43	240 ± 5 a*	14.3 ± 0.2 *c*	5.9 ± 0.1 *e*	1.7 ± 0 *a*
A2	52	199 ± 10 *b*	14.3 ± 0.3 *c*	9 ± 0.3 *d*	1.4 ± 0 *b*
-	58	117 ± 4 *c*	16.4 ± 0.3 *b*	12.1 ± 0.2 *c*	1.3 ± 0 *b*
A3	65	99 ± 3 *cd*	18.5 ± 0.3 *a*	13.6 ± 0.2 *b*	0.8 ± 0 *c*
-	72	81 ± 2 *d*	19.0 ± 0.2 *a*	16.4 ± 0.2 *a*	0.6 ± 0 *cd*
A4	78	80 ± 2 *d*	19.0 ± 0.2 *a*	17.2 ± 0.2 *a*	0.5 ± 0 *d*

^a^ Selected point for transcriptomic analysis;

^b^ Days Post Anthesis;

* Letter represent the results of Tukey test p Value < 0.05.

The size of the 25 grape berries was determined according to equatorial diameter measurement using a caliper expressing the value in millimeters. The soluble solids content of the selected berries was determined using a temperature-compensated digital refractometer (HI 96811, Hanna Instruments Inc., Woonsocket USA) expressing the results in % w/w of sucrose per 100 g solution. The acidity was determined by titration of three-pooled juice of 10 berries per bunch previously selected for each sampling point. The titration was performed with 0.1 N NaOH (pH 8.2) and reported as g L^-1^ of tartaric acid.

### Total RNA isolation

Four representative ripening points were selected for transcriptomic analysis (TA) from the total of six sampling points in a temporarily equidistant manner identified as A1 (veraison), A2, A3 and A4 (harvest) (Table 1). Grape berries from five bunches belonging from different plants, were frozen in liquid nitrogen and stored at -80°C immediately after harvest. Frozen berries were grinded until fine powder using liquid nitrogen in a mortar. Total RNA was extracted from entire grape berry according to Gudenschwager et al. [23], and concentration was determined using spectrophotometric method (Epoch, Biotek, VT, USA). For each sampling point it were performed five total RNA extractions. Each RNA extraction was composed of 10 selected berries of five bunches from five plants. The quality of each RNA extraction was confirmed by electrophoresis on 1.2% agarose gel and using capillary electrophoresis Fragment Analyzer Automated CE System (Advanced Analytical Technologies, Inc., IA, USA). The amount of total RNA was determined by fluorometer using Qubit RNA BR assay kit (Invitrogen, CA, USA) according to manufacturer indication. Two RNA pools were constructed for each sampling point by taking 5 μg of RNA per each extraction. The quality and concentration of each RNA pool was confirmed again by capillary electrophoresis and Qubit kit before the library construction.

### RNA-Seq library construction and sequencing

One microgram of total RNA of each sampled pool was used to isolate poly(A) mRNA and to prepare a non-directional Illumina RNA-Seq library with a TruSeq RNA sample preparation according to the manufacturer instructions (Illumina, Inc., San Diego, CA, USA). Library quality control was performed with a Fragment Analyzer Automated CE System (Advanced Analytical Technologies, Inc., IA, USA) using the protocol indicated by the manufacturer. Paired-end libraries were sequenced using HiSeq 2000 platform (Illumina, Inc., San Diego, CA, USA). A total of 100 nucleotides paired-ends reads were generated. Two technical replicates of each A1, A2, A3 and A4 samples were sequenced.

### Sequences processing

Reads sequences were processed according to Q20 quality criteria, trimming reads sequence with Q ≤ 20 and length ≤ 15 nucleotides. Paired-end reads that overlapped in at least 10 nucleotides were selected. Processed reads were mapped against *Vitis vinifera* cv Thompson Seedless reference genome [24] using Tophat 2.0.10 [25]. Counting of mapped reads was performed using the HTSeq package (http://www.huber.embl.de/users/anders/HTSeq/doc/-overview.html) [26].

### Expression and functional analysis

The expression of each transcript was determined by calculating Fragments Per Million per Kilobase (FPKM) of exon mapped reads [27]. FPKM cut-off value above 2, which corresponds to at least 20 reads matched, was applied in order to consider a gene as expressed and be further analyzed. Differential expression analysis was performed at all evaluated instances considering A1 as reference condition using the Edge R package [26,28]. The genes that showed a significant differential expression value in at least one comparison (A2 vs A1; A3 vs A1 or A4 vs A1) were selected according to Baggerly test results (FDR < 0.05) [29]. A false discovery rate threshold (FDR < 0.05) was used in order to determine significant differences in gene expression. Clustering analysis was performed according to FPKM values of each of the genes using *K*-means method considering the Euclidean distance between them and the figure of merits [30,31]. Gene Ontology (GO) terms enrichment analysis of each cluster was performed using AgriGO [32] and complemented with REVIGO tool in order to remove redundant terms based on semantic similarities [33]. For the identification of the most significant GO terms in each cluster, the “Tree Map” visualization of REVIGO was used, considering the term with the most significant p-Value within each sub-set of non-redundant related terms [33]. PlantCyc of Plant Metabolic Network database (http://pmn.plantcyc.org) was used for metabolic pathway analysis [34]. Metabolic Domains enrichment analysis seeks to find if genes associated to a given metabolic domain are overrepresented in a cluster using a hypergeometric test. Pathway enrichment analysis is also based on a hypergeometric test, being constrained to genes associated to pathways.

### Validation by qRT-PCR

qRT-PCR was carried out using the Fast EvaGreen qRT-PCR MasterMix (Applied Biotium, Hayward, CA, USA) and the Mx3000P Real-Time PCR System (Stratagene, La Jolla, USA). Reactions were performed in triplicate containing 50 ng of cDNA, 500 nM of primers, 2X concentration of Fast EvaGreen qRT-PCR MasterMix and nuclease free water with a final volume of 20 μL per reaction. The Ct values for all genes were normalized to the Ct value of gene GSVIVG01000037001 (mevalonate kinase) in each experimental condition, obtaining the best results when compared other evaluated references genes (transcriptional elongation factor II, GSVIVG01025147001; ubiquitin family protein, GSVIVG01008590001). The results obtained for the target genes were normalized using the 2^-ΔΔCT^ method with an efficiency correction [35].

## Results

### Phenotypic characterization

**Fig 1:**
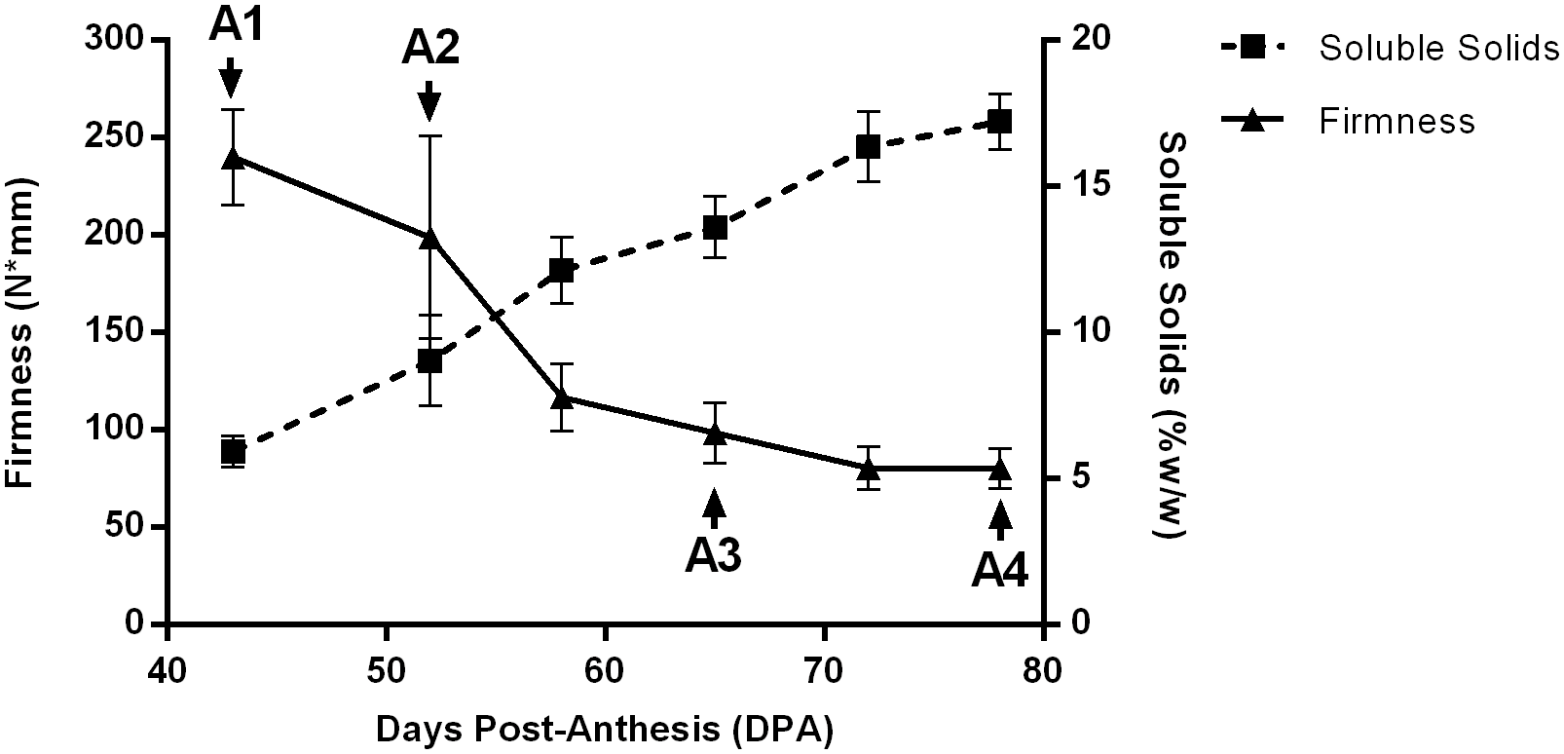
Changes in the fruit firmness (tringles) and soluble solids (squares) of T. Seedless varieties during ripening. A1 (veraison), A2, A3 and A4 (harvest) were selected at different Days Post-Anthesis (DPA) for transcriptomic analysis. Bars of each point show the standard error of the mean.

The highest firmness values were found at 43 DPA (A1) followed by a gradual and significant decrease until 72 DPA, after which values remained practically constant. Soluble solids content showed a linear significant increase from 5.9% at A1 to 17.2% at A4 (Fig 1). The grape berries diameter experienced a significant increase of 33% from A1 (14.3 mm) to A4 (19 mm), while acidity showed a gradual decrease from A1 (1.7 g L^-1^) towards A4 (0.4 g L^-1^) (Table 1).

### RNA sequencing

Each sample was paired-end sequenced, obtaining around 20 million reads per library, with an average length of 71 nucleotides per read processed, discarding an average of 2% of total reads per library (S1 Table). Transcriptome coverage was estimated in 46X, with an average Pearson correlation about 94% between technical replicates. About 90% of reads were correctly mapped against Thompson Seedless reference genome [24].

### Gene expression analysis

**Fig 2:**
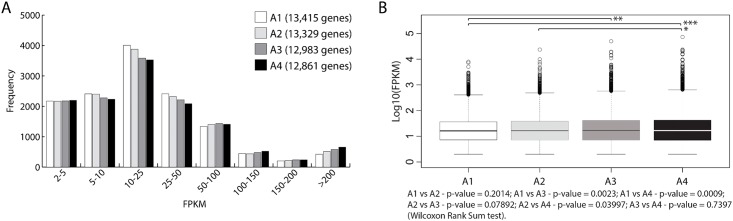
Transcript abundance measurements at each selected ripening stage. (A) The frequency represent the number of genes per category according to RPKM expression value. A1 (white bar), A2 (light gray bar), A3 (dark gray bar) and A4 (black bar). The number of total considered expressed genes (RPKM > 2) for each moment is presented in brackets. (B) Box plot representation of expressed genes of each library. Statistical analysis was performed (Wilcoxon Rank Sum Test). p-Value for all possible paired comparisons is presented at bottom of figure. Significant differences are represented by * (p < 0.05), ** (p < 0.01), *** (p < 0.001).

In terms of gene expression level, about 50% of annotated genes in the reference genome obtained a FPKM > 2 in each evaluated instance. Of these genes, more than 60% obtained FPKM values < 25, and about 30% obtained a value of FPKM between 10 and 25 (Fig 2A). The group of genes that showed the highest expression value (FPKM values > 200) account for about 5% of the genes. In general, the four conditions displayed the same trend regarding gene expression levels. Some of the differences were found mostly among A1-A2 vs A3-A4 at 25 FPKM and over 200 FPKM (Fig 2A). In fact, when assessing the data distribution, significant differences were detected between A1 vs A3, A1 vs A4 and A2 vs A4 (Fig 2B).

**Fig 3:**
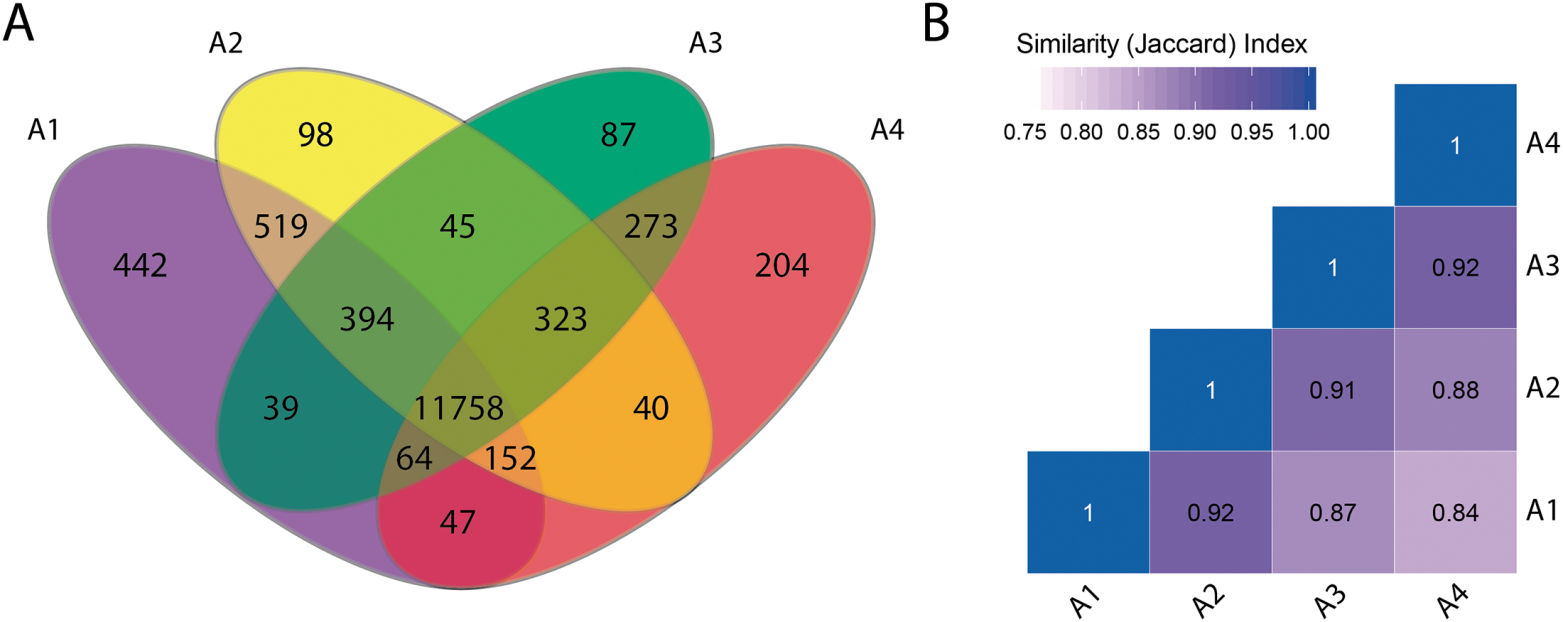
Gene expression distribution between selected moments evaluated. (A) Venn diagram of expressed genes (FPKM > 2) for each library (A1: 13,415 genes; A2: 13,329 genes; A3: 12,983 genes; A4:12,861 genes). The number of common expressed genes in each intersection area is presented. (B) Similarity analysis between four transcriptomic library. Jaccard index for each paired comparison.

We next analyzed the gene expression distribution among the four moments evaluated. From the universe of 14,480 genes with a FPKM > 2 in at least one instance studied, 11,758 (81%) of genes were expressed in the four stages, while a minor fraction of them were expressed only in one condition (3.1% in A1, 0.7% in A2, 0.6% in A3, and 1.4% in A4) (Fig 3A). Higher similarity index was obtained between consecutives instances, decreasing proportionally to the time differences between them (Fig 3B).

### Clustering analysis and Gene Ontology (GO)

**Fig 4:**
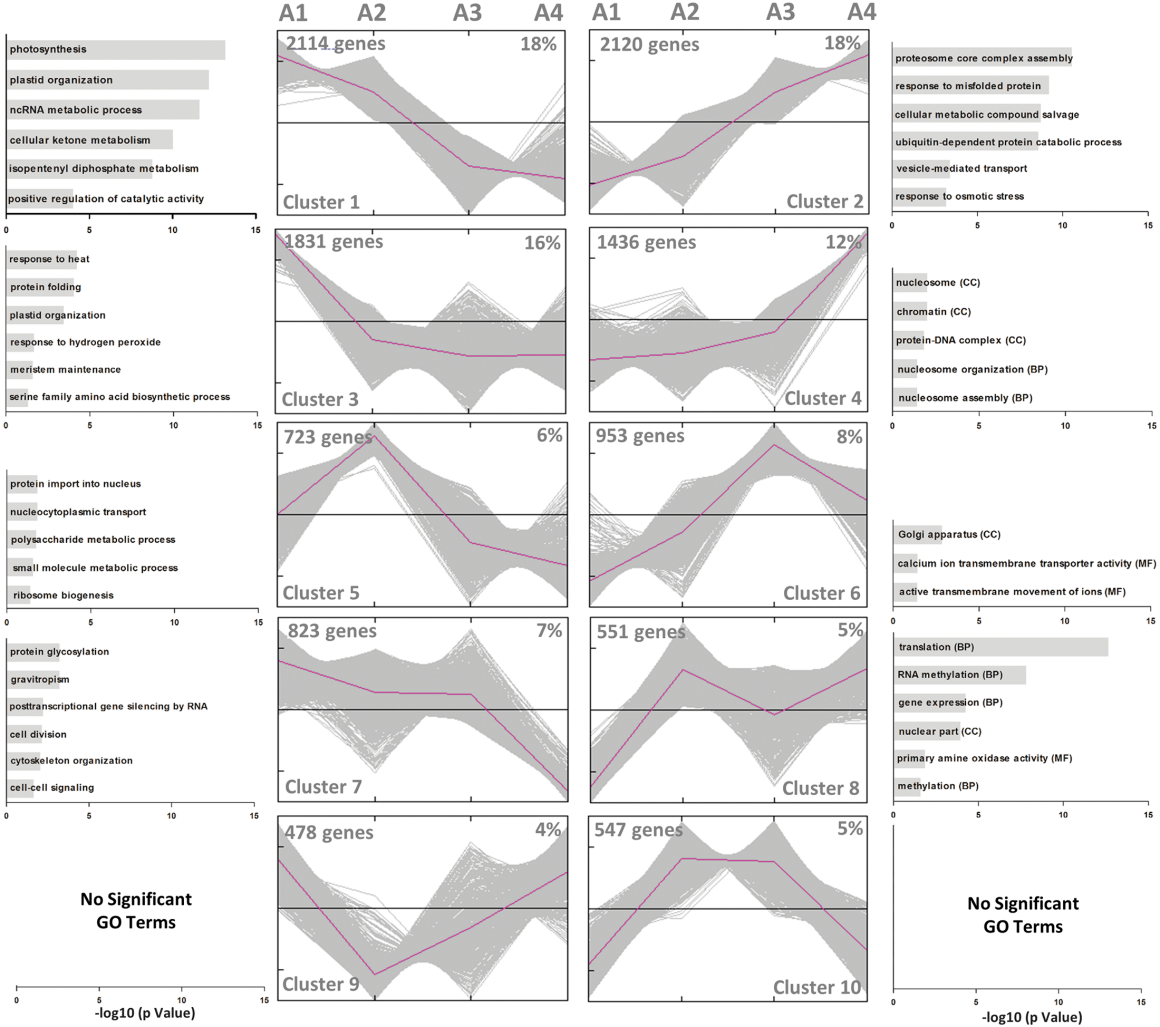
Clustering analysis according gene expression profiles in each selected moment. The number of genes and their respective percentage of the total considered in this analysis (11,576 genes) is presented at top of each graph. The representative profile of each cluster is represented in color pink and expression valued were normalized according to k-means clustering method (Y axis represent z-score). A Gene Ontology enrichment analysis was performed for each genes cluster. Results of significant terms is presented next to each graph.

One of the most valuables information obtained from transcriptome studies emerges from the functional analysis. In this regard, significant differential expressed genes were grouped into 10 clusters (Fig 4), according to their expression profile using *K*-means method [30] and GO enrichment analysis was performed. No significant GO terms were associated to cluster 9 (478 genes, 4%) and cluster 10 (547 genes, 5%), discarding those genes for further analysis (Fig 4).

**Fig 5:**
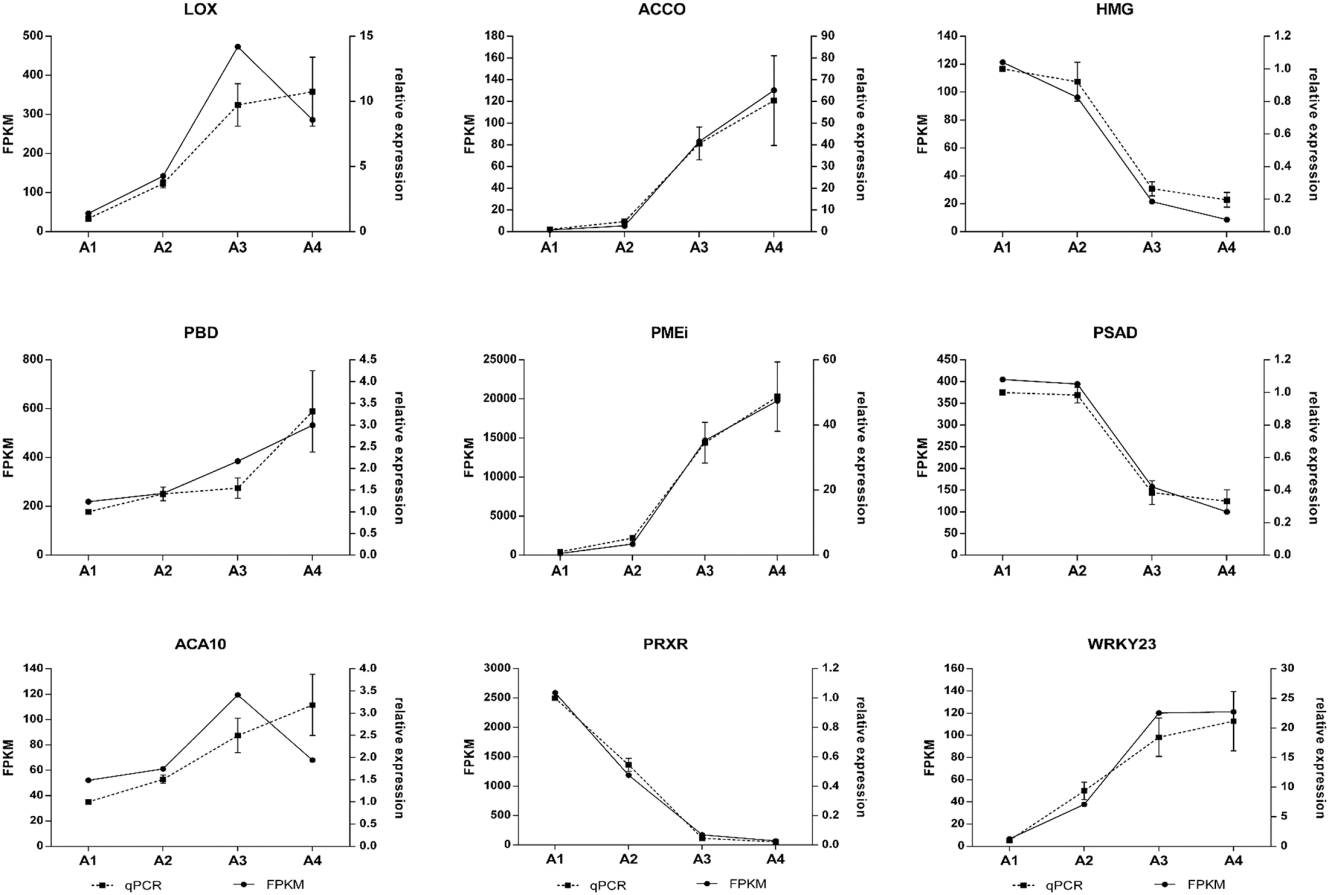
Transcriptome validation by qRT-PCR. Nine genes were selected according their biological role in the cell metabolism belong from different profile of expression in order to confirm the expression values obtained by RNASeq. For each gene, the FPKM value (continue line) and relative expression to A1 of qRT-PCR (dashed line) was evaluated in all selected points, using the expression of MVK gene for normalization. Vertical bars represent standard error of the mean of three biological replicates for qRT-PCR.

Cluster 1 grouped 2,114 (18%) genes showing a linear decrease in transcript accumulation, while cluster 2 showed the opposite profile, grouping 2120 (18%) genes with increase accumulation of transcript from A1 to A4 (S3 Table). Cluster 1 obtained the highest number of GO terms significantly associated compared to the other clusters (221 GO terms, FDR <0.05). Among the most significant terms are found “photosynthesis” (GO:0015979, 101 genes), “plastid organization” (GO:0009657, 103 genes), “positive regulation of catalytic activity” (GO:0043085, 34 genes), “isopentenyl diphosphate metabolic process” (GO:0046490, 67 genes), “cellular ketone metabolic process” (GO:0042180, 258 genes) and “ncRNA metabolic process” (GO:0034660, 98 genes) (Fig 4). Two members of this cluster coding for the subunit D-2 of photosystem I (PSAD, GSVIVG01017944001) and a peroxidase superfamily protein (PRXR, GSVIVG01012727001) had their expression patterns confirmed by qRT-PCR (Fig 5).

Cluster 2 grouped significantly 72 GO terms (S3 Table), suggesting a gradual increase from A1 to A4 of transcripts related to “response to osmotic stress” (GO:0006970, 96 genes), “vesicle-mediated transport” (GO:0016192, 81 genes), “ubiquitin-dependent protein catabolic process” (GO:0006511, 72 genes), “proteasome core complex assembly” (GO:0080129, 48 genes), “response to misfolded protein” (GO:0051788, 53 genes) and “cellular metabolic compound salvage” (GO:0043094, 52 genes) (Fig 4 and S3 Table). Cluster 3 was the third largest group with 1,831 genes (16%), grouping genes exhibiting a high accumulation of transcripts in A1 and then decreased at A2 remaining relatively constant up to A4. Among GO terms associated to this cluster were “plastid organization” (GO:0009657, 66 genes), "meristem maintenance" (GO:0010073, 38 genes), "protein folding" (GO:0006457, 55 genes), “RNA processing” (GO:0006396, 99 genes), and "response to hydrogen peroxide" (GO: 0042542, 63 genes) (Fig 4 and S3 Table). Two members of this cluster coding for the 20S proteasome beta subunit D1 (PBD, GSVIVG01001182001) and a plant invertase/pectin methylesterase inhibitor superfamily protein (PME1, GSVIVG01018598001) had their expression patterns confirmed by qRT-PCR (Fig 5).

The cluster 4 grouped 1,436 genes equivalent to 12% of total expressed genes, grouping genes increasing the abundance of transcript at the final A4 stage. Five GO terms were significantly associated to the related to biological processes of nucleosome organization (GO:0034728 and GO:0006334, 12 genes), grouping several histones, DNA-complex and chromatin related proteins (Fig 4 and S3 Table). 723 genes (6%) were grouped in cluster 5 showing an expression profile with a peak of increased accumulation of transcripts in A2 and A3. Four GO terms were significantly associated with this cluster, grouping 35 genes associated to "protein import into nucleus" (GO:0006606) and "ribosome biogenesis" (GO:0042254); "small molecule metabolic process" (GO:0044281, 131 genes), and "polysaccharide metabolic process" (GO:0005976, 38 genes) (Fig 4 and S3 Table). Cluster 6 grouped 953 genes (8%) showing a profile of increase expression peaking at A3 and declining to A4. GO analysis showed a significant association of 56 genes with the cellular component "Golgi apparatus" (GO: 0005794), and 10 genes associated to "ATPase activity, coupled to transmembrane movement of ions" (GO:0042625) grouping mainly calcium transporters (Fig 4 and S3 Table).

GO term enrichment analysis of cluster 7 showed a high number of significant GO terms associated (82 terms, S3 Table), grouping 823 genes (7%) with a significant decrease of transcript accumulation at A4. Among the most significant terms were found "regulation of gene expression, epigenetic" (GO:0040029, 33 genes); "production of ta-siRNAs involved in RNA interference" (GO:0010267, 14 genes); "helicase activity" (GO:0004386, 21 genes); “post-translational protein modifications” (GO:0043687, 82 genes); “protein amino acid glycosylation” (GO:0006486, 25 genes); "gravitropism" (GO:0009630, 21 genes,); "Golgi vesicle transport" (GO:0048193, 23 genes); and "reproductive structure development" (GO:0048608, 73 genes) (Fig 4). 551 genes (5%) were grouped in cluster 8, showing an increase in transcript abundance after A2 in a non-linear pattern, with 39 GO terms significantly associated. Among the most relevant terms founded were "gene expression" (GO:0010467, 109 genes); "translation" (GO:0006412, 47 genes); "RNA methylation" (GO:0001510, 23 genes); and "amine oxidase activity" (GO:0008131, 5 genes) (Fig 4). No significant GO terms were associated to cluster 9 (478 genes, 4%) and cluster 10 (547 genes, 5%).

### Metabolic pathways analysis

**Fig 6:**
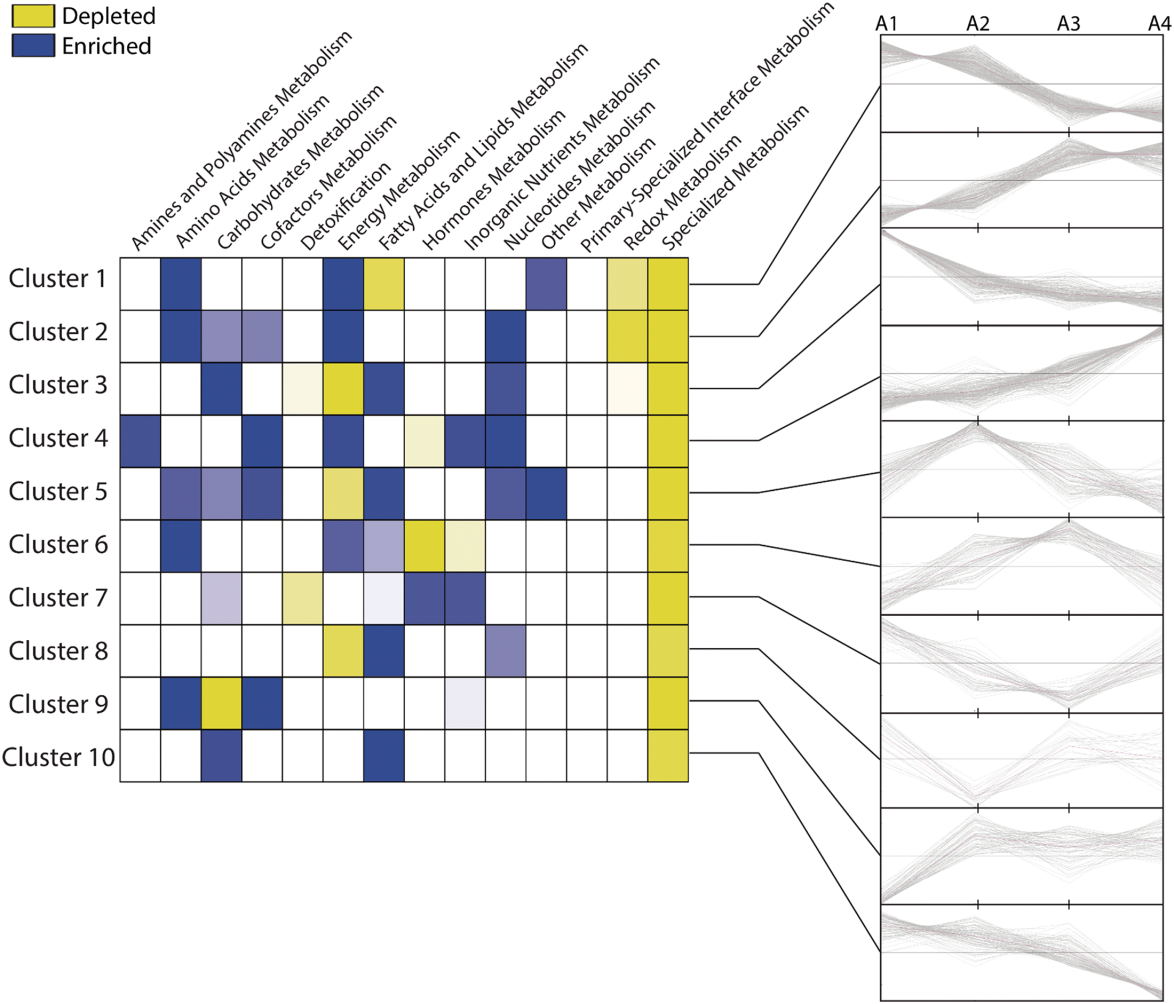
Expression analysis of genes associated to metabolic pathways. The analysis contempt 1,372 genes differentially expressed in at least one comparison considering A1 as a reference (Baggerley test, FDR < 0.05). The expression profiles of these genes encoded enzymes were clustered in 10 groups and an enrichment analysis of metabolic domains was performed for each one. Significant differences (Hypergeometric test, FDR < 0.05) are presented in blue (enrichment) / yellow (depleted) color scale.

Of the total of 25,885 genes annotated in the genome of the Thompson Seedless genome, 7,567 (29%) encode enzymes, of which 3,426 (13%) are associated to metabolic pathways [Plant Metabolic Network (PMN), http://plantcyc.org/, August 5, 2016] [36]. The remaining "orphan reactions", have not yet been associated with any particular pathway. In this study, 1,372 genes were associated with metabolic pathways and obtained significant differential expression (Baggerley test, FDR < 0.05) in at least one of the following comparisons: A2 vs A1; A3 vs A1 and/or A4 vs A1. The expression profiles of these 1,372 genes were grouped into 10 clusters (Fig 6) using *K*-means method [30]. 77% of the genes involved in metabolic pathways were associated to clusters 1, 2, 3 and 4, and 23% were grouped to clusters 5, 6, 7 and 8. The largest number of genes related to metabolic pathways were grouped in cluster 1 (259 genes, 19%). An enrichment analysis of metabolic domains [37] was performed showing differences between clusters (Fig 6). A significant enrichment of metabolism of amines, cofactors and inorganic nutrients could be associated with an increased expression profile during ripening (clusters 2 and 4), while an enrichment of metabolism of carbohydrates and fatty acids were associated with a down-regulated profile (clusters 1 and 3) (Fig 6).

To characterize more precisely the metabolic processes associated to each condition assessed, a pathways enrichment analysis from each significant metabolic domain was performed. Cluster 1 showed an over-representation of pathways involved in the synthesis of arginine and histidine, and the route of nitric oxide (Table 2). Also, cluster 1 showed an over-representation of genes associated to glycolysis, the pentose phosphate pathway and the route known as "rubisco shunt" described as a source generation of Acetyl-CoA independent from the Calvin Cycle [38] (S1 Fig). Genes involved in zeaxanthin synthesis and pathways involved in its interconversion in antheraxanthin and violaxanthin were also detected. Finally, an overrepresentation of the tRNA charging pathway was also found associated with cluster 1, obtaining the most statistically significate value (Table 2). The cluster 2 grouped the largest number of metabolic pathways significantly overrepresented compared to other clusters, which were mainly involved in synthesis of amino acids, carbohydrates and energy metabolism (Table 2). The route of the plastid and cytosolic glycolysis was significantly associated to cluster 2, in addition to alternative oxidase pathway, Krebs cycle and rubisco shunt. Also several routes of degradation of macromolecules, such as aspartate, uracil, galactose and stachyose were also recorded (S2 Fig).

**Table 2:** Metabolic pathway enrichment analysis in each expression profile cluster.

Cluster	Metabolic Domain	Metabolic Pathway	Description	p-Value (-log_10_)
1	Amino Acids	ARGSYNBSUB-PWY	arginine biosynthesis II (acetyl cycle)	2.02 ***
1	Amino Acids	GLUTORN-PWY	ornithine biosynthesis	1.12 *
1	Amino Acids	HISTSYN-PWY	histidine biosynthesis	1.12 *
1	Amino Acids	PWY-4983	citrulline-nitric oxide cycle	1.14 *
1	Energy	GLYCOLYSIS	glycolysis	1.03 *
1	Energy	OXIDATIVEPENT-PWY	pentose phosphate pathway (oxidative branch) I	1.35 **
1	Energy	PWY-5723	Rubisco shunt	1.67 **
1	Energy	PWY-5944	zeaxanthin biosynthesis	1.66 **
1	Energy	PWY-5945	zeaxanthin, antheraxanthin and violaxanthin interconversion	1.22 *
1	Other	TRNA-CHARGING	tRNA charging	2.37 ***
2	Amino Acids	ASPARTATE-DEG1-PWY	aspartate biogénesis/degradation	1.64 **
2	Amino Acids	PWY-3982	uracil degradation I (reductive)	1.20 *
2	Carbohydrate	GLUCONEO-PWY	gluconeogenesis I	1.61 **
2	Carbohydrate	PWY-3821	galactose degradation III	2.00 **
2	Carbohydrate	PWY-4661	1D-myo-inositol hexakisphosphate biosynthesis III	1.65 **
2	Carbohydrate	PWY-4841	UDP-α-D-glucuronate biosynthesis (from myo-inositol)	1.32 **
2	Carbohydrate	PWY-6317	galactose degradation I (Leloir pathway)	1.25 *
2	Carbohydrate	PWY-6527	stachyose degradation	1.15 *
2	Energy	GLYCOLYSIS	glycolysis	2.90 ***
2	Energy	PWY-1042	glycolysis IV (plant cytosol)	3.62 ***
2	Energy	PWY-4302	aerobic respiration III (alternative oxidase pathway)	1.06 *
2	Energy	PWY-5690	TCA cycle II (plants and fungi)	1.36 **
2	Energy	PWY-5723	Rubisco shunt	1.37 **
3	Amino Acids	HOMOSERSYN-PWY	homoserine biosynthesis	1.23 *
3	Amino Acids	METHIONINE-DEG1-PWY	methionine degradation I (to homocysteine)	1.06 *
3	Amino Acids	PWY-4361	S-methyl-5-thio-α-D-ribose 1-phosphate degradation	1.23 *
3	Carbohydrates	PWY-5800	xylan biosynthesis	3.19 ***
3	Carbohydrates	PWY-621	sucrose degradation III (sucrose invertase)	1.82 **
3	Fatty Acids and Lipids	PWY0-1264	biotin-carboxyl carrier protein assembly	1.94 **
3	Fatty Acids and Lipids	PWY-2501	fatty acid α-oxidation I	1.88 **
3	Fatty Acids and Lipids	PWY-5669	phosphatidylethanolamine biosynthesis I	1.10 *
4	Amino Acids	PWY-4321	glutamate degradation IV	1.49 **
4	Energy	PWY-4302	aerobic respiration III (alternative oxidase pathway)	5.35 ***
4	Energy	PWY-5690	TCA cycle II (plants and fungi)	2.06 ***
4	Inorganic Nutrients	PWY-6348	phosphate acquisition	1.26 *
5	Fatty Acids and Lipids	PWY-4381	fatty acid biosynthesis initiation I	1.43 **
5	Fatty Acids and Lipids	PWY-6352	3-phosphoinositide biosynthesis	1.11 *
5	Fatty Acids and Lipids	PWY-6804	diacylglycerol biosynthesis (PUFA enrichment in oilseed)	1.12 *
6	Energy	PYRUVDEHYD-PWY	pyruvate decarboxylation to acetyl CoA	1.05 *
6	Fatty Acids and Lipids	TRIGLSYN-PWY	triacylglycerol biosynthesis	1.55 **
7	Amino Acids	PWY-6196	serine racemization	1.05 *
7	Cofactors	PWY-2161B	glutamate removal from folates	1.05 *
7	Inorganic Nutrients	PWY-5326	sulfite oxidation IV	1.09 *
8	Fatty Acids and Lipids	PWY-5148	acyl-CoA hydrolysis	1.11 *

* Hypergeometric test p Value < 0.1;

** p Value < 0.05;

***p Value < 0.01.

Eight metabolic pathways were significantly associated with cluster 3, highlighting routes related to carbohydrates metabolism such as degradation of sucrose (sucrose invertase) pathway and xylan synthesis. Also, pathways involved in the metabolism of fatty acids were significantly associated to cluster 3, such as biotin-carboxyl carrier protein assembly pathway and fatty acid α-oxidation I (Table 2 and S3 Fig). Four metabolic pathways were significantly associated to cluster 4, highlighting the Krebs cycle and the alternative oxidase pathway, obtaining this last route the most significant value of the entire analysis. Others pathways associated with cluster 4 were the glutamate degradation and the phosphate acquisition pathways (Table 2 and S4 Fig).

The remaining four clusters (clusters 5, 6, 7 and 8) share the characteristics of having non-linear expression profiles presenting increase/decrease peaks at intermediate points, and less to 100 genes each (Fig 6). A total of nine metabolic pathways were significantly associated to these clusters (S5, S6, S7 and S8 Figs). Five of these routes were related to fatty acids metabolism, which three of them belong to cluster 5 showing a peak of expression at A2. Other routes represented in these clusters were pyruvate decarboxylation to acetyl CoA (cluster 6), serine racemization, glutamate removal from folate and sulfite oxidation IV (cluster 7) (Table 2).

## Discussion

### Phenotype analysis

Fruit ripening has been extensively studied in climacteric fruit, being characterized by a respiration burst paralleled by an increase in ethylene production. In non-climacteric fruits such as grapes, the ripening would take place without such pronounced respiration and ethylene burst. However, studies in grapes related with respiratory metabolism suggest important changes in metabolism and glycolysis activity during ripening of Thompson Seedless grape berries, despite the lack of increase in respiration rate typical of climacteric fruit [39]. Ethylene response during grape development has also been reported, including an increased expression of ethylene biosynthesis related genes in Thompson Seedless at veraison [8–10]. These findings highlight the need to further characterize non-climacteric fruit ripening. To contribute to the knowledge of this process, we performed a transcriptome analysis during ripening of table grapes berries cv Thompson Seedless. The analysis of firmness, diameter, soluble solids accumulation and acidity showed an expected behavior according to previously reported evidence for grape berries in Phase III [13]. Berries showed an increase in soluble solids and in diameter from A1 to A4 and a decrease in acidity of 70%, accompanied by a firmness loss of 65% (Fig 1). Thus, selected berries from A1 to A4 were representative of ripening process in grape bunches of Thompson Seedless cultivar, which were used for transcriptome analysis.

### Global transcriptome analysis

Our transcriptome analysis allowed identifying about 13,000 expressed genes (FPKM > 2) in each analyzed point, equivalent to the half of annotated genes in the Thompson Seedless genome [24]. The distribution of genes by expression level category showed a similar trend in the four analyzed points, with a major peak of genes expressed at 10–25 FPKM followed by a minor peak at over 200 FPKM (Fig 2A). However, it could be observed a gradual decrease in the proportion of genes of low expression range (FPKM < 50) accompanied by an increase in the proportion of genes in high expression range (FPKM > 200) from A1 to A4, generating statistically significant differences between the non-contiguous libraries (Fig 2B). The GO terms enrichment analysis shows an enrichment of shared biological processes in this group of highly expressed genes at the four instances analyzed (S2 Table), such as the response to abiotic stresses, particularly osmotic stress; protein synthesis, glucose metabolism and water transport. Regarding the differences, processes related to Golgi apparatus organization were presented in all points except A4. Also, enrichment of terms related with macromolecules recycling by proteasome were detected only at A4. This result, added to the fact that the total number of genes detected decreased from Al to A4 by 4%, could suggest a general cell metabolic shutdown during grapes fruit ripening.

The Venn diagram analysis showed that about 90% (11,758 genes) of the transcripts were present in all four points, while 10% (831 genes) of the genes showed an exclusive expression in one of the evaluated instances assessed (Fig 3). In agreement with statistical analysis of Fig 2B, similarity index between libraries decreases proportionally to timespan who separated them (Fig 3B). This result demonstrates the effectiveness in detect the transcriptome evolution during ripening, which lies in the saturation level of our analysis with four analysis points between veraison and harvest.

A great diversity of gene expression profiles was detected by clustering analysis. Approximately 65% of the genes showed a relatively linear expression profile between A1 and A4, as it was observed in the clusters 1 (continuous decrease) and 2 (continuous increase) (Fig 4); while about a third of the genes showed a non-linear expression profile, with partial expression peaks associated to particular points (clusters 5, 6, 7 and 8). To identify the association of the different expression profiles with specific biological functions, we used the GO annotation (http://www.geneontology.org). Our results were consistent with several mechanisms described during fruit ripening, which help to validate those novel terms associated to ripening (see below). Furthermore, metabolic pathway analysis was a relevant input to support these results.

### Transcriptome down-regulation during ripening

Within the group of genes that decreased their expression during ripening, the term "photosynthesis" was the most significant, grouping several genes related to chloroplast integrity. The greatest differences were related with proteins associated to light harvesting complex photosystem I and II (LHCP I and II). The decrease of photosynthetic activity and decreased expression and transcription of the photosystems associated proteins has been described in the literature as part of the ripening process in various fruits including grape [16,40,41]. The degradation of LHCP II is a finely coordinated process in parallel with degradation of chlorophyll-a due to the high phytotoxicity of the chlorophyll degradation intermediates [42,43]. Regarding to chlorophyll degradation pathway, our results suggests a constant expression of transcripts encoding chlorophylase, pheophorbide-a oxygenase and RCC reductase (S9 Fig). The pheophorbide-a oxygenase obtained the highest expression values, coinciding with it described key step role in the chlorophyll degradation pathway [44]. It is noteworthy that most genes associated with photosynthesis, including chlorophyll degradation pathway, had associated transcripts even at A4. The presence of small amounts of transcripts and proteins associated with photosynthesis have been previously reported during late stage of tomato ripening [40]. However, it remains unknown whether this finding involves *de-novo* synthesis or some mechanism associated to protein turn-over.

Another significant process that decreased its transcriptional activity during ripening was the isoprenoid metabolism. Isoprenoids (or terpenoids) are one of the most diverse groups of metabolites in plants, participating in several essential processes such as respiration, photosynthesis, growth and development [45]. Within this group, the gene coding for the enzyme 3-hydroxy-3-methylglutaryl-CoA reductase (HMG1), a key enzyme that catalyzes the first step in the synthesis of cytosolic isoprenoids, showed a significant decrease during ripening which was also confirmed by qRT-PCR (Fig 5). HMG1 mutants showed a phenotype of early senescence in *A*. *thaliana* [46], with a possible role in the endoplasmic reticulum morphogenesis [47]. The down-regulation of terpenoids pathways suggest an additional input for ripening control and a putative crosstalk with senescence mechanism. Other important members of isoprenoid correspond to carotenoid compounds. One of the main functions attributed to these pigments corresponds to photo-protection through the dissipation of light energy excess absorbed by the complex antenna in the chloroplasts during photosynthesis [48,49]. In this regard, metabolic pathway analysis suggests a high expression of zeaxanthin synthesis and its violaxantin conversion in the early stages of ripening, declining after during ripening progress (Table 2). Thus, the analysis revealed the decreased expression of the 3 isoforms predicted in *V*. *vinifera* of b-carotene hydrolase which converts b-carotene to zeaxanthin (EC 1.14.13.129). Regard the conversion of zeaxanthin to violaxanthin, a great abundance of one of the four transcripts isoforms predicted for zeaxanthin epoxidase (EC 1.14.13.90) through all ripening was detected, while violaxanthin de-epoxidase, which catalyzes the reverse reaction, was not detected (EC 1.10.99.3) (S1 Fig). Thus, our data suggest that b-carotene to violaxantin conversion is favored during ripening of Thompson Seedless grapes cultivar. This result is in concordance with findings in grape wine, where unlike that what has been seem in other fruits, the carotenoids decreased during ripening while xanthophylls increased, specially violaxanthin [50,51]. This effect could be associated with a protection mechanism of non-photochemical quenching generated by the over exposition to light of fruit in conditions of disassemblement of photosynthetic machinery across ripening.

### Transcriptome up-regulation during ripening

One of the most relevant processes associated to transcriptional increase during ripening correspond to macromolecules recycling, highlighting the proteasome complex assembly process grouping 47 genes (S3 Table). Consequently, the response to misfolded proteins and protein ubiquitination were also important terms in this cluster. These processes allow a significant protein turnover control during grape ripening, where proteolysis mediated by ubiquitination could be playing an important role on its modulation [52]. Experiments in *A*. *thaliana* shows that the F-box protein ORE9 selectively controls the ubiquitination and proteolysis of senescence repressor elements [53]. Our data indicates a gradual increase in the expression of ORE9 during ripening, suggesting the influence of this mechanism during the ripening of Thompson Seedless grape berries.

The lipid metabolism was another significantly represented process that increased their expression during ripening. The degradation of lipid membranes, especially the thylakoid membranes is an important source of energy during the process of senescence in plants [54]. Four key enzymes are involved in this process: phospholipase D, phosphatidic acid phosphatase, lipolytic acyl hydrolase and lipoxygenase, whose concerted activity has been associated with membrane degradation in senescent tissue [54,55]. The suppression of the isoforms α and δ of phospholipase D retarded the senescence promoted by ABA and ethylene in leaves of *A*. *thaliana* [56,57]. Our analysis showed a decrease in the expression of these isoforms, however phospholipase D-β showed an up-regulated profile during ripening. Additionally, an increased expression of phosphatidic acid phosphatase, lipoxygenase acyl hydrolase and lipoxygenase coding genes was detected, the last also confirmed by qRT-PCR (Fig 5). Consistent with this mechanism of energy recycling, the route of fatty acid b-oxidation in peroxisome has also been associated with senesce in leaves of *A*. *thaliana* [58]. The GO term "fatty acid beta-oxidation" was significantly associated with increased transcriptional profile of cluster 2, and the analysis of this metabolic pathway was also consistent with this up-regulation during ripening, especially for the enoyl-CoA hydratase (S9 Fig). The acetyl-CoA generated from phospholipids by this pathway can directly enter the Krebs cycle or could participate in the conversion of malate through the glyoxylate cycle and subsequent transformation of sucrose through gluconeogenesis [59]. Regarding glyoxylate cycle, our results revealed the absence of two key enzymes transcripts as malate synthase and isocitrate lyase [60]. On the other hand, the examination of the abundance of transcripts associated with control points of both glycolysis and gluconeogenesis pathways suggest a greater activity of glycolysis during the ripening. Thus, results showed an increase in the activity of different isoforms of invertase and a constant high expression of hexokinase through grape berry ripening. In addition, an increase of phosphofructokinase and pyruvate kinase transcripts was detected, which are described as irreversible reactions and main control points of glycolysis [59]. Meanwhile, only one isoform of pyruvate orthophosphate dikinase was detected which decrease during ripening, corresponding to the main checkpoint of gluconeogenesis. These results suggest the presence of an energy recycling mechanism from phospholipids fatty acids, similar to the one described on *A*. *thaliana* membranes of senescent leaves. However, in contrast to the mechanism described in leaves, results on grape suggest that this energy source is confined to support berry ripening rather than to be used for sucrose biosynthesis. This metabolic interpretation of transcriptional data is congruent with expected differences of a “sink” organ, such as fruit, compared to recycling metabolism of a “source” organ such as leaf during a senescence process.

Another process associated with an increased expression during ripening was the response to stress, especially osmotic stress. One of the most abundant transcript in this group corresponded to osmotin-34 belonging to the thaumatin superfamily proteins. Although the role of most of these superfamily proteins is unknown, its function is generally associated with stress response [61]. Experiment in tobacco suggests that the promoter region of osmotin gene act in coordination with other cis-elements involved in the response to ethylene [62]. Different members of WRKY and ERF transcription factors have been associated to diverse processes, including stress response and senescence [63–66]. In this respect, our results showed an increase of transcript accumulation of different members of WRKY family protein and ERF, highlighting the isoforms WRKY-23 and ERF-9, suggesting an important role of these transcription factors in the response to stress during grape berry ripening (WRKY-23 expression was confirmed by qRT-PCR, Fig 5). An increase in the transcription of genes associated with the organization of the nucleosome and chromatin condensation was also observed, especially at A4. These processes have been associated with late events of senescence as typical effect of PCD detected in leaf of several species [67].

One the key enzymes in ethylene metabolism is the ACC oxidase (ACCO), which catalyzes the last step of ethylene synthesis pathway [68]. We detected a significant increase in the expression of ACCO during ripening (a pattern confirmed by qRT-PCR, Fig 5). The substrate of this enzyme is catalyzed by ACC synthase, two of which were detected in low levels along ripening. Despite grape has been characterized as a non-climacteric fruit with an independent ethylene ripening, our results suggest an increase in transcriptional activity associated with ethylene synthesis pathway. The synthesis pathway and ethylene signaling has been poorly described in grape, however differential effects associated to ripening by applications of the ethylene receptor blocker 1-MCP have been reported [69]. Additionally, it has been reported differential expression of various genes associated with ethylene biosynthesis in Thompson Seedless during ripening [8]; and in Cabernet Sauvignon cultivar has been detected a differential expression of genes related with water transport, cell wall metabolism and transcription factors in response to ethylene application [9,10]. Accordingly, our results also showed the increase in transcript accumulation of some ethylene response transcription factors during ripening, indicating a possible influence of ethylene over the ripening control in non-climacteric fruit.

### Others transcriptional profiles

Approximately 35% of the genes displayed a non-linear transcriptional behavior during ripening, with partial peaks of increases and/or decrease in their abundance of transcripts during ripening, such as observed in the expression profiles of the clusters 5 to 10. Given the diversity of expression profiles, there was a smaller amount of both GO terms and metabolic pathways associated to these clusters. Cluster 5 displayed a transcriptional peak at A2 associated to "polysaccharide metabolic process". This term group several genes involved in cell wall polysaccharides metabolism, which could be related to higher rate of firmness loss observed at this point. Other terms associated to cluster 5 were "nuclear import" and "ribosome biogenesis" grouping several ribosomal genes, many of which remained at high transcript levels up to A4. Ribosomal RNA has been proposed as an important source of nitrogen source, carbon and phosphorus derived from the catabolism of nucleic acids during senescence, registering an abundance loss of rRNA as this process progresses, while the DNA remains constant [70,71].

Additionally, the expression analysis of cluster 5 suggests a decrease in fatty acid synthesis after A2 for the synthesis of membrane phospholipids. This result is consistent with our previously suggested hypothesis related to metabolic shift of ripening cells to promote the synthesis of acetyl-CoA as a substrate for the Krebs cycle rather than the generation of membrane phospholipids. Another interesting metabolic pathway associated to cluster 5 corresponded to the 3-phosphoinositide synthesis which play an important role as second messenger [72,73]. The gene expression analysis of this route showed a preference at the transcriptional level to generate the compound phosphatidylinositol 4,5-bisphosphate, which has been associated with response to vesicle trafficking and polarity growth through regulation of the cytoskeleton in pollen tube [74,75] (S5 Fig).

Cluster 6, which presented a transcriptional peak at A3, showed an association with the terms "calcium ion transmembrane transporter activity", grouping five transmembrane calcium pumps (ACA10, ECA3, ECA4, calcium ATPase 2 and 4) predicted in plasma membrane, reticulum and vacuoles. The expression profile of one these genes was confirmed by qRT-PCR, showing an increase of transcript accumulation of ACA10 until harvest (Fig 5). The term "Golgi apparatus" was also associated to this cluster, grouping several genes related to cell wall polysaccharides metabolism such as pectin methyl esterase, methyltransferases, xylose isomerase, polygalacturonase inhibiting protein and xyloglucan endotransglycosylase, among others. The expression of these genes suggests a high transcriptional activity at A3 related to the cell wall metabolism, accompanied by a putative peak in the movement of calcium into the apoplast that could interact with pectins, modifying the cell wall structure. These results are consistent with biochemical evidence from grape berries cv Thompson Seedless, suggesting an important influence of calcium bridges in maintaining the cell wall pectin structure, especially close to the harvest time [21]. Besides, it has also been extensively described the role of calcium in the regulation of signaling pathways involved in PCD in plants [76]. The analysis of metabolic pathways associated with the cluster 6 showed an increase in the transcription of the genes coded for pyruvate decarboxylation to acetyl CoA activity, consistent with the increased flow of central metabolism from glycolysis to the Krebs cycle during ripening (S6 Fig). Moreover, the triacylglycerol synthesis pathway shows high transcriptional activity during the ripening, suggesting a highly coordinated regulation of lipid homeostasis over ripening, as discussed previously.

The cluster 7 grouped genes with a significant decrease at A4 of transcript accumulation. One of the most significant terms associated with this cluster was "protein glycosylation" which correspond to one of most widely described post-translational modifications occurring in the ER and Golgi [77]. The term "post-transcriptional gene silencing by RNA" was also associated with cluster 7, grouping several genes related to post-transcriptional gene regulation such as RNA helicase, splicing factors, P-loop hydrolases, Dicer-like protein, ARM and HEAT repeat containing proteins involved in the regulation of micro-RNA activity, among others. These results suggest a decrease in the post-transcriptional and post-translational metabolism of proteins at A4, suggesting a cell metabolic depression taking place after A3. Other terms highly associated with cluster 7 were "gravitropism", "cytoskeleton organization" and "cell division". These genes are associated with structural proteins of the Golgi apparatus, endomembranes systems, microtubule-associated proteins and some DNA binding proteins related with cytokinesis and polarized growth gravitropism studied in the *Arabidopsis* root apex [78,79]. The polarization effect of cell growth is a known process due to the application of growth regulators such as auxin and gibberellins in plants [80]. Also, a differential effect in the form of berries due to the effect of growth regulators GA3 and CPPU has been observed in table grapes [81]. These results suggest a cell growth polarization effect associated to GA3 treatment, following a similar molecular mechanism to cell elongation gravitropism described in the *Arabidopsis* root apex [78]. In this regard, our results suggest a decrease in transcriptional activity associated to polarized cell elongation from A3 to A4, which matches the absence of changes registered in the berry diameter measurements between 72 and 78 dpa (Table 1).

Results from cluster 8 suggest a negative association between the terms "gene expression" with the term "translation" according to the ontological prediction by AgriGO. This result supports the evidence of gene expression down-regulation at A4 compared to early ripening. An enrichment of the term "RNA methylation" was also found, grouping several genes encoding for rRNA and mRNA adenosine methylase (MTA). The *N*^6^-methyladenosine is a modification of mRNA involved in the specific recognition of proteins that affect its translation and lifespan [82]. While it is not fully understood the role of methylation of mRNA, there is evidence in *Arabidopsis* that MTA interacts with a specific splicing factor [83]. Our data suggest the constant expression of this MTA protein from A1 to A4, suggesting the involvement of this transcriptional regulation mechanism during the whole grape berry ripening process.

In order to confirm the RNASeq expression data, qRT-PCR experiments were carried out in nine representative genes of transcriptional profile diversity, considering also their biological role in the cell metabolism (Fig 5). The high correlation in gene expression profiles obtained by both methods allowed validate transcriptome expression analysis.

**Fig 7:**
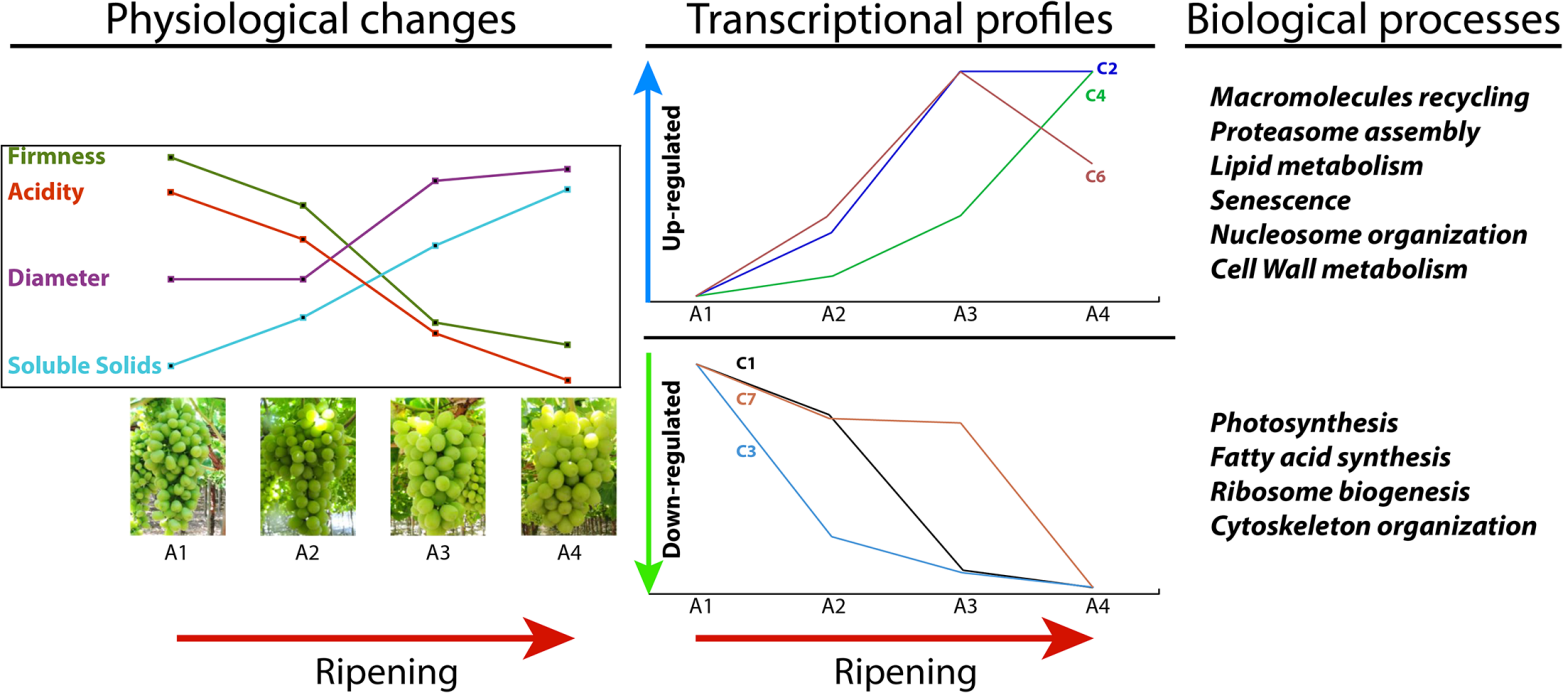
Most relevant biological processes involved during table grape berry ripening. In this schematic resume, the biological processes which show an up-regulated transcriptional profile are presented in red. In contrast, the processes presented a decrease of its related transcripts are presented in green.

Fig 7 summarizes the most relevant biological processes associated with transcriptional profiles showing increasing or decreasing trends during ripening. A visible correspondence between the main physiological changes with transcriptomic profiles was detected during these grape developmental stages. Differential expression associated with cytoskeleton organization and cell wall metabolism could be associated with changes observed in berry diameter and texture. The "sink" role of grape berry also correlated with a significant decrease of photosynthesis related genes, combined with an active process of energy recycling and the increase of specific isoforms of key enzymes of the sucrose biosynthesis pathway (S9 Fig).

## Conclusion

The functional analysis of transcriptome during grape berry ripening of Thompson Seedless cultivar, allowed the identification of several cellular processes described during senescence and PCD in plant cell, especially close to harvest. Despite the non-climateric characteristic of grape fruit, our results suggest an increase on the transcriptional activity of genes associated to respiration metabolic pathways across ripening. Our results are consistent with several cell mechanisms described during fruit ripening and allow to propose molecular mechanism and specific genes related with phenotypic and physiological changes registered during table grape berry ripening.

## Supporting information

S1 Fig Metabolic pathways associated to Cluster 1. (PDF)

S2 Fig Metabolic pathways associated to Cluster 2. (PDF)

S3 Fig Metabolic pathways associated to Cluster 3. (PDF)

S4 Fig Metabolic pathways associated to Cluster 4. (PDF)

S5 Fig Metabolic pathways associated to Cluster 5. (PDF)

S6 Fig Metabolic pathways associated to Cluster 6. (PDF)

S7 Fig Metabolic pathways associated to Cluster 7. (PDF)

S8 Fig Metabolic pathways associated to Cluster 8. (PDF)

S9 Fig Additional metabolic pathways. (PDF)

S1 Table Quality processing results of transcriptomic libraries. (XLSX)

S2 Table Gene Ontology analysis in overexpressed genes. (XLSX)

S3 Table Gene Ontology analysis in each cluster. (XLSX)
