# Supplementary material for: Transcriptome analysis during ripening of table grape berry cv. Thompson Seedless
Source: PLoS One. 2018 Jan 10;13(1):e0190087. doi: 10.1371/journal.pone.0190087 (PMC5761854; doi:10.1371/journal.pone.0190087)
Supplement: S1 Fig — (PDF) [file pone.0190087.s001.pdf]

# Pathway: arginine biosynthesis II (acetyl cycle)

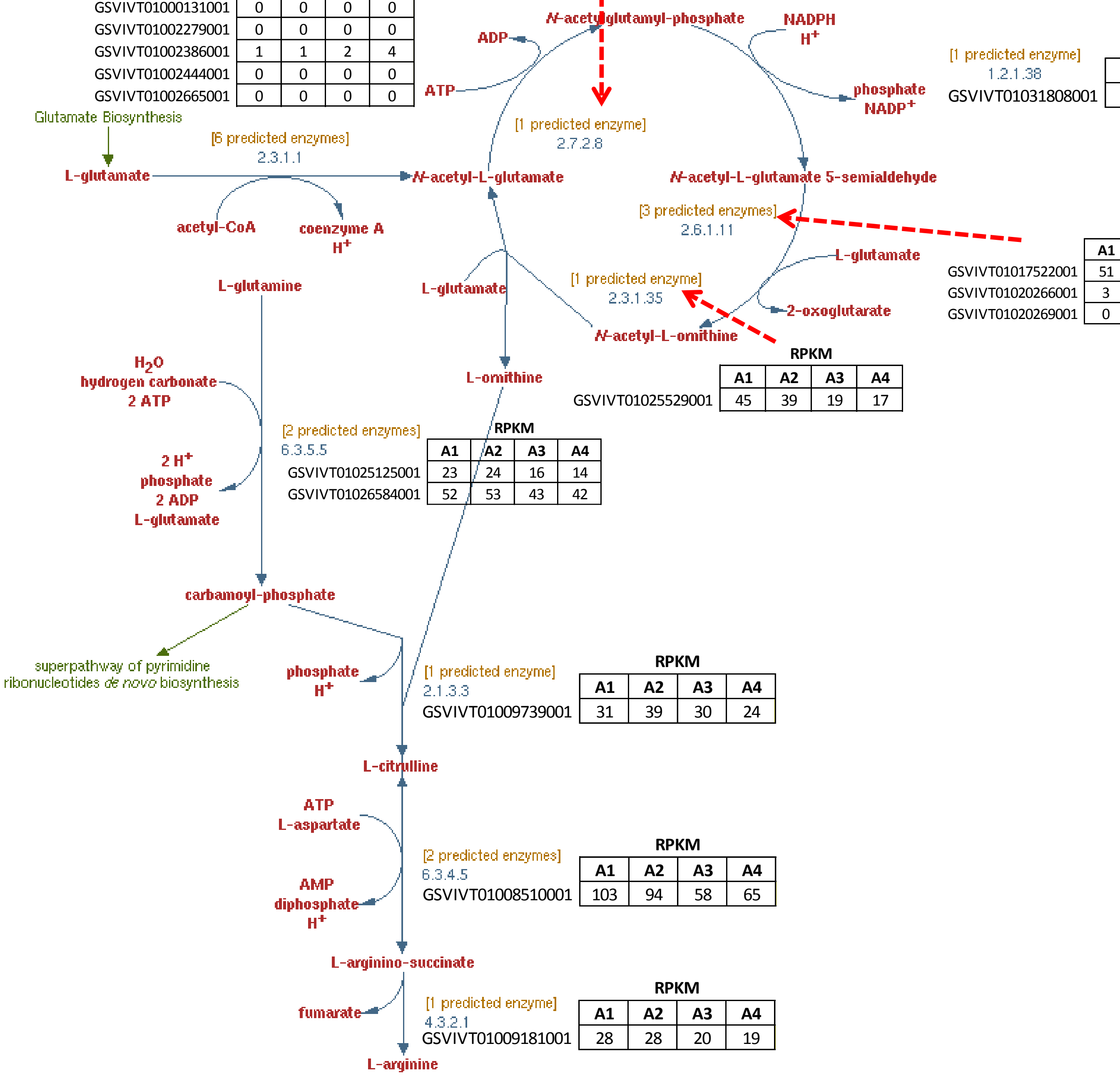


RPKM (2.3.1.1)

| | A1 | A2 | A3 | A4 |
|---|---|---|---|---|
| GSVIVT01000131001 | 0 | 0 | 0 | 0 |
| GSVIVT01002279001 | 0 | 0 | 0 | 0 |
| GSVIVT01002386001 | 1 | 1 | 2 | 4 |
| GSVIVT01002444001 | 0 | 0 | 0 | 0 |
| GSVIVT01002665001 | 0 | 0 | 0 | 0 |

RPKM (2.7.2.8)

| | A1 | A2 | A3 | A4 |
|---|---|---|---|---|
| GSVIVT01035586001 | 32 | 27 | 18 | 16 |

RPKM (1.2.1.38)

| | A1 | A2 | A3 | A4 |
|---|---|---|---|---|
| GSVIVT01031808001 | 42 | 41 | 28 | 28 |

RPKM (2.6.1.11)

| | A1 | A2 | A3 | A4 |
|---|---|---|---|---|
| GSVIVT01017522001 | 51 | 44 | 38 | 37 |
| GSVIVT01020266001 | 3 | 3 | 2 | 2 |
| GSVIVT01020269001 | 0 | 0 | 0 | 0 |

RPKM (2.3.1.35)

| | A1 | A2 | A3 | A4 |
|---|---|---|---|---|
| GSVIVT01025529001 | 45 | 39 | 19 | 17 |

RPKM (6.3.5.5)

| | A1 | A2 | A3 | A4 |
|---|---|---|---|---|
| GSVIVT01025125001 | 23 | 24 | 16 | 14 |
| GSVIVT01026584001 | 52 | 53 | 43 | 42 |

RPKM (2.1.3.3)

| | A1 | A2 | A3 | A4 |
|---|---|---|---|---|
| GSVIVT01009739001 | 31 | 39 | 30 | 24 |

RPKM (6.3.4.5)

| | A1 | A2 | A3 | A4 |
|---|---|---|---|---|
| GSVIVT01008510001 | 103 | 94 | 58 | 65 |

RPKM (4.3.2.1)

| | A1 | A2 | A3 | A4 |
|---|---|---|---|---|
| GSVIVT01009181001 | 28 | 28 | 20 | 19 |

# Pathway: ornithine biosynthesis

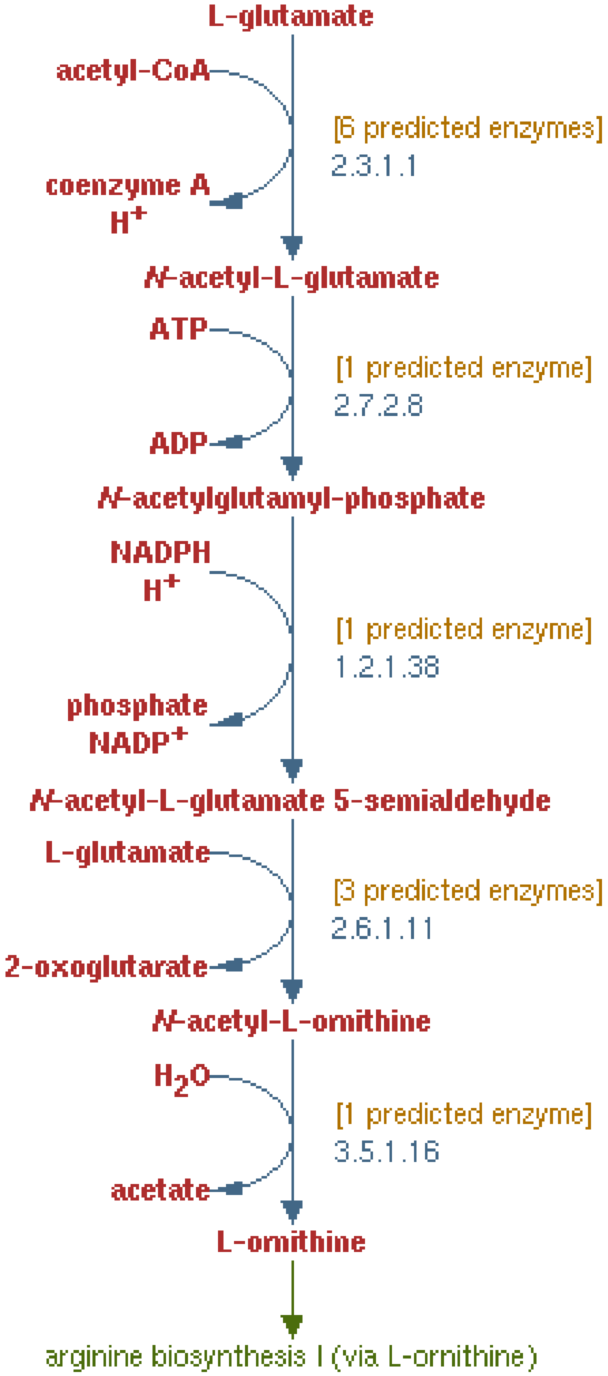


RPKM

| | A1 | A2 | A3 | A4 |
|---|---|---|---|---|
| GSVIVT01000131001 | 0 | 0 | 0 | 0 |
| GSVIVT01002279001 | 0 | 0 | 0 | 0 |
| GSVIVT01002386001 | 1 | 1 | 2 | 4 |
| GSVIVT01002444001 | 0 | 0 | 0 | 0 |
| GSVIVT01002665001 | 0 | 0 | 0 | 0 |
| GSVIVT01025529001 | 45 | 39 | 19 | 17 |

RPKM

| | A1 | A2 | A3 | A4 |
|---|---|---|---|---|
| GSVIVT01035586001 | 32 | 27 | 18 | 16 |

RPKM

| | A1 | A2 | A3 | A4 |
|---|---|---|---|---|
| GSVIVT01031808001 | 42 | 41 | 28 | 28 |

RPKM

| | A1 | A2 | A3 | A4 |
|---|---|---|---|---|
| GSVIVT01017522001 | 51 | 44 | 38 | 37 |
| GSVIVT01020266001 | 3 | 3 | 2 | 2 |
| GSVIVT01020269001 | 0 | 0 | 0 | 0 |

RPKM

| | A1 | A2 | A3 | A4 |
|---|---|---|---|---|
| GSVIVT01013167001 | 66 | 61 | 69 | 65 |

# Pathway: histidine biosynthesis

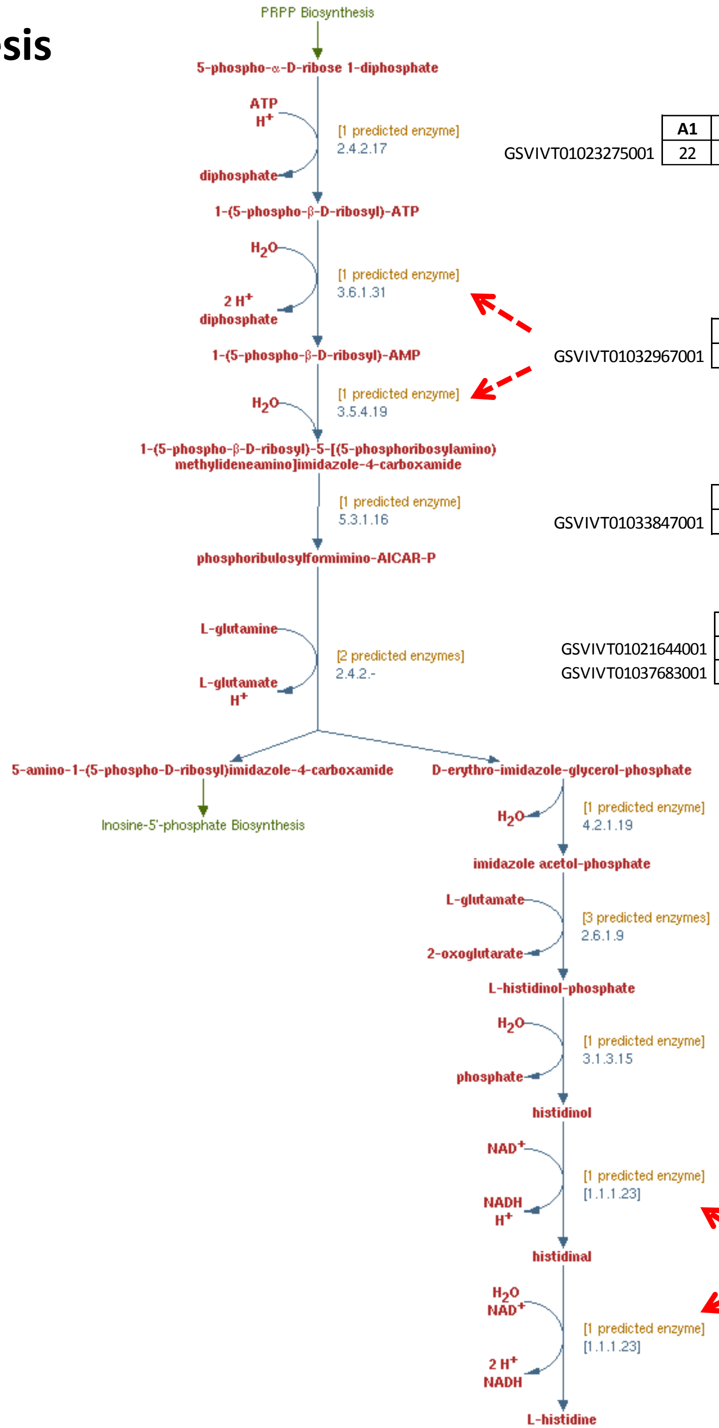


RPKM

| | A1 | A2 | A3 | A4 |
|---|---|---|---|---|
| GSVIVT01023275001 | 22 | 21 | 19 | 24 |

RPKM

| | A1 | A2 | A3 | A4 |
|---|---|---|---|---|
| GSVIVT01032967001 | 24 | 25 | 11 | 12 |

RPKM

| | A1 | A2 | A3 | A4 |
|---|---|---|---|---|
| GSVIVT01033847001 | 11 | 10 | 8 | 8 |

RPKM

| | A1 | A2 | A3 | A4 |
|---|---|---|---|---|
| GSVIVT01021644001 | 0 | 0 | 0 | 0 |
| GSVIVT01037683001 | 18 | 17 | 14 | 13 |

RPKM

| | A1 | A2 | A3 | A4 |
|---|---|---|---|---|
| GSVIVT01017973001 | 23 | 21 | 20 | 21 |

RPKM

| | A1 | A2 | A3 | A4 |
|---|---|---|---|---|
| GSVIVT01025260001 | 0 | 0 | 0 | 0 |
| GSVIVT01025261001 | 2 | 2 | 1 | 1 |
| GSVIVT01034496001 | 2 | 2 | 1 | 1 |

RPKM

| | A1 | A2 | A3 | A4 |
|---|---|---|---|---|
| GSVIVT01023826001 | 21 | 22 | 21 | 18 |

RPKM

| | A1 | A2 | A3 | A4 |
|---|---|---|---|---|
| GSVIVT01001692001 | 12 | 13 | 15 | 18 |

# Metabolic Pathway PWY-4983
# “citrulline-nitric oxide cycle”

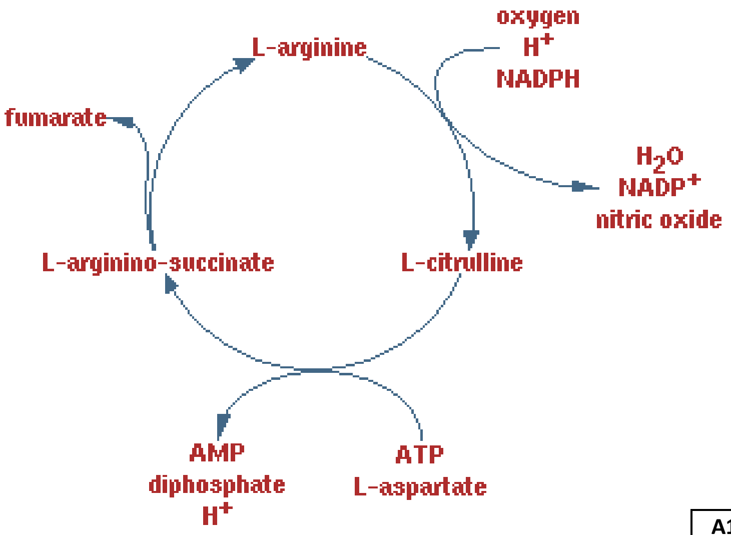


[1 predicted enzyme]
4.3.2.1

RPKM

| | A1 | A2 | A3 | A4 |
|---|---|---|---|---|
| GSVIVT01009181001 | 28 | 28 | 20 | 19 |

[4 predicted enzymes]
1.14.13.39

RPKM

| | A1 | A2 | A3 | A4 |
|---|---|---|---|---|
| GSVIVT01018681001 | 9 | 8 | 6 | 6 |
| GSVIVT01018708001 | 0 | 0 | 0 | 0 |
| GSVIVT01025382001 | 7 | 10 | 6 | 5 |
| GSVIVT01033559001 | 5 | 6 | 5 | 5 |

[2 predicted enzymes]
6.3.4.5

RPKM

| | A1 | A2 | A3 | A4 |
|---|---|---|---|---|
| GSVIVT01008510001 | 103 | 94 | 58 | 65 |
| GSVIVT01014064001 | Not Found in Sultanina | | | |

# Pathway: pentose phosphate pathway (oxidative branch) I

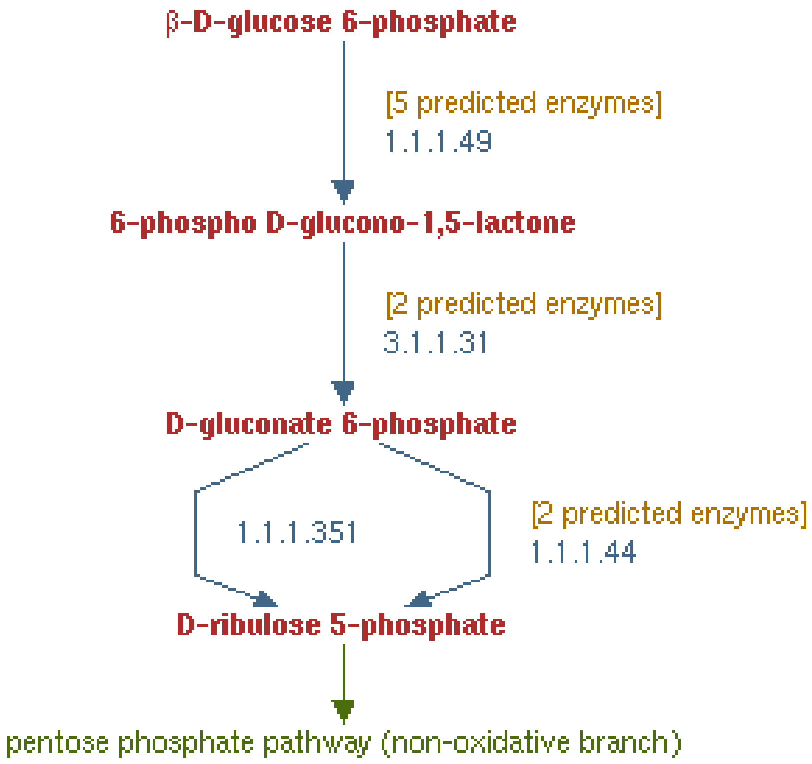


RPKM

| | A1 | A2 | A3 | A4 |
|---|---|---|---|---|
| GSVIVT01000913001 | 17 | 24 | 19 | 12 |
| GSVIVT01001847001 | 55 | 55 | 56 | 64 |
| GSVIVT01030079001 | 12 | 11 | 7 | 6 |
| GSVIVT01030086001 | 3 | 2 | 1 | 1 |
| GSVIVT01031295001 | 37 | 29 | 23 | 21 |

RPKM

| | A1 | A2 | A3 | A4 |
|---|---|---|---|---|
| GSVIVT01012236001 | 42 | 33 | 36 | 42 |
| GSVIVT01038616001 | 83 | 244 | 304 | 409 |

RPKM

| | A1 | A2 | A3 | A4 |
|---|---|---|---|---|
| GSVIVT01010170001 | 178 | 279 | 380 | 448 |
| GSVIVT01010171001 | 115 | 128 | 116 | 75 |

# Metabolic Pathway PWY-5723 "Rubisco Shunt"

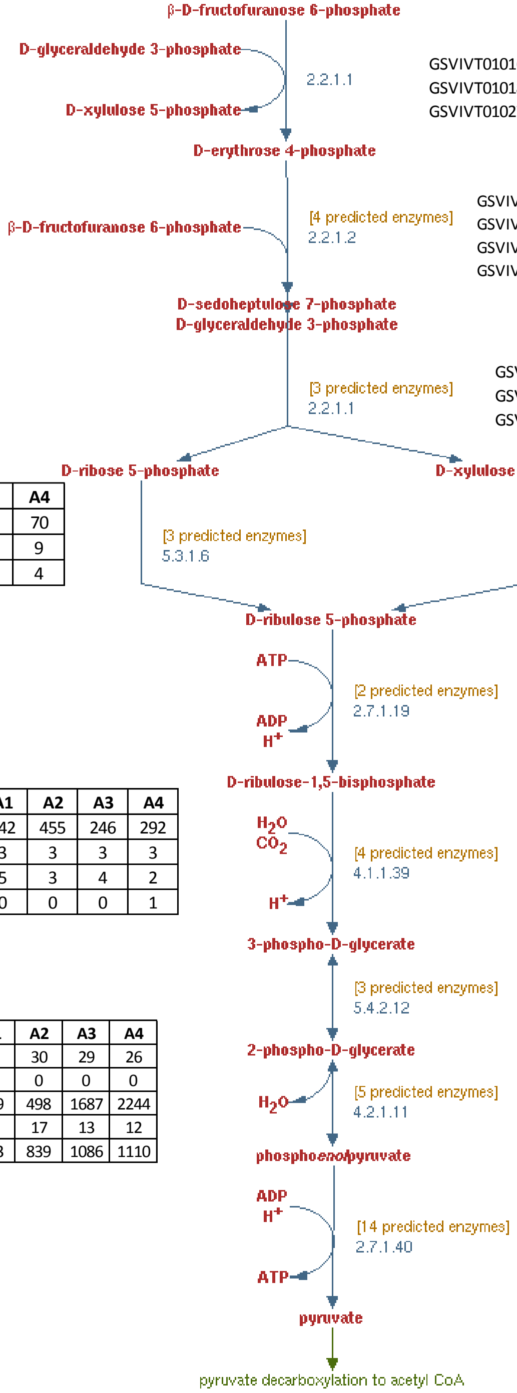


| | A1 | A2 | A3 | A4 |
|---|---|---|---|---|
| GSVIVT01016247001 | 3 | 3 | 3 | 3 |
| GSVIVT01018555001 | 133 | 242 | 525 | 500 |
| GSVIVT01027667001 | 263 | 31 | 4 | 1 |

| | A1 | A2 | A3 | A4 |
|---|---|---|---|---|
| GSVIVT01001098001 | 232 | 197 | 212 | 257 |
| GSVIVT01001274001 | 0 | 0 | 0 | 0 |
| GSVIVT01004774001 | 0 | 0 | 0 | 0 |
| GSVIVT01028518001 | 0 | 1 | 0 | 0 |

| | A1 | A2 | A3 | A4 |
|---|---|---|---|---|
| GSVIVT01016247001 | 3 | 3 | 3 | 3 |
| GSVIVT01018555001 | 133 | 242 | 525 | 500 |
| GSVIVT01027667001 | 263 | 31 | 4 | 1 |

| | A1 | A2 | A3 | A4 |
|---|---|---|---|---|
| GSVIVT01021828001 | 62 | 69 | 67 | 70 |
| GSVIVT01025127001 | 0 | 3 | 5 | 9 |
| GSVIVT01037941001 | 3 | 5 | 3 | 4 |

| | A1 | A2 | A3 | A4 |
|---|---|---|---|---|
| GSVIVT01001810001 | 42 | 31 | 21 | 18 |
| GSVIVT01007976001 | 64 | 58 | 29 | 31 |

| | A1 | A2 | A3 | A4 |
|---|---|---|---|---|
| GSVIVT01011555001 | 65 | 58 | 30 | 34 |
| GSVIVT01024410001 | 0 | 0 | 0 | 0 |

| | A1 | A2 | A3 | A4 |
|---|---|---|---|---|
| GSVIVT01008288001 | 542 | 455 | 246 | 292 |
| GSVIVT01013856001 | 3 | 3 | 3 | 3 |
| GSVIVT01022403001 | 5 | 3 | 4 | 2 |
| GSVIVT01029221001 | 0 | 0 | 0 | 1 |

| | A1 | A2 | A3 | A4 |
|---|---|---|---|---|
| GSVIVT01008609001 | 16 | 17 | 22 | 23 |
| GSVIVT01029842001 | 571 | 782 | 1265 | 1327 |
| GSVIVT01036480001 | 1 | 1 | 1 | 0 |

| | A1 | A2 | A3 | A4 |
|---|---|---|---|---|
| GSVIVT01008197001 | 21 | 30 | 29 | 26 |
| GSVIVT01015129001 | 0 | 0 | 0 | 0 |
| GSVIVT01018517001 | 199 | 498 | 1687 | 2244 |
| GSVIVT01024818001 | 23 | 17 | 13 | 12 |
| GSVIVT01033770001 | 773 | 839 | 1086 | 1110 |

| | A1 | A2 | A3 | A4 |
|---|---|---|---|---|
| GSVIVT01003140001 | 20 | 16 | 12 | 10 |
| GSVIVT01013170001 | 37 | 52 | 60 | 55 |
| GSVIVT01018079001 | 59 | 60 | 45 | 45 |
| GSVIVT01025504001 | 0 | 3 | 3 | 3 |
| GSVIVT01028701001 | 34 | 37 | 49 | 43 |
| GSVIVT01028756001 | 67 | 73 | 83 | 81 |
| GSVIVT01029840001 | 176 | 218 | 175 | 176 |
| GSVIVT01033379001 | 6 | 4 | 2 | 1 |
| GSVIVT01033599001 | 4 | 4 | 2 | 2 |
| GSVIVT01033605001 | 0 | 0 | 0 | 0 |
| GSVIVT01033747001 | 219 | 292 | 445 | 422 |
| GSVIVT01034344001 | 61 | 88 | 178 | 258 |
| GSVIVT01034684001 | 43 | 31 | 36 | 31 |
| GSVIVT01037043001 | 17 | 21 | 17 | 16 |

# Pathway: zeaxanthin biosynthesis

| | A1 | A2 | A3 | A4 |
|---|---|---|---|---|
| GSVIVT01019403001 | 0 | 1 | 2 | 1 |
| GSVIVT01028864001 | 35 | 28 | 9 | 5 |

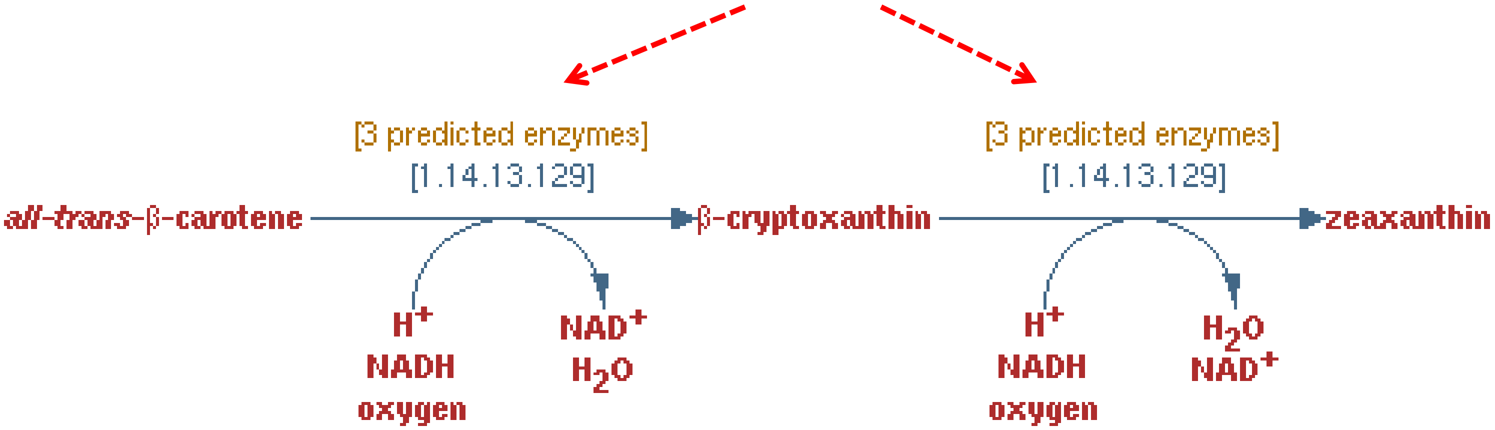

# Metabolic Pathway PWY-5944 “zeaxanthin biosynthesis”

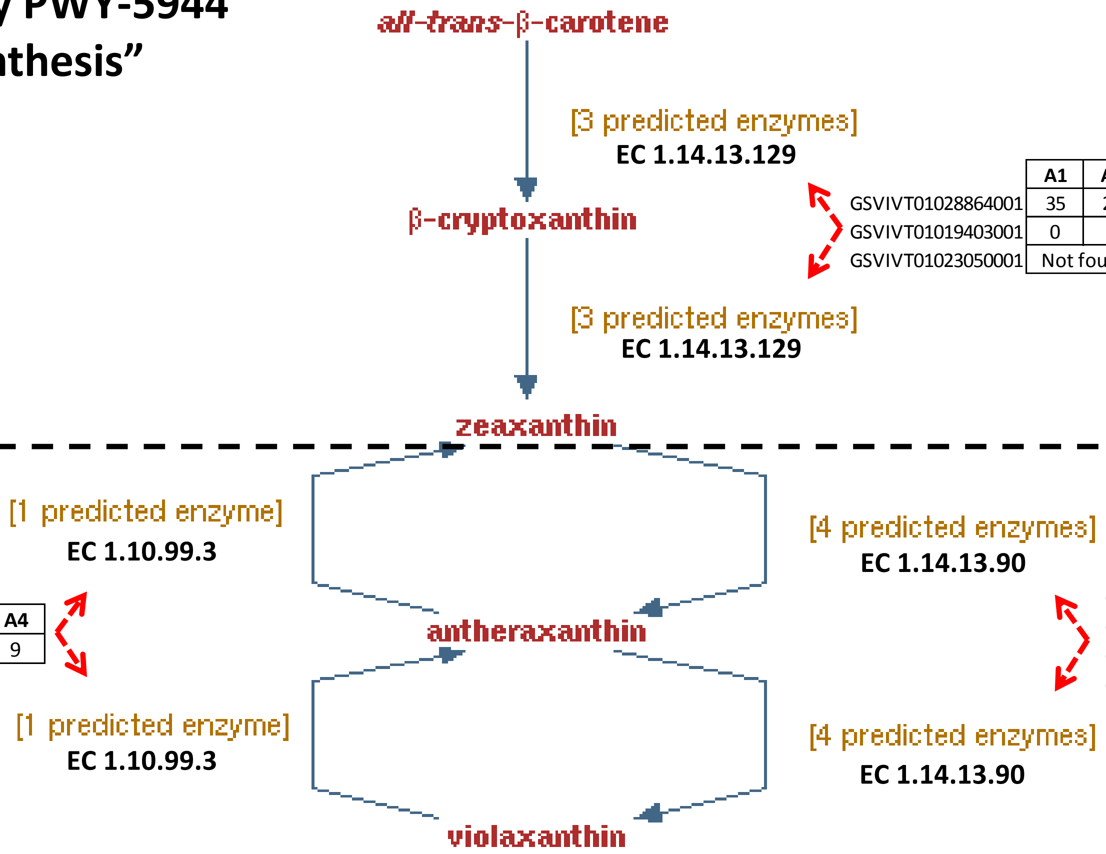


RPKM

| | A1 | A2 | A3 | A4 |
|---|---|---|---|---|
| GSVIVT01028864001 | 35 | 28 | 9 | 5 |
| GSVIVT01019403001 | 0 | 1 | 2 | 0 |
| GSVIVT01023050001 | Not found in Sultanina | | | |

RPKM

| | A1 | A2 | A3 | A4 |
|---|---|---|---|---|
| GSVIVT01026405001 | 7 | 8 | 7 | 9 |

RPKM

| | A1 | A2 | A3 | A4 |
|---|---|---|---|---|
| GSVIVT01001372001 | 12 | 11 | 9 | 9 |
| GSVIVT01001999001 | 19 | 11 | 12 | 8 |
| GSVIVT01003729001 | 800 | 792 | 528 | 630 |
| GSVIVT01022128001 | 34 | 19 | 9 | 9 |

# Metabolic Pathway PWY-5945 “zeaxanthin, antheraxanthin and violaxanthin interconversion”

# Metabolic Pathway PWY-922
# “mevalonate pathway I”

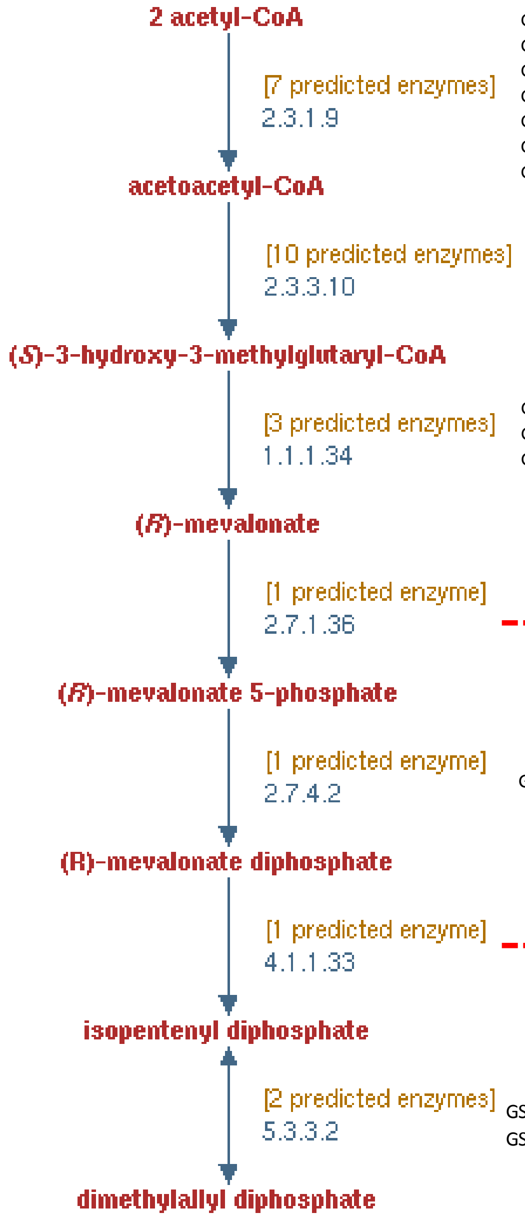


RPKM

| | A1 | A2 | A3 | A4 |
|---|---|---|---|---|
| GSVIVT01007332001 | 5 | 7 | 11 | 13 |
| GSVIVT01030112001 | 270 | 356 | 472 | 513 |
| GSVIVT01037163001 | 0 | 0 | 0 | 0 |
| GSVIVT01037164001 | 0 | 0 | 0 | 0 |
| GSVIVT01037168001 | 2 | 1 | 0 | 0 |
| GSVIVT01037169001 | 0 | 0 | 0 | 0 |
| GSVIVT01037175001 | 1 | 1 | 1 | 0 |

RPKM

| | A1 | A2 | A3 | A4 |
|---|---|---|---|---|
| GSVIVT01008388001 | 0 | 0 | 0 | 0 |
| GSVIVT01010658001 | 0 | 0 | 0 | 0 |
| GSVIVT01010662001 | 0 | 0 | 0 | 0 |
| GSVIVT01015848001 | 0 | 0 | 0 | 0 |
| GSVIVT01018434001 | 0 | 0 | 0 | 0 |
| GSVIVT01018566001 | 0 | 0 | 0 | 0 |
| GSVIVT01019875001 | 37 | 41 | 56 | 34 |
| GSVIVT01023489001 | 0 | 0 | 0 | 0 |
| GSVIVT01023491001 | 0 | 0 | 0 | 0 |

RPKM

| | A1 | A2 | A3 | A4 |
|---|---|---|---|---|
| GSVIVT01013435001 | 1 | 1 | 0 | 0 |
| GSVIVT01023852001 | 20 | 42 | 51 | 86 |
| GSVIVT01026444001 | 120 | 96 | 21 | 9 |

RPKM

| | A1 | A2 | A3 | A4 |
|---|---|---|---|---|
| GSVIVT01000037001 | 21 | 16 | 17 | 16 |

RPKM

| | A1 | A2 | A3 | A4 |
|---|---|---|---|---|
| GSVIVT01013024001 | 36 | 35 | 34 | 29 |

RPKM

| | A1 | A2 | A3 | A4 |
|---|---|---|---|---|
| GSVIVT01011287001 | 45 | 59 | 47 | 43 |

RPKM

| | A1 | A2 | A3 | A4 |
|---|---|---|---|---|
| GSVIVT01016092001 | 27 | 26 | 31 | 26 |
| GSVIVT01019089001 | 147 | 382 | 525 | 471 |

# Glycolysis I (from glucose 6-phosphate)

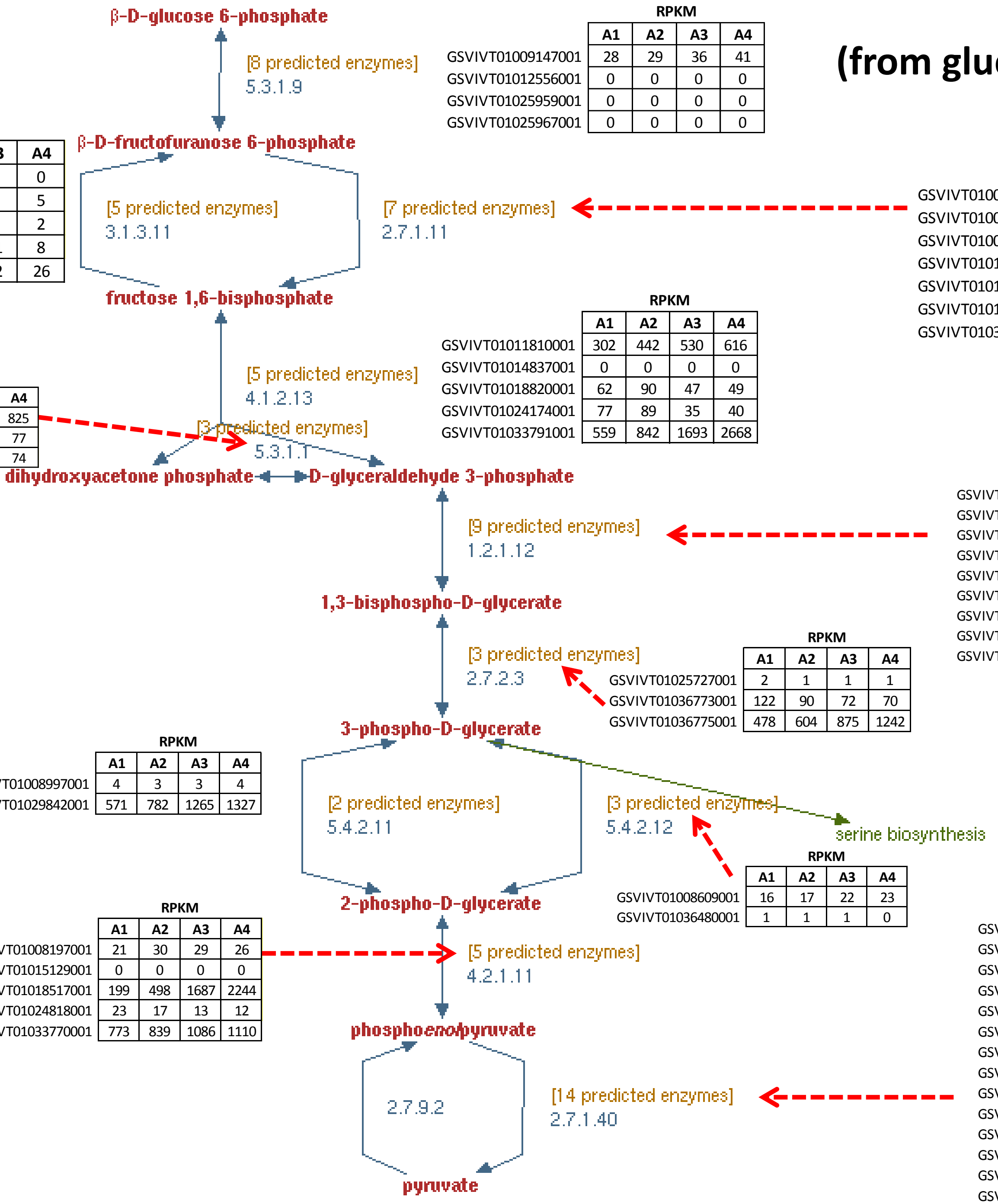


5.3.1.9 — RPKM

| | A1 | A2 | A3 | A4 |
|---|---|---|---|---|
| GSVIVT01009147001 | 28 | 29 | 36 | 41 |
| GSVIVT01012556001 | 0 | 0 | 0 | 0 |
| GSVIVT01025959001 | 0 | 0 | 0 | 0 |
| GSVIVT01025967001 | 0 | 0 | 0 | 0 |

3.1.3.11 — RPKM

| | A1 | A2 | A3 | A4 |
|---|---|---|---|---|
| GSVIVT01011038001 | 0 | 0 | 0 | 0 |
| GSVIVT01012513001 | 8 | 6 | 5 | 5 |
| GSVIVT01034034001 | 4 | 4 | 2 | 2 |
| GSVIVT01034037001 | 33 | 25 | 11 | 8 |
| GSVIVT01034516001 | 25 | 33 | 22 | 26 |

2.7.1.11 — RPKM

| | A1 | A2 | A3 | A4 |
|---|---|---|---|---|
| GSVIVT01002583001 | 0 | 0 | 0 | 0 |
| GSVIVT01003611001 | 1 | 1 | 0 | 0 |
| GSVIVT01003613001 | 1 | 1 | 1 | 0 |
| GSVIVT01011393001 | 3 | 2 | 3 | 3 |
| GSVIVT01013938001 | 16 | 18 | 29 | 30 |
| GSVIVT01015271001 | 59 | 82 | 85 | 83 |
| GSVIVT01035322001 | 56 | 144 | 189 | 225 |

4.1.2.13 — RPKM

| | A1 | A2 | A3 | A4 |
|---|---|---|---|---|
| GSVIVT01011810001 | 302 | 442 | 530 | 616 |
| GSVIVT01014837001 | 0 | 0 | 0 | 0 |
| GSVIVT01018820001 | 62 | 90 | 47 | 49 |
| GSVIVT01024174001 | 77 | 89 | 35 | 40 |
| GSVIVT01033791001 | 559 | 842 | 1693 | 2668 |

5.3.1.1 — RPKM

| | A1 | A2 | A3 | A4 |
|---|---|---|---|---|
| GSVIVT01016559001 | 382 | 469 | 687 | 825 |
| GSVIVT01024086001 | 75 | 71 | 73 | 77 |
| GSVIVT01024952001 | 94 | 79 | 76 | 74 |

1.2.1.12 — RPKM

| | A1 | A2 | A3 | A4 |
|---|---|---|---|---|
| GSVIVT01001842001 | 0 | 0 | 0 | 0 |
| GSVIVT01007521001 | 2181 | 2412 | 2972 | 3352 |
| GSVIVT01010328001 | 96 | 496 | 1068 | 1343 |
| GSVIVT01013403001 | 76 | 67 | 48 | 32 |
| GSVIVT01032942001 | 176 | 131 | 55 | 54 |
| GSVIVT01036751001 | 4 | 6 | 9 | 9 |
| GSVIVT01037165001 | 1 | 1 | 1 | 0 |
| GSVIVT01037170001 | 0 | 0 | 0 | 0 |
| GSVIVT01037176001 | 2 | 2 | 3 | 2 |

2.7.2.3 — RPKM

| | A1 | A2 | A3 | A4 |
|---|---|---|---|---|
| GSVIVT01025727001 | 2 | 1 | 1 | 1 |
| GSVIVT01036773001 | 122 | 90 | 72 | 70 |
| GSVIVT01036775001 | 478 | 604 | 875 | 1242 |

5.4.2.11 — RPKM

| | A1 | A2 | A3 | A4 |
|---|---|---|---|---|
| GSVIVT01008997001 | 4 | 3 | 3 | 4 |
| GSVIVT01029842001 | 571 | 782 | 1265 | 1327 |

5.4.2.12 — RPKM

| | A1 | A2 | A3 | A4 |
|---|---|---|---|---|
| GSVIVT01008609001 | 16 | 17 | 22 | 23 |
| GSVIVT01036480001 | 1 | 1 | 1 | 0 |

4.2.1.11 — RPKM

| | A1 | A2 | A3 | A4 |
|---|---|---|---|---|
| GSVIVT01008197001 | 21 | 30 | 29 | 26 |
| GSVIVT01015129001 | 0 | 0 | 0 | 0 |
| GSVIVT01018517001 | 199 | 498 | 1687 | 2244 |
| GSVIVT01024818001 | 23 | 17 | 13 | 12 |
| GSVIVT01033770001 | 773 | 839 | 1086 | 1110 |

2.7.1.40 — RPKM

| | A1 | A2 | A3 | A4 |
|---|---|---|---|---|
| GSVIVT01003140001 | 20 | 16 | 12 | 10 |
| GSVIVT01013170001 | 37 | 52 | 60 | 55 |
| GSVIVT01018079001 | 59 | 60 | 45 | 45 |
| GSVIVT01025504001 | 0 | 3 | 3 | 3 |
| GSVIVT01028701001 | 34 | 37 | 49 | 43 |
| GSVIVT01028756001 | 67 | 73 | 83 | 81 |
| GSVIVT01029840001 | 176 | 218 | 175 | 176 |
| GSVIVT01033379001 | 6 | 4 | 2 | 1 |
| GSVIVT01033599001 | 4 | 4 | 2 | 2 |
| GSVIVT01033605001 | 0 | 0 | 0 | 0 |
| GSVIVT01033747001 | 219 | 292 | 445 | 422 |
| GSVIVT01034344001 | 61 | 88 | 178 | 258 |
| GSVIVT01034684001 | 43 | 31 | 36 | 31 |
| GSVIVT01037043001 | 17 | 21 | 17 | 16 |
