# Supplementary material for: Transcriptome analysis during ripening of table grape berry cv. Thompson Seedless
Source: PLoS One. 2018 Jan 10;13(1):e0190087. doi: 10.1371/journal.pone.0190087 (PMC5761854; doi:10.1371/journal.pone.0190087)
Supplement: S2 Fig — (PDF) [file pone.0190087.s002.pdf]

Glycolysis I
(from glucose 6-phosphate)

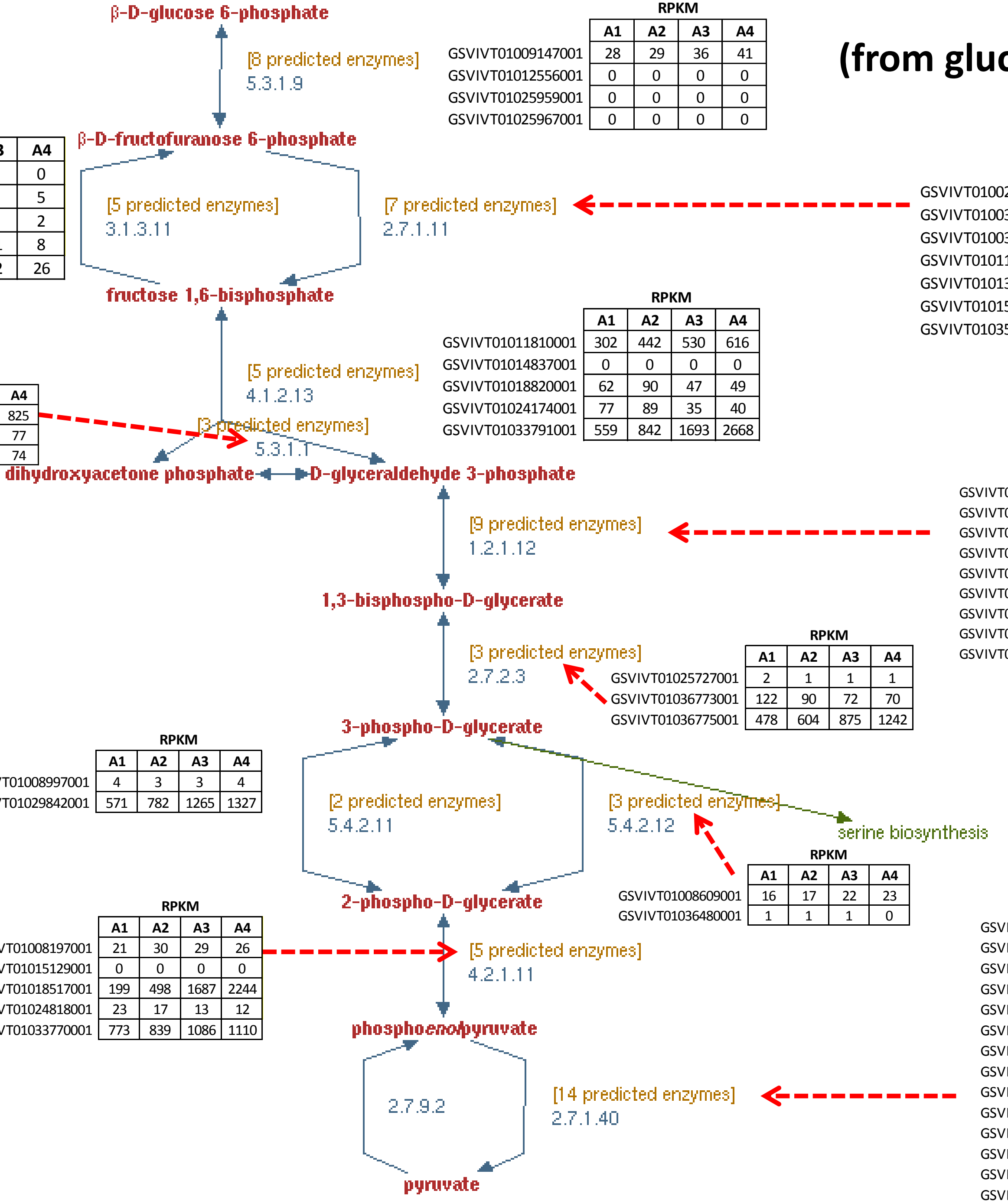

RPKM (5.3.1.9)

	A1	A2	A3	A4
GSVIVT01009147001	28	29	36	41
GSVIVT01012556001	0	0	0	0
GSVIVT01025959001	0	0	0	0
GSVIVT01025967001	0	0	0	0

RPKM (3.1.3.11)

	A1	A2	A3	A4
GSVIVT01011038001	0	0	0	0
GSVIVT01012513001	8	6	5	5
GSVIVT01034034001	4	4	2	2
GSVIVT01034037001	33	25	11	8
GSVIVT01034516001	25	33	22	26

RPKM (2.7.1.11)

	A1	A2	A3	A4
GSVIVT01002583001	0	0	0	0
GSVIVT01003611001	1	1	0	0
GSVIVT01003613001	1	1	1	0
GSVIVT01011393001	3	2	3	3
GSVIVT01013938001	16	18	29	30
GSVIVT01015271001	59	82	85	83
GSVIVT01035322001	56	144	189	225

RPKM (4.1.2.13)

	A1	A2	A3	A4
GSVIVT01011810001	302	442	530	616
GSVIVT01014837001	0	0	0	0
GSVIVT01018820001	62	90	47	49
GSVIVT01024174001	77	89	35	40
GSVIVT01033791001	559	842	1693	2668

RPKM (5.3.1.1)

	A1	A2	A3	A4
GSVIVT01016559001	382	469	687	825
GSVIVT01024086001	75	71	73	77
GSVIVT01024952001	94	79	76	74

RPKM (1.2.1.12)

	A1	A2	A3	A4
GSVIVT01001842001	0	0	0	0
GSVIVT01007521001	2181	2412	2972	3352
GSVIVT01010328001	96	496	1068	1343
GSVIVT01013403001	76	67	48	32
GSVIVT01032942001	176	131	55	54
GSVIVT01036751001	4	6	9	9
GSVIVT01037165001	1	1	1	0
GSVIVT01037170001	0	0	0	0
GSVIVT01037176001	2	2	3	2

RPKM (2.7.2.3)

	A1	A2	A3	A4
GSVIVT01025727001	2	1	1	1
GSVIVT01036773001	122	90	72	70
GSVIVT01036775001	478	604	875	1242

RPKM (5.4.2.11)

	A1	A2	A3	A4
GSVIVT01008997001	4	3	3	4
GSVIVT01029842001	571	782	1265	1327

RPKM (5.4.2.12)

	A1	A2	A3	A4
GSVIVT01008609001	16	17	22	23
GSVIVT01036480001	1	1	1	0

RPKM (4.2.1.11)

	A1	A2	A3	A4
GSVIVT01008197001	21	30	29	26
GSVIVT01015129001	0	0	0	0
GSVIVT01018517001	199	498	1687	2244
GSVIVT01024818001	23	17	13	12
GSVIVT01033770001	773	839	1086	1110

RPKM (2.7.1.40)

	A1	A2	A3	A4
GSVIVT01003140001	20	16	12	10
GSVIVT01013170001	37	52	60	55
GSVIVT01018079001	59	60	45	45
GSVIVT01025504001	0	3	3	3
GSVIVT01028701001	34	37	49	43
GSVIVT01028756001	67	73	83	81
GSVIVT01029840001	176	218	175	176
GSVIVT01033379001	6	4	2	1
GSVIVT01033599001	4	4	2	2
GSVIVT01033605001	0	0	0	0
GSVIVT01033747001	219	292	445	422
GSVIVT01034344001	61	88	178	258
GSVIVT01034684001	43	31	36	31
GSVIVT01037043001	17	21	17	16

Glycolysis IV (plant cytosol)

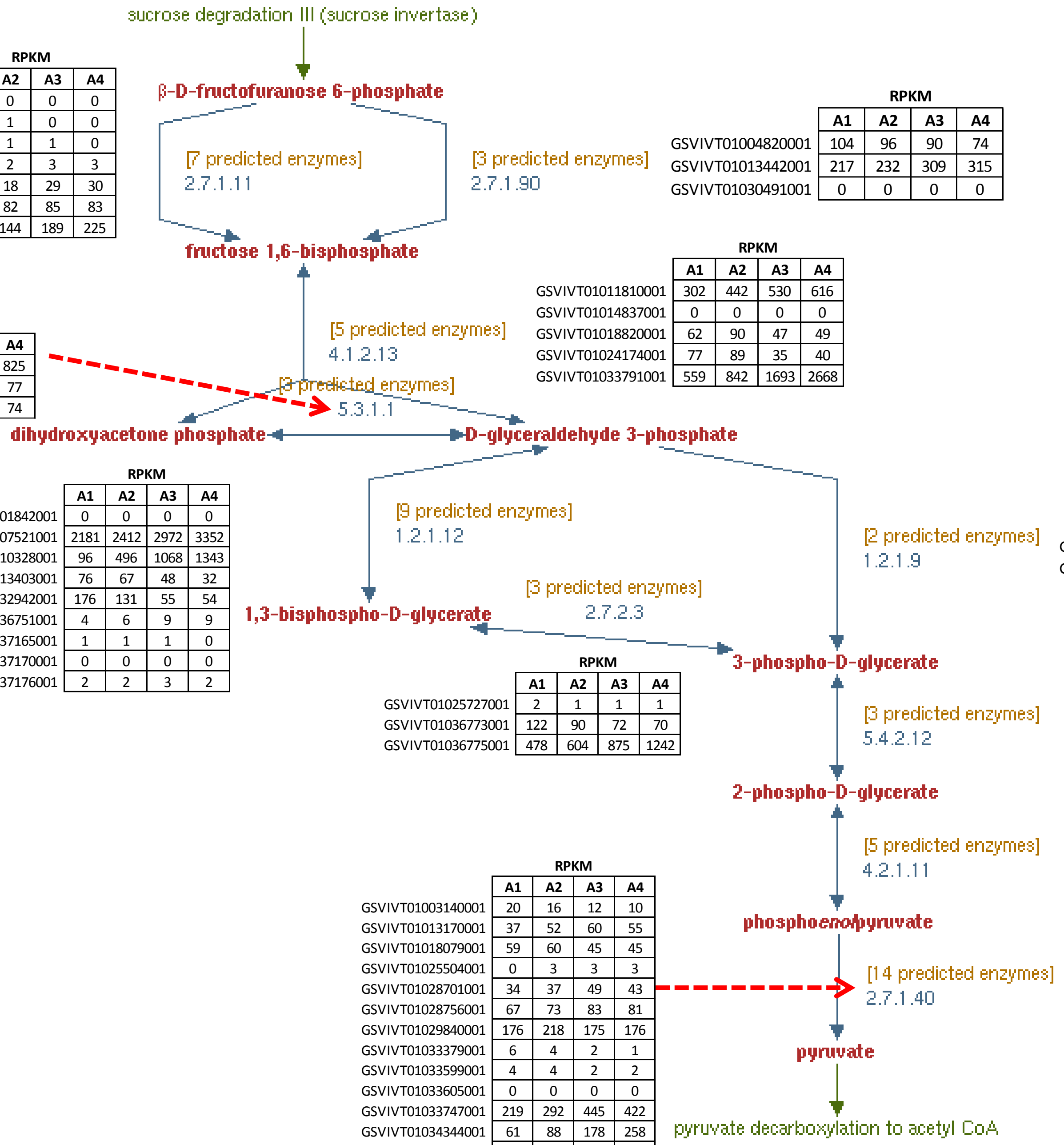

RPKM

	A1	A2	A3	A4
GSVIVT01002583001	0	0	0	0
GSVIVT01003611001	1	1	0	0
GSVIVT01003613001	1	1	1	0
GSVIVT01011393001	3	2	3	3
GSVIVT01013938001	16	18	29	30
GSVIVT01015271001	59	82	85	83
GSVIVT01035322001	56	144	189	225

RPKM

	A1	A2	A3	A4
GSVIVT01004820001	104	96	90	74
GSVIVT01013442001	217	232	309	315
GSVIVT01030491001	0	0	0	0

RPKM

	A1	A2	A3	A4
GSVIVT01011810001	302	442	530	616
GSVIVT01014837001	0	0	0	0
GSVIVT01018820001	62	90	47	49
GSVIVT01024174001	77	89	35	40
GSVIVT01033791001	559	842	1693	2668

RPKM

	A1	A2	A3	A4
GSVIVT01016559001	382	469	687	825
GSVIVT01024086001	75	71	73	77
GSVIVT01024952001	94	79	76	74

RPKM

	A1	A2	A3	A4
GSVIVT01001842001	0	0	0	0
GSVIVT01007521001	2181	2412	2972	3352
GSVIVT01010328001	96	496	1068	1343
GSVIVT01013403001	76	67	48	32
GSVIVT01032942001	176	131	55	54
GSVIVT01036751001	4	6	9	9
GSVIVT01037165001	1	1	1	0
GSVIVT01037170001	0	0	0	0
GSVIVT01037176001	2	2	3	2

RPKM

	A1	A2	A3	A4
GSVIVT01023590001	98	127	64	56
GSVIVT01035891001	45	40	44	59

RPKM

	A1	A2	A3	A4
GSVIVT01025727001	2	1	1	1
GSVIVT01036773001	122	90	72	70
GSVIVT01036775001	478	604	875	1242

RPKM

	A1	A2	A3	A4
GSVIVT01008609001	16	17	22	23
GSVIVT01029842001	571	782	1265	1327
GSVIVT01036480001	1	1	1	0

RPKM

	A1	A2	A3	A4
GSVIVT01008197001	21	30	29	26
GSVIVT01015129001	0	0	0	0
GSVIVT01018517001	199	498	1687	2244
GSVIVT01024818001	23	17	13	12
GSVIVT01033770001	773	839	1086	1110

RPKM

	A1	A2	A3	A4
GSVIVT01003140001	20	16	12	10
GSVIVT01013170001	37	52	60	55
GSVIVT01018079001	59	60	45	45
GSVIVT01025504001	0	3	3	3
GSVIVT01028701001	34	37	49	43
GSVIVT01028756001	67	73	83	81
GSVIVT01029840001	176	218	175	176
GSVIVT01033379001	6	4	2	1
GSVIVT01033599001	4	4	2	2
GSVIVT01033605001	0	0	0	0
GSVIVT01033747001	219	292	445	422
GSVIVT01034344001	61	88	178	258
GSVIVT01034684001	43	31	36	31
GSVIVT01037043001	17	21	17	16

Pathway: TCA cycle II (plants and fungi)

RPKM

	A1	A2	A3	A4
GSVIVT01006474001	53	39	60	62
GSVIVT01007997001	45	57	49	58
GSVIVT01010395001	2	3	2	3
GSVIVT01011422001	25	31	25	26
GSVIVT01014231001	31	24	45	54
GSVIVT01018190001	7	10	15	21
GSVIVT01021185001	160	186	270	344
GSVIVT01028330001	0	0	0	0
GSVIVT01028332001	0	0	0	0
GSVIVT01036965001	20	18	11	16

RPKM

	A1	A2	A3	A4
GSVIVT01000817001	38	45	55	51
GSVIVT01001358001	71	77	98	98

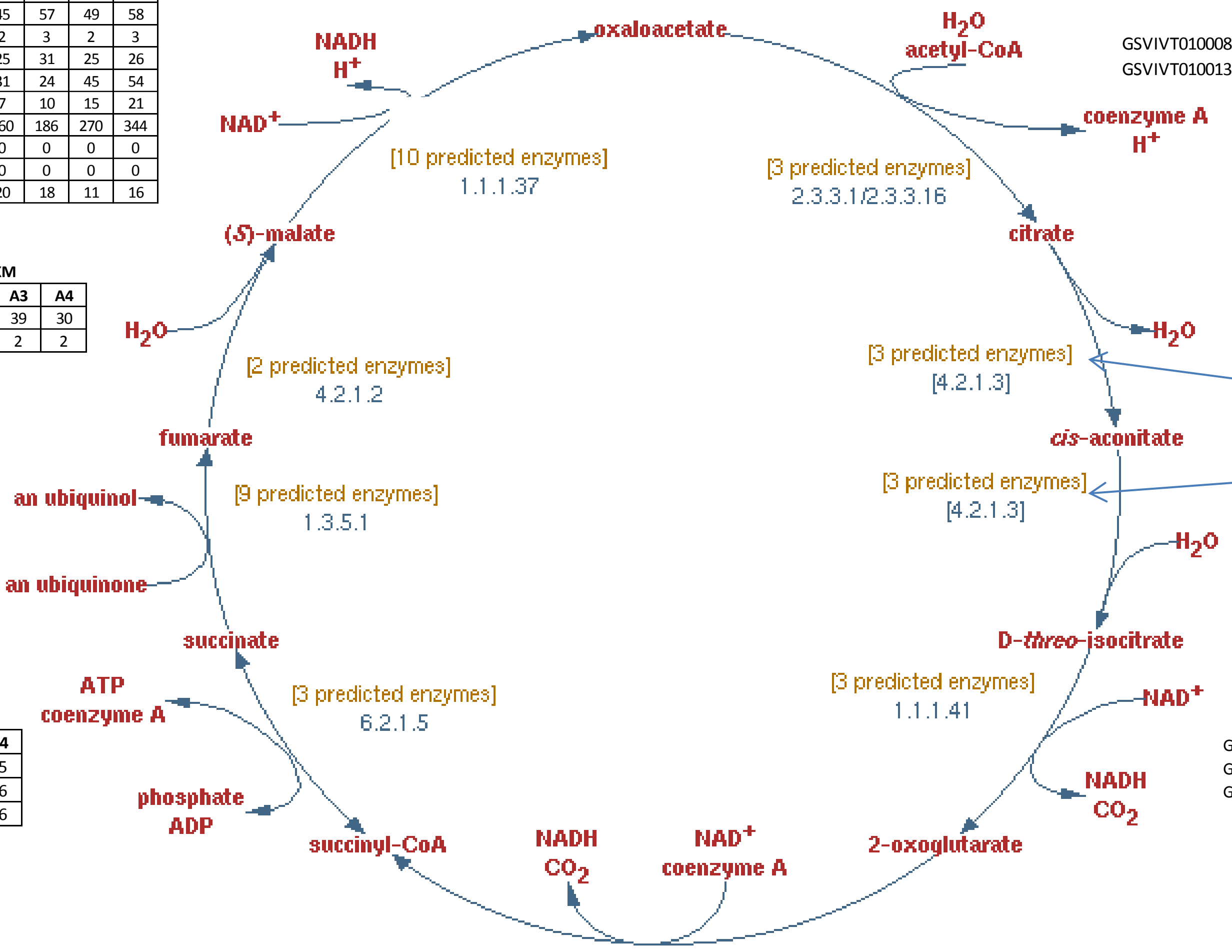

RPKM

	A1	A2	A3	A4
GSVIVT01028056001	46	43	39	30
GSVIVT01031185001	3	1	2	2

RPKM

	A1	A2	A3	A4
GSVIVT01005190001	122	108	100	81
GSVIVT01030588001	13	10	5	3
GSVIVT01037657001	90	94	138	122

RPKM

	A1	A2	A3	A4
GSVIVT01007813001	35	38	57	75
GSVIVT01009527001	66	66	76	95
GSVIVT01018976001	70	69	75	69
GSVIVT01027061001	79	80	84	118
GSVIVT01028464001	242	310	381	473

RPKM

	A1	A2	A3	A4
GSVIVT01026227001	19	20	17	15
GSVIVT01028373001	60	63	88	86
GSVIVT01029337001	42	50	58	66

RPKM

	A1	A2	A3	A4
GSVIVT01003837001	27	24	32	44
GSVIVT01023949001	44	57	63	65
GSVIVT01025704001	72	79	96	90

Metabolic Pathway PWY-5723 "Rubisco Shunt"

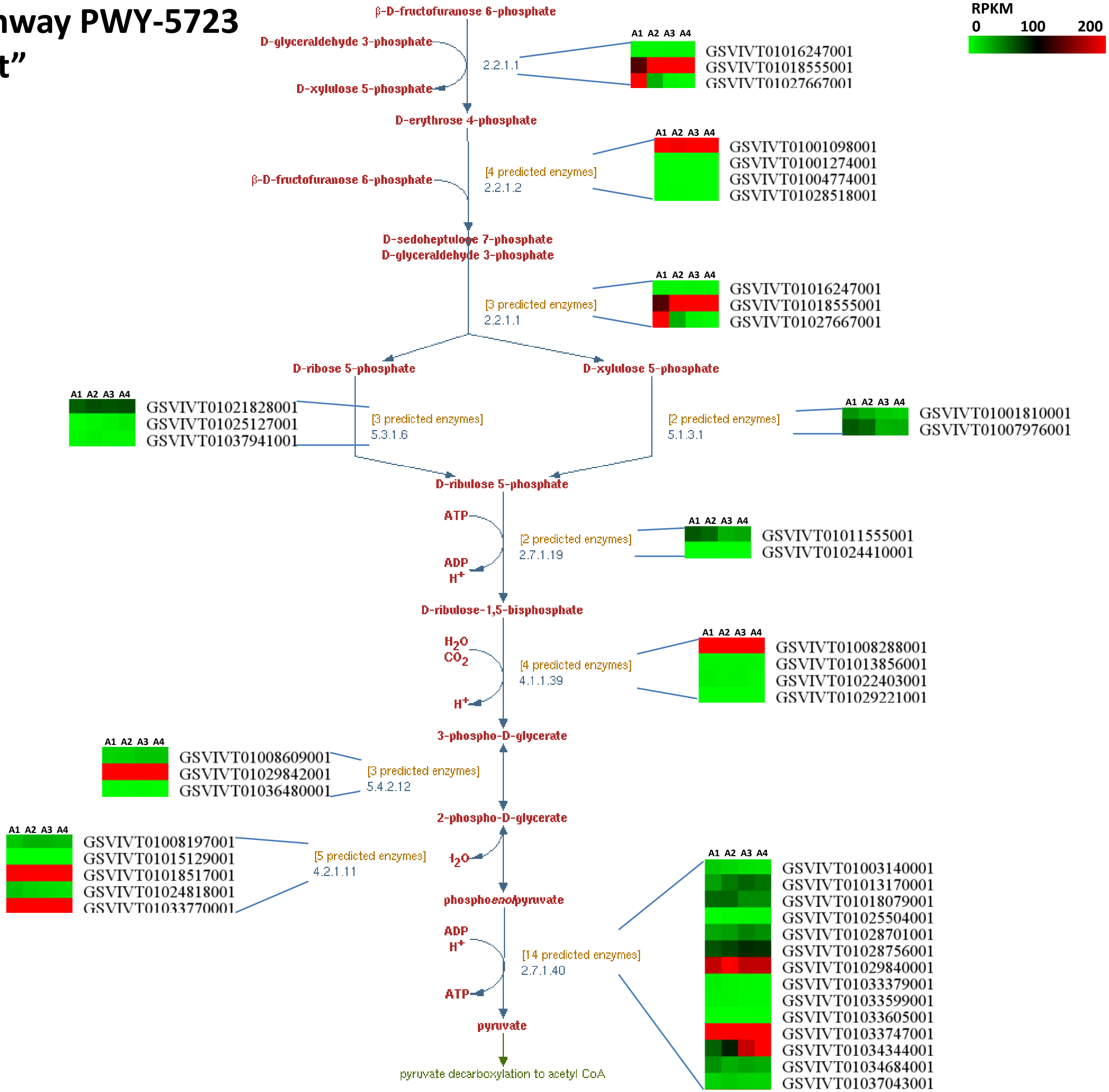

Pathway: aerobic respiration III (alternative oxidase pathway)

RPKM

	A1	A2	A3	A4
GSVIVT01000609001	63	78	94	135
GSVIVT01000718001	111	114	123	139
GSVIVT01002385001	0	0	0	0
GSVIVT01002443001	0	0	0	0
GSVIVT01004403001	0	0	0	0
GSVIVT01004958001	0	0	0	0
GSVIVT01004963001	0	0	0	0
GSVIVT01004966001	4	3	4	7
GSVIVT01004967001	1	0	1	1
GSVIVT01004976001	1	0	0	1
GSVIVT01006222001	0	0	0	0
GSVIVT01007203001	1	2	3	7
GSVIVT01008282001	542	648	1001	1390
GSVIVT01009735001	215	233	270	318
GSVIVT01010076001	30	45	57	110
GSVIVT01010482001	70	107	137	341
GSVIVT01010679001	103	86	106	171
GSVIVT01010904001	107	119	189	224
GSVIVT01011707001	8	7	2	1
GSVIVT01012613001	0	0	0	0
GSVIVT01013142001	3	2	5	6
GSVIVT01013345001	0	0	0	0

RPKM

	A1	A2	A3	A4
GSVIVT01013481001	0	0	0	0
GSVIVT01018929001	0	0	0	0
GSVIVT01020401001	129	164	227	293
GSVIVT01023768001	0	0	0	0
GSVIVT01024414001	63	59	72	94
GSVIVT01028438001	83	87	86	70
GSVIVT01028486001	85	90	112	125
GSVIVT01028820001	20	21	27	34
GSVIVT01029664001	1	1	1	1
GSVIVT01029731001	51	45	70	63
GSVIVT01029923001	0	0	0	0
GSVIVT01030914001	98	118	167	155
GSVIVT01031966001	96	111	142	177
GSVIVT01033348001	16	20	30	44
GSVIVT01035146001	48	63	81	96
GSVIVT01035355001	43	57	130	186
GSVIVT01036114001	165	242	528	712
GSVIVT01036643001	138	155	342	422
GSVIVT01036674001	0	0	0	0
GSVIVT01036676001	0	0	0	0
GSVIVT01037779001	59	82	97	133

RPKM

	A1	A2	A3	A4
GSVIVT01006689001	6	6	8	8
GSVIVT01007272001	3	3	7	8
GSVIVT01022812001	1	4	1	5
GSVIVT01022814001	10	17	20	41
GSVIVT01038745001	35	34	58	85

RPKM

	A1	A2	A3	A4
GSVIVT01007813001	35	38	57	75
GSVIVT01009527001	66	66	76	95
GSVIVT01018976001	70	69	75	69
GSVIVT01027061001	79	80	84	118
GSVIVT01028464001	242	310	381	473

[46 predicted enzymes]
1.6.5.3

[6 predicted enzymes]
1.10.3.11

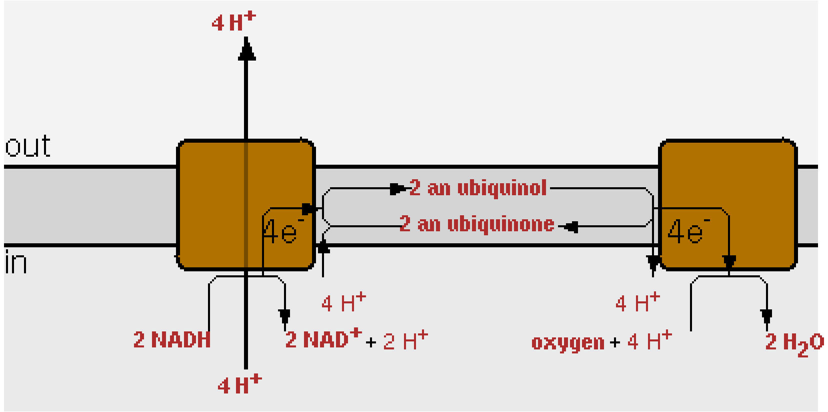

[9 predicted enzymes]
1.3.5.1

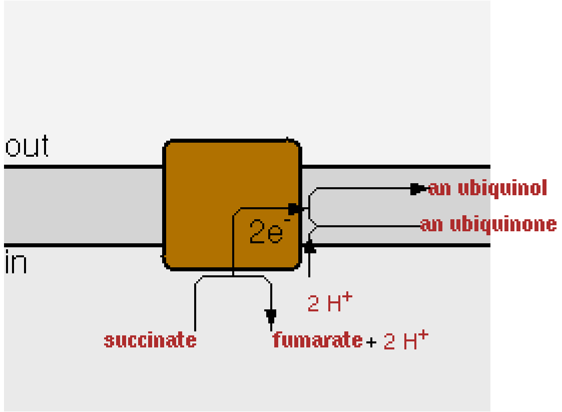

Pathway: aspartate degradation I

	RPKM			
	A1	A2	A3	A4
GSVIVT01008982001	7	10	9	10
GSVIVT01015252001	0	0	0	0
GSVIVT01015367001	92	109	165	198
GSVIVT01018494001	9	10	7	6
GSVIVT01019920001	4	3	2	1
GSVIVT01020739001	40	43	57	49
GSVIVT01020940001	0	0	0	0
GSVIVT01022161001	3	3	3	4
GSVIVT01022162001	4	2	1	1
GSVIVT01022163001	7	4	3	6
GSVIVT01030218001	8	8	26	34
GSVIVT01035662001	15	22	36	41
GSVIVT01035914001	124	121	116	119

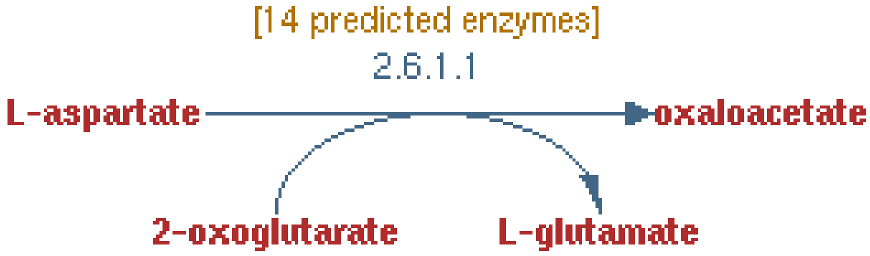

Pathway: aspartate biosynthesis

RPKM

	A1	A2	A3	A4
GSVIVT01008982001	7	10	9	10
GSVIVT01015252001	0	0	0	0
GSVIVT01015367001	92	109	165	198
GSVIVT01018494001	9	10	7	6
GSVIVT01019920001	4	3	2	1
GSVIVT01020739001	40	43	57	49
GSVIVT01020940001	0	0	0	0
GSVIVT01022161001	3	3	3	4
GSVIVT01022162001	4	2	1	1
GSVIVT01022163001	7	4	3	6
GSVIVT01030218001	8	8	26	34
GSVIVT01035662001	15	22	36	41
GSVIVT01035914001	124	121	116	119

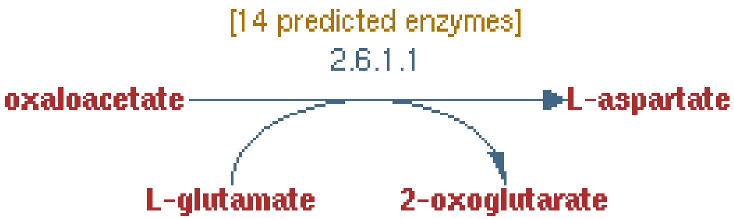

Pathway: uracil degradation I (reductive)

	RPKM			
	A1	A2	A3	A4
GSVIVT01008478001	81	137	253	291

	RPKM			
	A1	A2	A3	A4
GSVIVT01037327001	105	120	159	201

	RPKM			
	A1	A2	A3	A4
GSVIVT01015663001	33	48	63	77

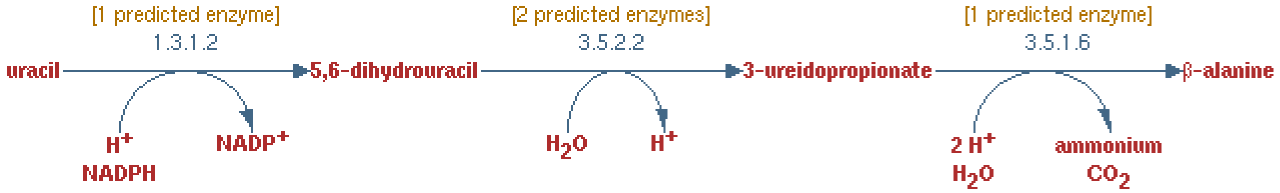

Pathway: gluconeogenesis I

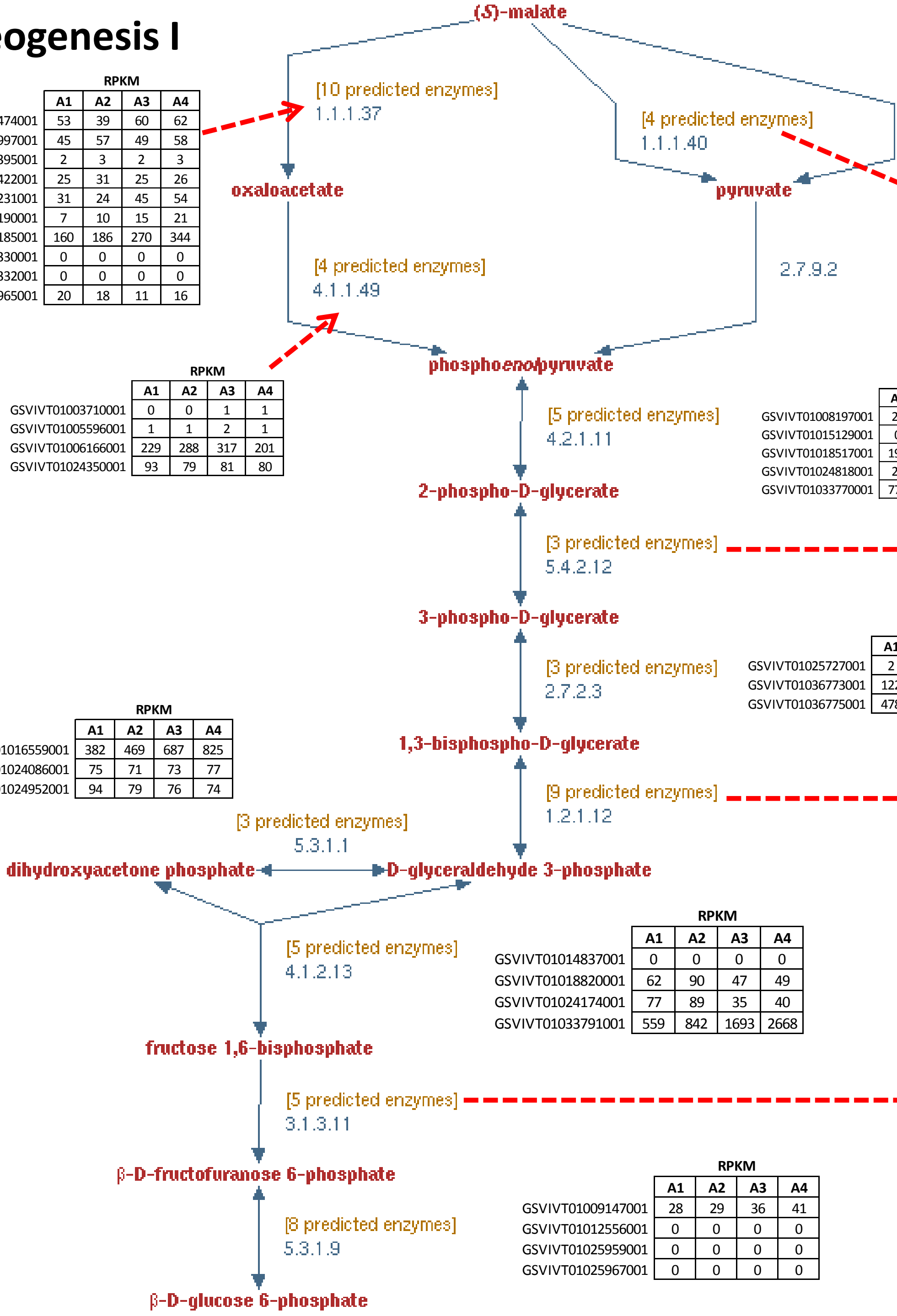

RPKM (1.1.1.37)

	A1	A2	A3	A4
GSVIVT01006474001	53	39	60	62
GSVIVT01007997001	45	57	49	58
GSVIVT01010395001	2	3	2	3
GSVIVT01011422001	25	31	25	26
GSVIVT01014231001	31	24	45	54
GSVIVT01018190001	7	10	15	21
GSVIVT01021185001	160	186	270	344
GSVIVT01028330001	0	0	0	0
GSVIVT01028332001	0	0	0	0
GSVIVT01036965001	20	18	11	16

RPKM (1.1.1.38/1.1.1.39)

	A1	A2	A3	A4
GSVIVT01018377001	90	69	74	64
GSVIVT01026824001	52	43	33	24

RPKM (1.1.1.40)

	A1	A2	A3	A4
GSVIVT01013039001	0	0	0	0
GSVIVT01015311001	314	417	452	548
GSVIVT01016997001	122	158	140	131
GSVIVT01035291001	1	1	6	3

RPKM (4.1.1.49)

	A1	A2	A3	A4
GSVIVT01003710001	0	0	1	1
GSVIVT01005596001	1	1	2	1
GSVIVT01006166001	229	288	317	201
GSVIVT01024350001	93	79	81	80

RPKM (4.2.1.11)

	A1	A2	A3	A4
GSVIVT01008197001	21	30	29	26
GSVIVT01015129001	0	0	0	0
GSVIVT01018517001	199	498	1687	2244
GSVIVT01024818001	23	17	13	12
GSVIVT01033770001	773	839	1086	1110

RPKM (5.4.2.12)

	A1	A2	A3	A4
GSVIVT01008609001	16	17	22	23
GSVIVT01029842001	571	782	1265	1327
GSVIVT01036480001	1	1	1	0

RPKM (2.7.2.3)

	A1	A2	A3	A4
GSVIVT01025727001	2	1	1	1
GSVIVT01036773001	122	90	72	70
GSVIVT01036775001	478	604	875	1242

RPKM (5.3.1.1)

	A1	A2	A3	A4
GSVIVT01016559001	382	469	687	825
GSVIVT01024086001	75	71	73	77
GSVIVT01024952001	94	79	76	74

RPKM (1.2.1.12)

	A1	A2	A3	A4
GSVIVT01001842001	0	0	0	0
GSVIVT01007521001	2181	2412	2972	3352
GSVIVT01010328001	96	496	1068	1343
GSVIVT01013403001	76	67	48	32
GSVIVT01032942001	176	131	55	54
GSVIVT01036751001	4	6	9	9
GSVIVT01037165001	1	1	1	0
GSVIVT01037170001	0	0	0	0
GSVIVT01037176001	2	2	3	2

RPKM (4.1.2.13)

	A1	A2	A3	A4
GSVIVT01014837001	0	0	0	0
GSVIVT01018820001	62	90	47	49
GSVIVT01024174001	77	89	35	40
GSVIVT01033791001	559	842	1693	2668

RPKM (3.1.3.11)

	A1	A2	A3	A4
GSVIVT01011810001	302	442	530	616
GSVIVT01014837001	0	0	0	0
GSVIVT01018820001	62	90	47	49
GSVIVT01024174001	77	89	35	40
GSVIVT01033791001	559	842	1693	2668

RPKM (5.3.1.9)

	A1	A2	A3	A4
GSVIVT01009147001	28	29	36	41
GSVIVT01012556001	0	0	0	0
GSVIVT01025959001	0	0	0	0
GSVIVT01025967001	0	0	0	0

Pathway: galactose degradation III

RPKM

	A1	A2	A3	A4
GSVIVT01034964001	85	96	139	200

RPKM

	A1	A2	A3	A4
GSVIVT01008799001	124	157	266	215

RPKM

	A1	A2	A3	A4
GSVIVT01019545001	1	3	7	8
GSVIVT01019547001	82	105	92	64
GSVIVT01019833001	21	29	16	13
GSVIVT01024169001	6	5	3	2

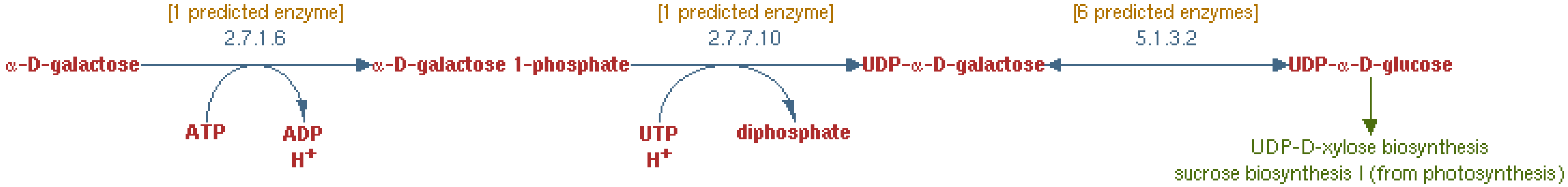

Pathway: 1D-myo-inositol hexakisphosphate biosynthesis III

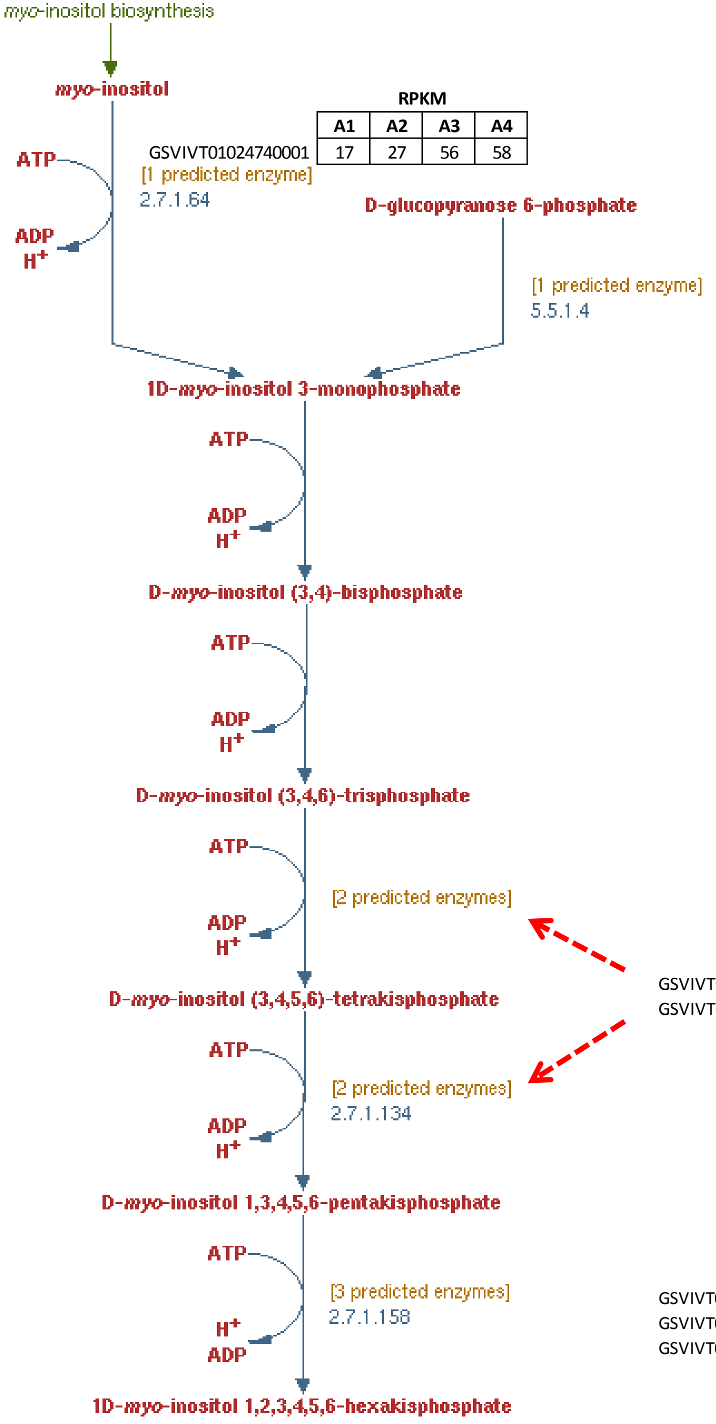

RPKM

	A1	A2	A3	A4
GSVIVT01024740001	17	27	56	58

RPKM

	A1	A2	A3	A4
GSVIVT01022158001	599	478	250	240

RPKM

	A1	A2	A3	A4
GSVIVT01038252001	24	17	16	14
GSVIVT01009265001	47	56	57	30

RPKM

	A1	A2	A3	A4
GSVIVT01009755001	10	10	12	13
GSVIVT01013127001	7	7	12	8
GSVIVT01024352001	31	53	62	65

Pathway: UDP-α-D-glucuronate biosynthesis (from myo-inositol)

RPKM

	A1	A2	A3	A4
GSVIVT01015266001	1	0	0	0
GSVIVT01016929001	14	17	76	81
GSVIVT01016931001	1	0	0	0

RPKM

	A1	A2	A3	A4
GSVIVT01032385001	43	36	45	40

RPKM

	A1	A2	A3	A4
GSVIVT01008799001	124	157	266	215

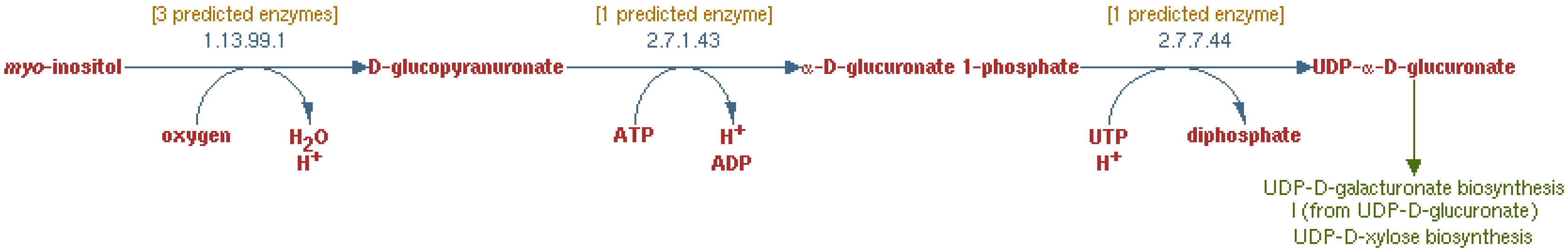

Pathway: stachyose degradation

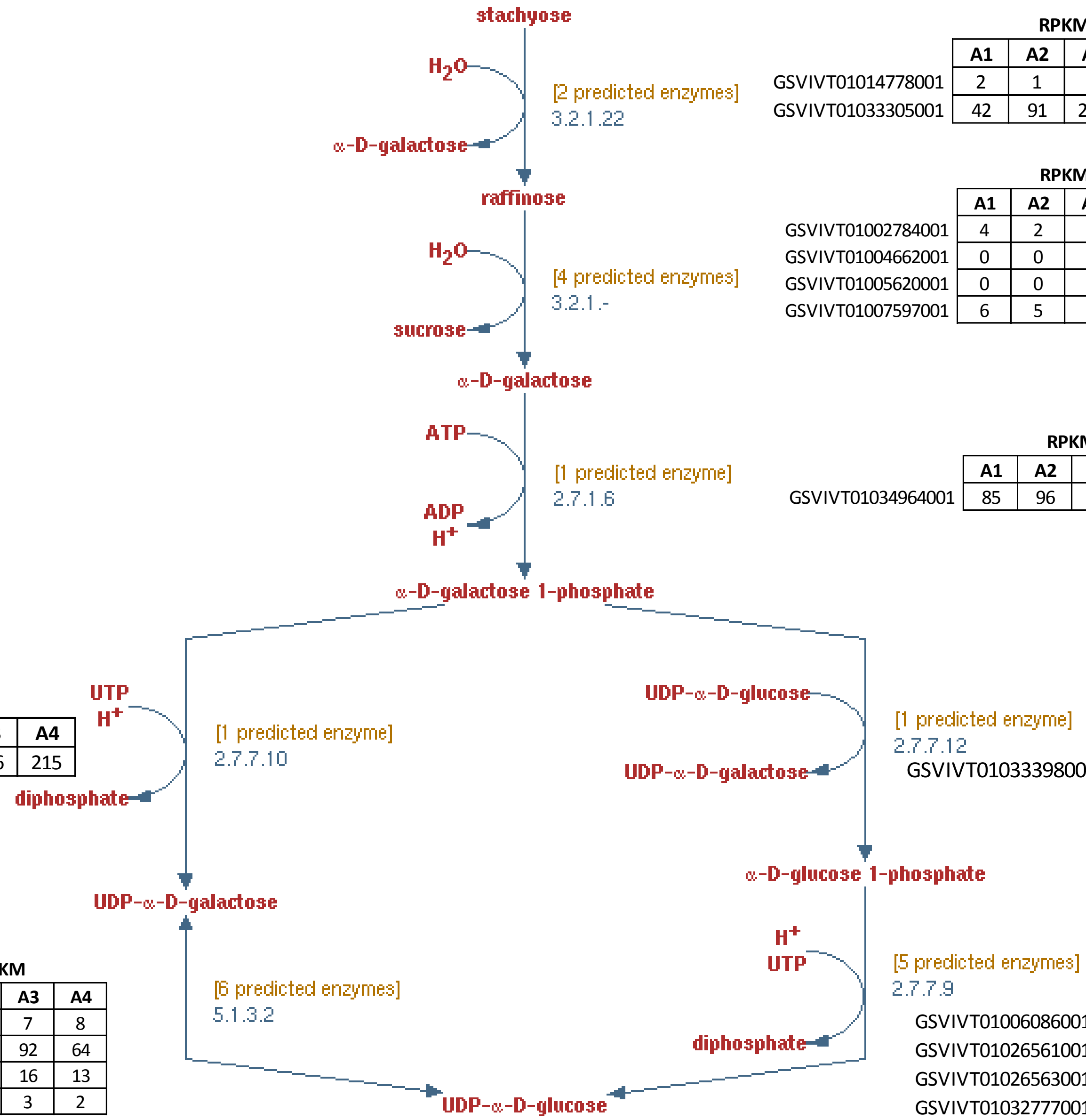

RPKM

	A1	A2	A3	A4
GSVIVT01014778001	2	1	1	1
GSVIVT01033305001	42	91	299	254

RPKM

	A1	A2	A3	A4
GSVIVT01002784001	4	2	1	1
GSVIVT01004662001	0	0	0	0
GSVIVT01005620001	0	0	0	0
GSVIVT01007597001	6	5	4	4

RPKM

	A1	A2	A3	A4
GSVIVT01034964001	85	96	139	200

RPKM

	A1	A2	A3	A4
GSVIVT01008799001	124	157	266	215

RPKM

	A1	A2	A3	A4
GSVIVT01033398001	13	11	19	22

RPKM

	A1	A2	A3	A4
GSVIVT01019545001	1	3	7	8
GSVIVT01019547001	82	105	92	64
GSVIVT01019833001	21	29	16	13
GSVIVT01024169001	6	5	3	2

RPKM

	A1	A2	A3	A4
GSVIVT01006086001	0	0	0	0
GSVIVT01026561001	0	0	0	0
GSVIVT01026563001	437	562	994	1047
GSVIVT01032777001	9	8	5	6
