# Supplementary material for: Transcriptome analysis during ripening of table grape berry cv. Thompson Seedless
Source: PLoS One. 2018 Jan 10;13(1):e0190087. doi: 10.1371/journal.pone.0190087 (PMC5761854; doi:10.1371/journal.pone.0190087)
Supplement: S3 Fig — (PDF) [file pone.0190087.s003.pdf]

Pathway: biotin-carboxyl carrier protein assembly

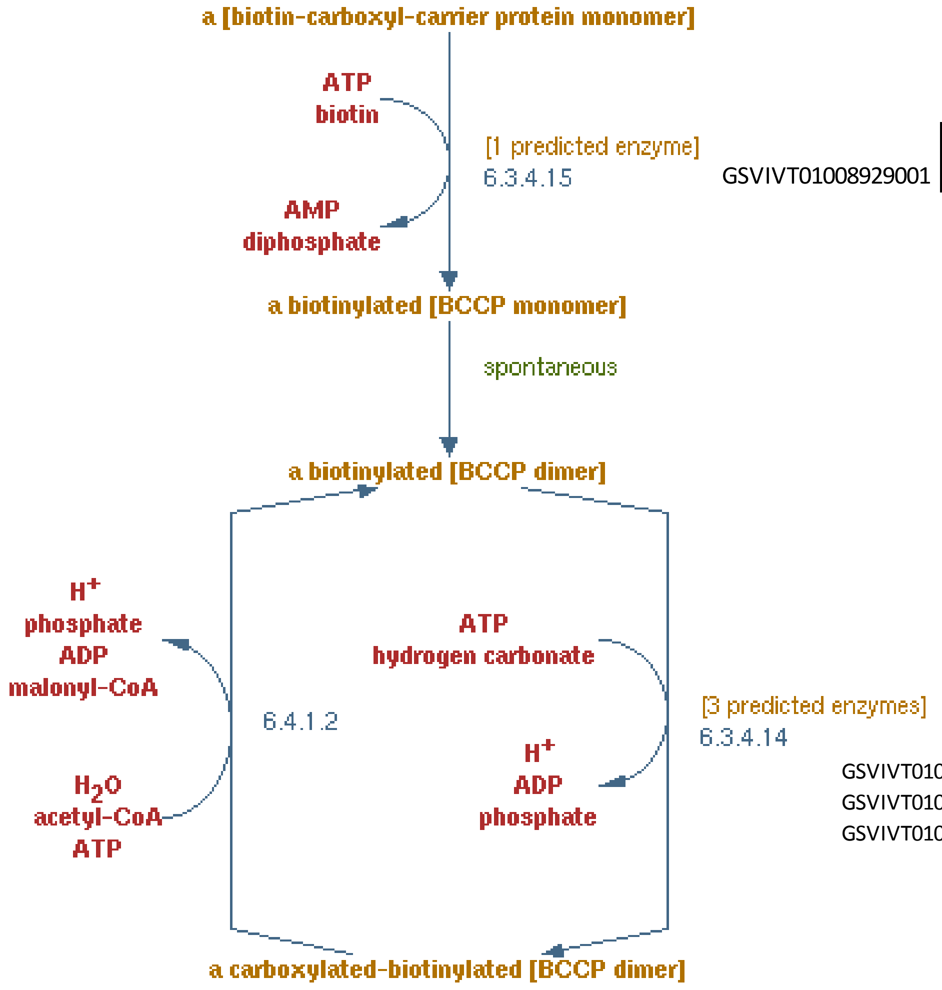

RPKM

	A1	A2	A3	A4
GSVIVT01008929001	51	44	41	39

RPKM

	A1	A2	A3	A4
GSVIVT01008991001	44	28	22	13
GSVIVT01032257001	39	32	38	28
GSVIVT01037979001	0	0	0	0

Pathway: fatty acid α-oxidation I

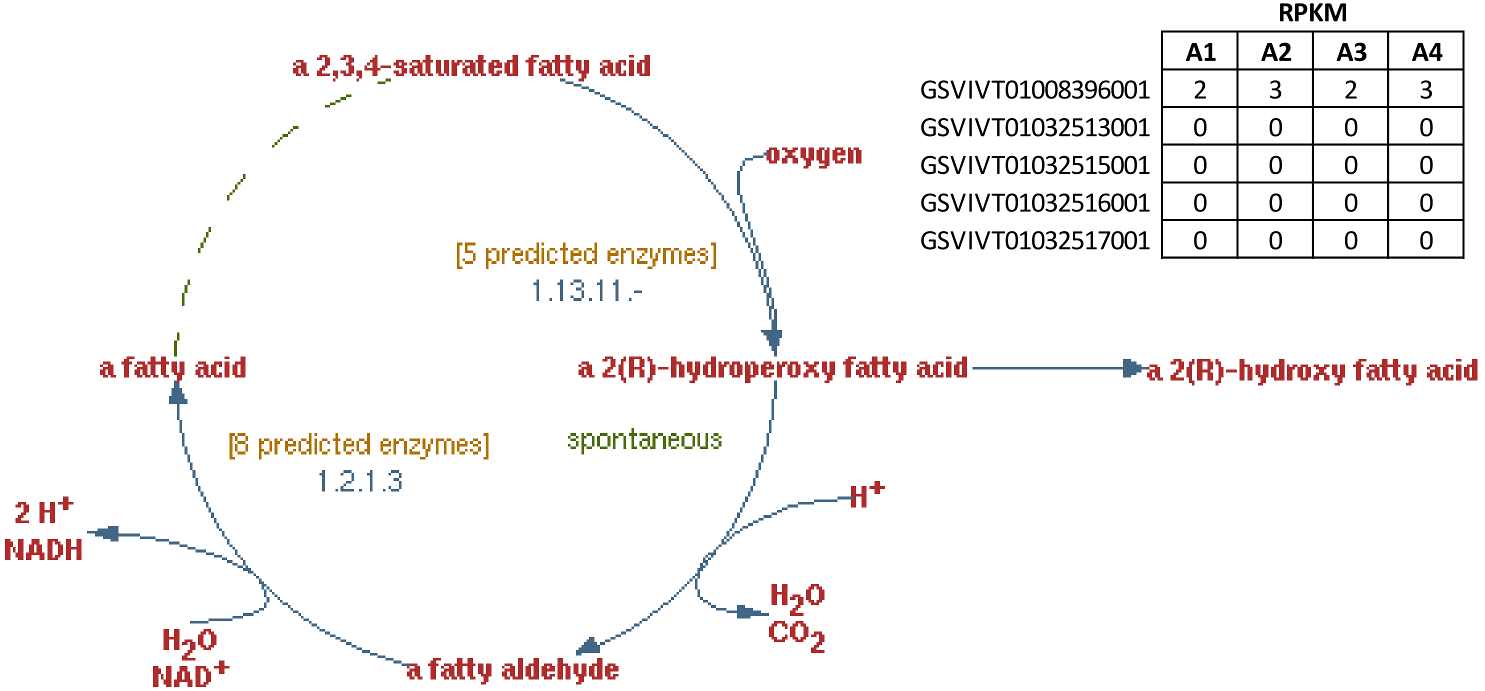

RPKM

	A1	A2	A3	A4
GSVIVT01008396001	2	3	2	3
GSVIVT01032513001	0	0	0	0
GSVIVT01032515001	0	0	0	0
GSVIVT01032516001	0	0	0	0
GSVIVT01032517001	0	0	0	0

RPKM

	A1	A2	A3	A4
GSVIVT01008845001	42	39	19	15
GSVIVT01022356001	32	34	65	102

Pathway: phosphatidylethanolamine biosynthesis I

RPKM

	A1	A2	A3	A4
GSVIVT01008367001	8	6	2	2
GSVIVT01011808001	10	12	35	38

RPKM

	A1	A2	A3	A4
GSVIVT01004724001	5	4	3	3
GSVIVT01010110001	0	0	0	0
GSVIVT01029732001	5	5	6	6
GSVIVT01029733001	20	16	15	15
GSVIVT01029735001	68	53	31	19
GSVIVT01038340001	0	0	0	0

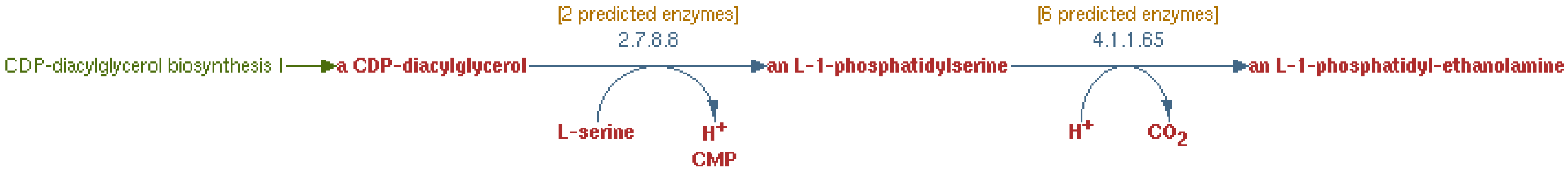

Pathway: homoserine biosynthesis

RPKM

	A1	A2	A3	A4
GSVIVT01004941001	6	4	3	2
GSVIVT01023985001	7	2	1	1
GSVIVT01032996001	24	28	20	9
GSVIVT01036290001	47	35	26	17
GSVIVT01038492001	0	0	0	0

RPKM

	A1	A2	A3	A4
GSVIVT01011646001	19	23	18	19

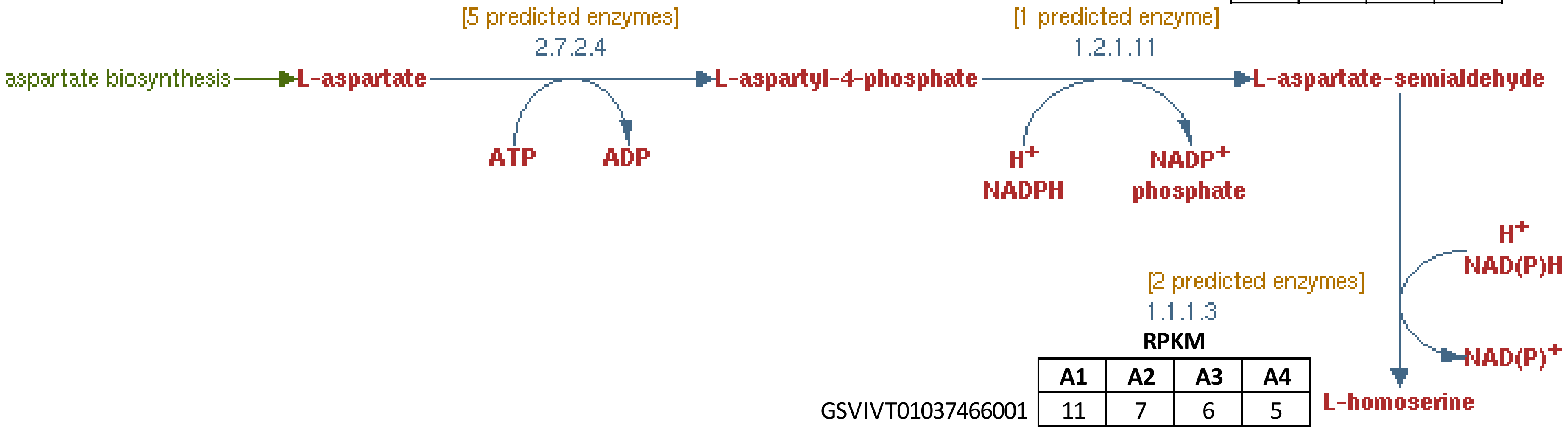

RPKM

	A1	A2	A3	A4
GSVIVT01037466001	11	7	6	5

Pathway: methionine degradation I (to homocysteine)

RPKM

	A1	A2	A3	A4
GSVIVT01007568001	0	0	0	0
GSVIVT01017675001	261	563	718	882
GSVIVT01026821001	23	18	14	10
GSVIVT01028205001	167	237	231	165
GSVIVT01031313001	1048	1341	1609	2366
GSVIVT01031492001	0	0	0	0
GSVIVT01033651001	6	12	24	20
GSVIVT01036663001	0	0	0	0

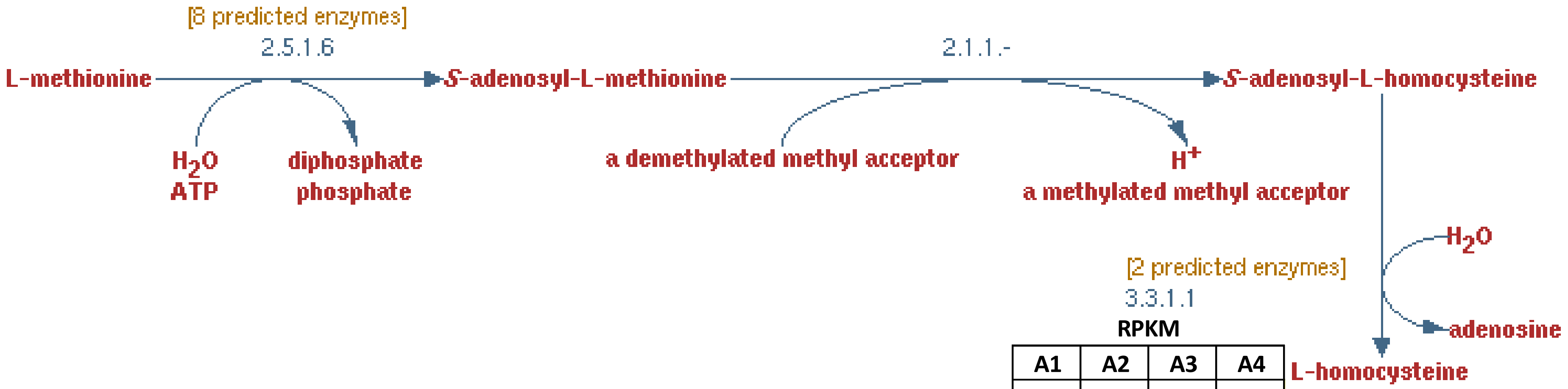

RPKM

	A1	A2	A3	A4
GSVIVT01007578001	185	290	292	313
GSVIVT01021041001	644	1201	1699	1700

Pathway: S-methyl-5-thio-α-D-ribose 1-phosphate degradation

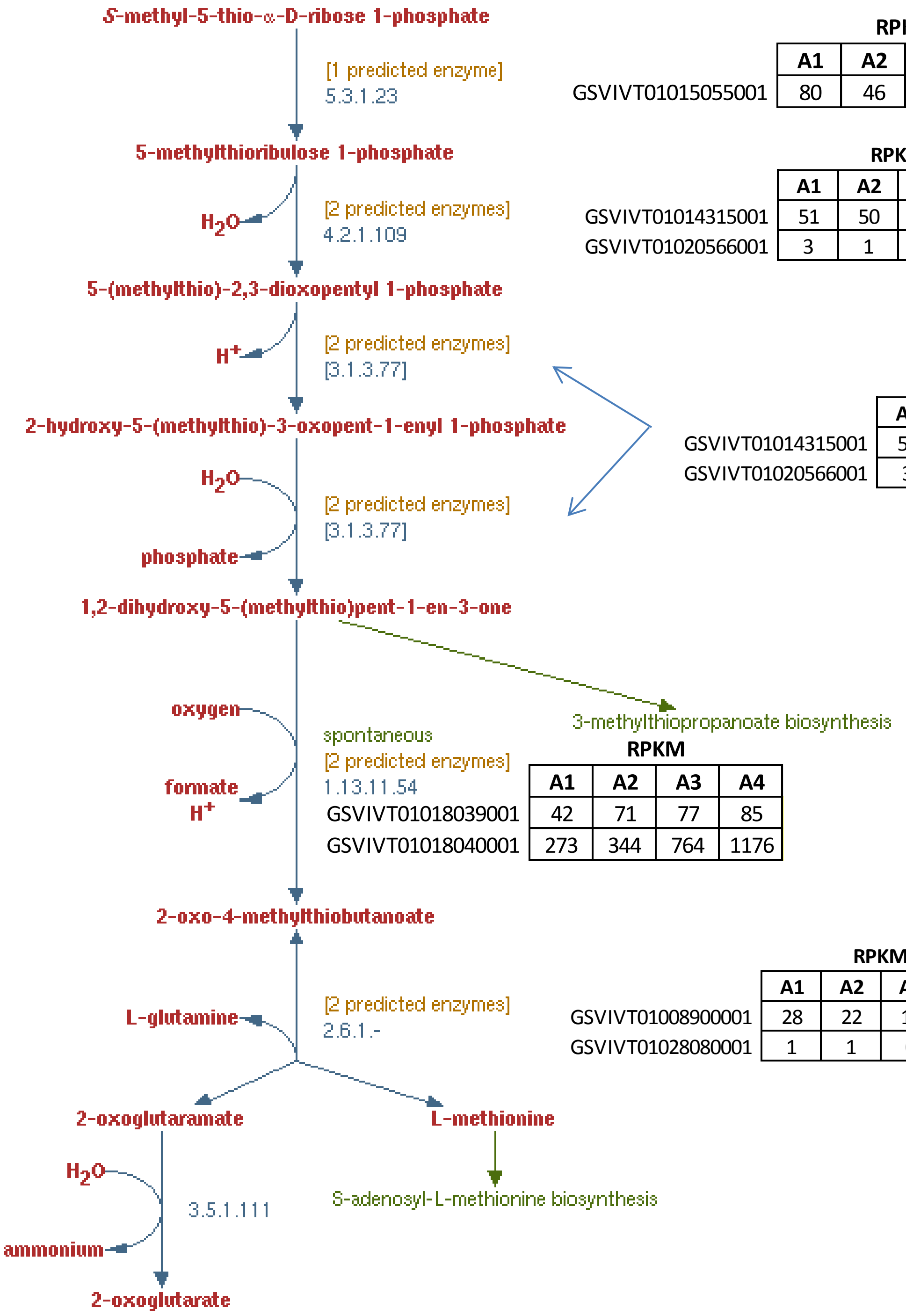

RPKM

	A1	A2	A3	A4
GSVIVT01015055001	80	46	37	41

RPKM

	A1	A2	A3	A4
GSVIVT01014315001	51	50	55	70
GSVIVT01020566001	3	1	1	2

RPKM

	A1	A2	A3	A4
GSVIVT01014315001	51	50	55	70
GSVIVT01020566001	3	1	1	2

RPKM

	A1	A2	A3	A4
GSVIVT01018039001	42	71	77	85
GSVIVT01018040001	273	344	764	1176

RPKM

	A1	A2	A3	A4
GSVIVT01008900001	28	22	18	21
GSVIVT01028080001	1	1	0	0

Pathway: xylan biosynthesis

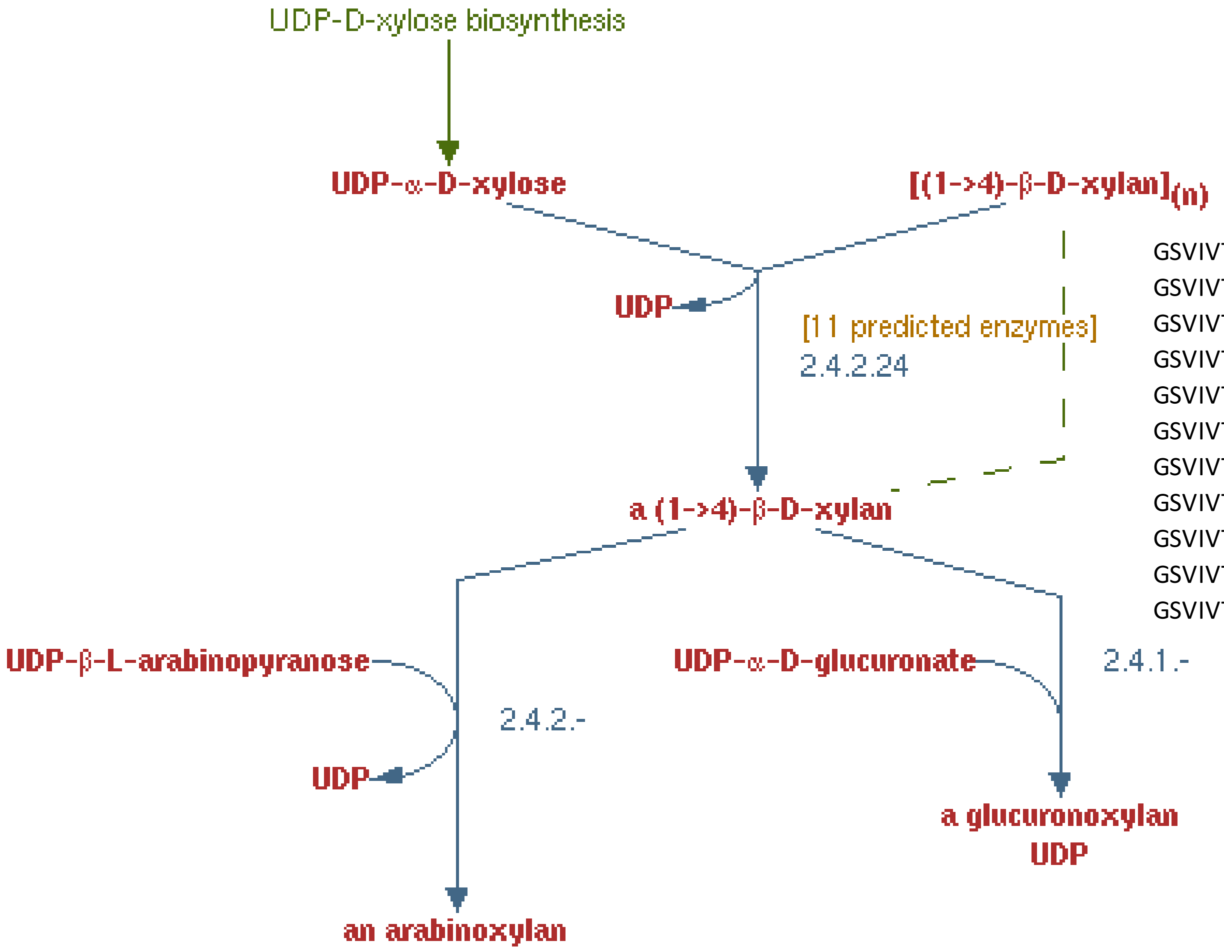

RPKM

	A1	A2	A3	A4
GSVIVT01009245001	45	62	64	44
GSVIVT01010090001	0	0	0	0
GSVIVT01013057001	2	9	29	20
GSVIVT01018559001	0	0	0	0
GSVIVT01021798001	0	0	0	0
GSVIVT01022064001	34	31	27	21
GSVIVT01023850001	0	0	0	0
GSVIVT01024398001	40	63	58	28
GSVIVT01025636001	4	4	2	3
GSVIVT01028071001	2	0	0	0
GSVIVT01035647001	11	10	7	3

Pathway: sucrose degradation III (sucrose invertase)

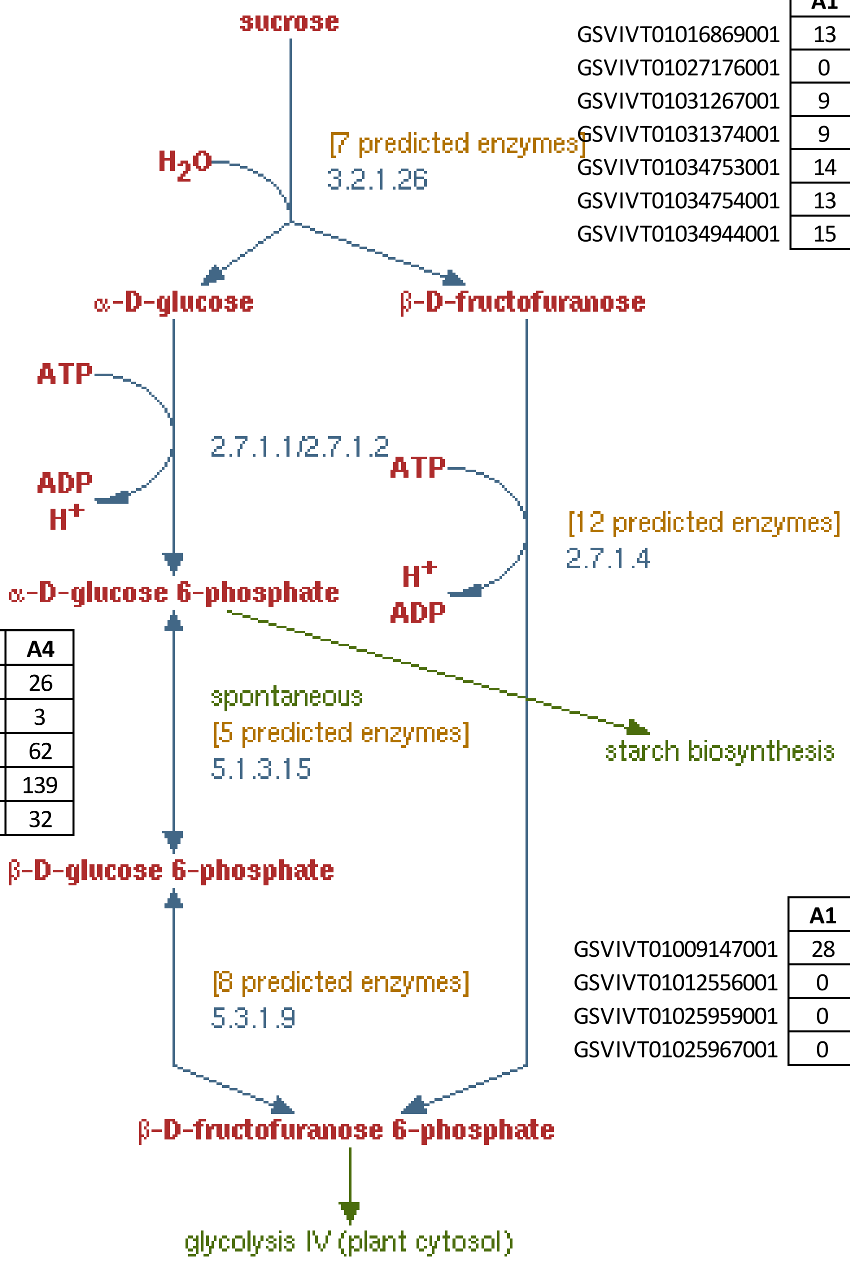

RPKM

	A1	A2	A3	A4
GSVIVT01016869001	13	4	2	8
GSVIVT01027176001	0	0	0	0
GSVIVT01031267001	9	16	25	22
GSVIVT01031374001	9	10	11	11
GSVIVT01034753001	14	22	20	9
GSVIVT01034754001	13	26	33	32
GSVIVT01034944001	15	11	11	14

RPKM

	A1	A2	A3	A4
GSVIVT01000761001	14	12	13	8
GSVIVT01002667001	0	0	0	0
GSVIVT01006129001	0	0	0	0
GSVIVT01009899001	51	26	8	5
GSVIVT01010790001	21	6	2	3
GSVIVT01012237001	11	9	8	8
GSVIVT01018449001	13	20	22	21
GSVIVT01027578001	3	2	1	1
GSVIVT01030838001	42	31	24	25
GSVIVT01031551001	12	10	14	17
GSVIVT01034028001	28	26	15	14
GSVIVT01037102001	1	0	0	1

RPKM

	A1	A2	A3	A4
GSVIVT01018938001	15	16	15	26
GSVIVT01019482001	13	5	3	3
GSVIVT01027042001	26	23	32	62
GSVIVT01029239001	177	139	136	139
GSVIVT01032393001	103	84	42	32

RPKM

	A1	A2	A3	A4
GSVIVT01009147001	28	29	36	41
GSVIVT01012556001	0	0	0	0
GSVIVT01025959001	0	0	0	0
GSVIVT01025967001	0	0	0	0
