# Supplementary material for: Transcriptome analysis during ripening of table grape berry cv. Thompson Seedless
Source: PLoS One. 2018 Jan 10;13(1):e0190087. doi: 10.1371/journal.pone.0190087 (PMC5761854; doi:10.1371/journal.pone.0190087)
Supplement: S4 Fig — (PDF) [file pone.0190087.s004.pdf]

Pathway: aerobic respiration III (alternative oxidase pathway)

RPKM

	A1	A2	A3	A4
GSVIVT01000609001	63	78	94	135
GSVIVT01000718001	111	114	123	139
GSVIVT01002385001	0	0	0	0
GSVIVT01002443001	0	0	0	0
GSVIVT01004403001	0	0	0	0
GSVIVT01004958001	0	0	0	0
GSVIVT01004963001	0	0	0	0
GSVIVT01004966001	4	3	4	7
GSVIVT01004967001	1	0	1	1
GSVIVT01004976001	1	0	0	1
GSVIVT01006222001	0	0	0	0
GSVIVT01007203001	1	2	3	7
GSVIVT01008282001	542	648	1001	1390
GSVIVT01009735001	215	233	270	318
GSVIVT01010076001	30	45	57	110
GSVIVT01010482001	70	107	137	341
GSVIVT01010679001	103	86	106	171
GSVIVT01010904001	107	119	189	224
GSVIVT01011707001	8	7	2	1
GSVIVT01012613001	0	0	0	0
GSVIVT01013142001	3	2	5	6
GSVIVT01013345001	0	0	0	0

RPKM

	A1	A2	A3	A4
GSVIVT01013481001	0	0	0	0
GSVIVT01018929001	0	0	0	0
GSVIVT01020401001	129	164	227	293
GSVIVT01023768001	0	0	0	0
GSVIVT01024414001	63	59	72	94
GSVIVT01028438001	83	87	86	70
GSVIVT01028486001	85	90	112	125
GSVIVT01028820001	20	21	27	34
GSVIVT01029664001	1	1	1	1
GSVIVT01029731001	51	45	70	63
GSVIVT01029923001	0	0	0	0
GSVIVT01030914001	98	118	167	155
GSVIVT01031966001	96	111	142	177
GSVIVT01033348001	16	20	30	44
GSVIVT01035146001	48	63	81	96
GSVIVT01035355001	43	57	130	186
GSVIVT01036114001	165	242	528	712
GSVIVT01036643001	138	155	342	422
GSVIVT01036674001	0	0	0	0
GSVIVT01036676001	0	0	0	0
GSVIVT01037779001	59	82	97	133

RPKM

	A1	A2	A3	A4
GSVIVT01006689001	6	6	8	8
GSVIVT01007272001	3	3	7	8
GSVIVT01022812001	1	4	1	5
GSVIVT01022814001	10	17	20	41
GSVIVT01038745001	35	34	58	85

RPKM

	A1	A2	A3	A4
GSVIVT01007813001	35	38	57	75
GSVIVT01009527001	66	66	76	95
GSVIVT01018976001	70	69	75	69
GSVIVT01027061001	79	80	84	118
GSVIVT01028464001	242	310	381	473

[46 predicted enzymes]
1.6.5.3

[6 predicted enzymes]
1.10.3.11

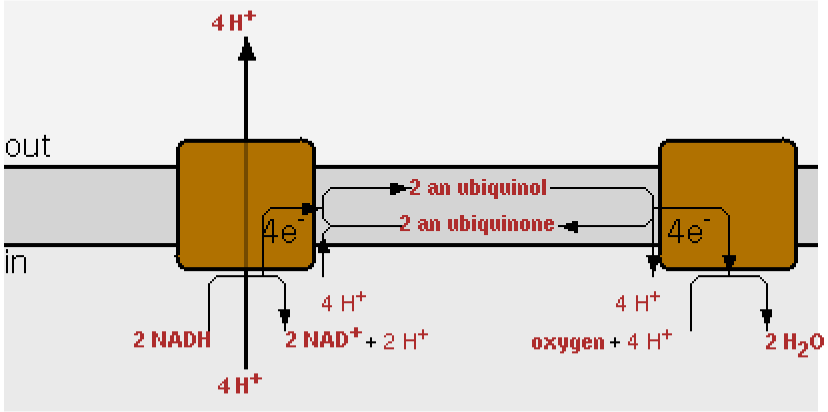

[9 predicted enzymes]
1.3.5.1

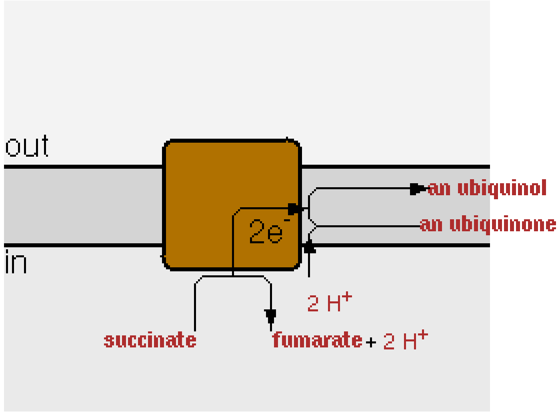

Pathway: TCA cycle II (plants and fungi)

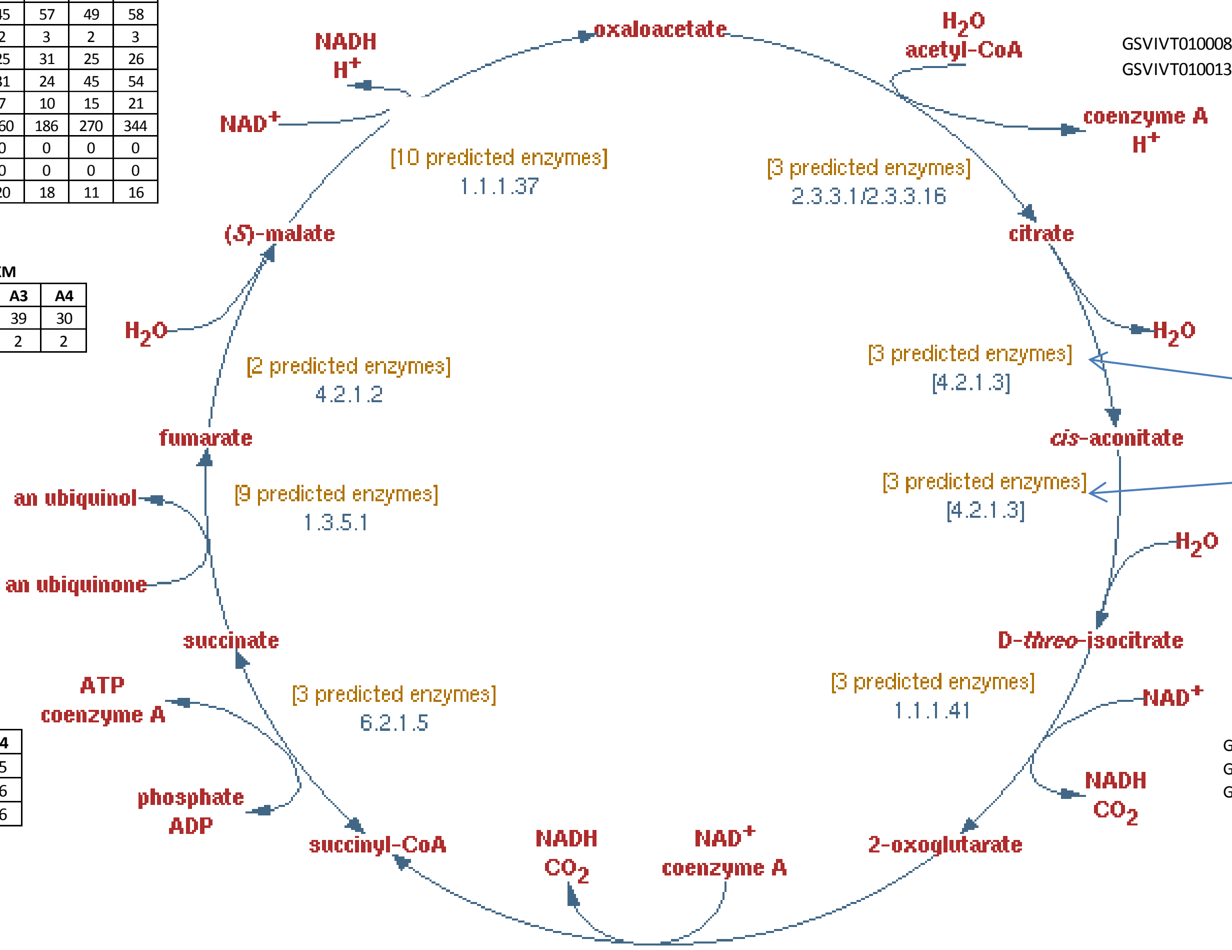

RPKM

	A1	A2	A3	A4
GSVIVT01006474001	53	39	60	62
GSVIVT01007997001	45	57	49	58
GSVIVT01010395001	2	3	2	3
GSVIVT01011422001	25	31	25	26
GSVIVT01014231001	31	24	45	54
GSVIVT01018190001	7	10	15	21
GSVIVT01021185001	160	186	270	344
GSVIVT01028330001	0	0	0	0
GSVIVT01028332001	0	0	0	0
GSVIVT01036965001	20	18	11	16

RPKM

	A1	A2	A3	A4
GSVIVT01000817001	38	45	55	51
GSVIVT01001358001	71	77	98	98

RPKM

	A1	A2	A3	A4
GSVIVT01028056001	46	43	39	30
GSVIVT01031185001	3	1	2	2

RPKM

	A1	A2	A3	A4
GSVIVT01005190001	122	108	100	81
GSVIVT01030588001	13	10	5	3
GSVIVT01037657001	90	94	138	122

RPKM

	A1	A2	A3	A4
GSVIVT01007813001	35	38	57	75
GSVIVT01009527001	66	66	76	95
GSVIVT01018976001	70	69	75	69
GSVIVT01027061001	79	80	84	118
GSVIVT01028464001	242	310	381	473

RPKM

	A1	A2	A3	A4
GSVIVT01026227001	19	20	17	15
GSVIVT01028373001	60	63	88	86
GSVIVT01029337001	42	50	58	66

RPKM

	A1	A2	A3	A4
GSVIVT01003837001	27	24	32	44
GSVIVT01023949001	44	57	63	65
GSVIVT01025704001	72	79	96	90

Pathway: phosphate acquisition

	RPKM			
	A1	A2	A3	A4
GSVIVT01000004001	15	11	4	7
GSVIVT01005232001	6	6	9	13
GSVIVT01009802001	10	11	14	11
GSVIVT01009803001	17	20	15	23
GSVIVT01011599001	14	41	44	83
GSVIVT01012830001	34	37	48	48
GSVIVT01014022001	11	18	26	32
GSVIVT01015277001	6	4	9	21
GSVIVT01016112001	0	0	0	0
GSVIVT01016377001	61	64	69	83
GSVIVT01016957001	0	0	0	0
GSVIVT01021054001	0	0	0	0
GSVIVT01024212001	0	0	0	0
GSVIVT01024214001	0	0	0	0
GSVIVT01024216001	0	0	0	0
GSVIVT01028404001	9	27	5	1
GSVIVT01029053001	13	14	46	43
GSVIVT01029880001	1	1	0	3
GSVIVT01031373001	22	18	17	19
GSVIVT01033822001	0	0	0	0
GSVIVT01033823001	0	0	0	0
GSVIVT01035956001	45	50	59	45
GSVIVT01037630001	107	124	129	167

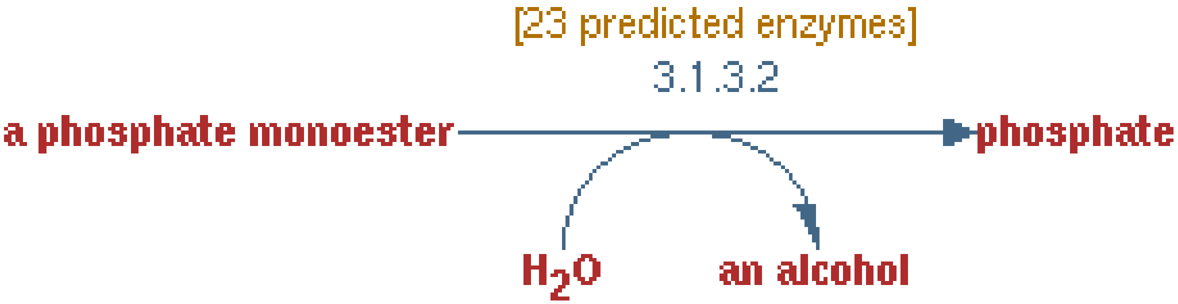

Pathway: glutamate degradation IV

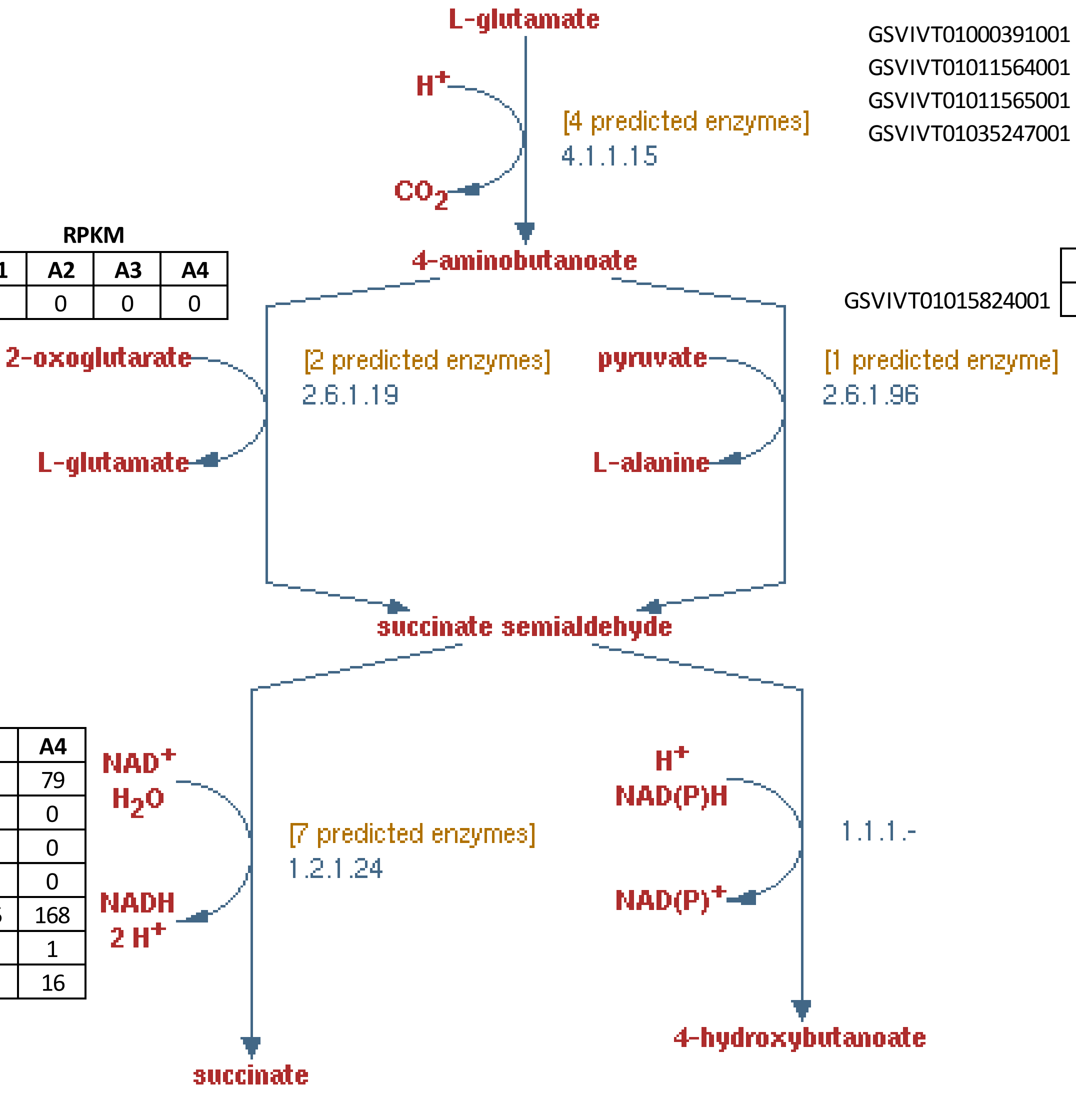

RPKM

	A1	A2	A3	A4
GSVIVT01000391001	203	169	303	353
GSVIVT01011564001	2	1	0	0
GSVIVT01011565001	4	4	5	7
GSVIVT01035247001	422	427	334	372

RPKM

	A1	A2	A3	A4
GSVIVT01015821001	0	0	0	0

RPKM

	A1	A2	A3	A4
GSVIVT01015824001	86	94	103	118

RPKM

	A1	A2	A3	A4
GSVIVT01004121001	56	64	64	79
GSVIVT01006061001	0	0	0	0
GSVIVT01036678001	0	0	0	0
GSVIVT01036681001	0	0	0	0
GSVIVT01036719001	345	298	205	168
GSVIVT01036720001	1	1	1	1
GSVIVT01036721001	36	36	23	16
