# Supplementary material for: Transcriptome analysis during ripening of table grape berry cv. Thompson Seedless
Source: PLoS One. 2018 Jan 10;13(1):e0190087. doi: 10.1371/journal.pone.0190087 (PMC5761854; doi:10.1371/journal.pone.0190087)
Supplement: S5 Fig — (PDF) [file pone.0190087.s005.pdf]

Pathway: fatty acid biosynthesis initiation I

RPKM

	A1	A2	A3	A4
GSVIVT01003458001	0	0	0	0
GSVIVT01003465001	0	0	0	0
GSVIVT01008991001	44	28	22	13
GSVIVT01010035001	28	22	24	20
GSVIVT01011511001	4	2	0	0
GSVIVT01011513001	1	0	0	0
GSVIVT01017253001	23	31	22	21
GSVIVT01019068001	29	37	48	55
GSVIVT01022888001	0	0	0	0
GSVIVT01026852001	28	29	22	21
GSVIVT01032257001	39	32	38	28
GSVIVT01034017001	28	26	22	19
GSVIVT01037256001	0	0	0	0

RPKM

	A1	A2	A3	A4
GSVIVT01024638001	27	21	17	12
GSVIVT01024639001	17	21	21	22

RPKM

	A1	A2	A3	A4
GSVIVT01001278001	7	8	5	3
GSVIVT01004779001	0	0	0	0

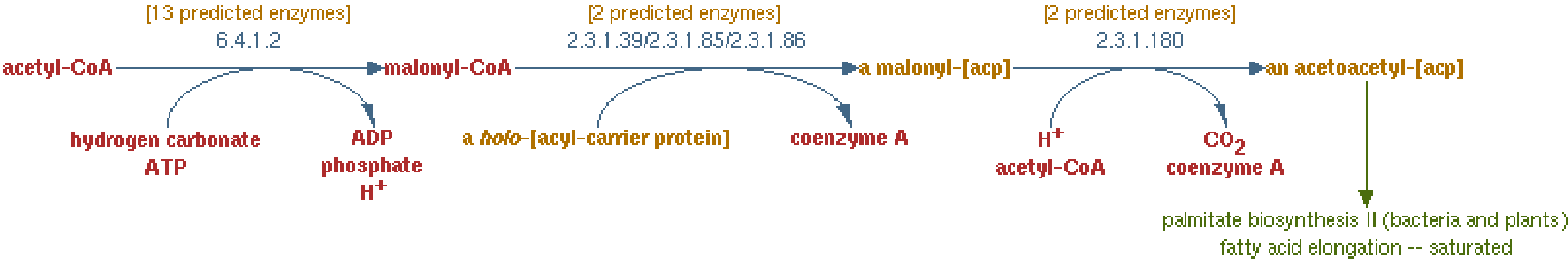

Pathway: 3-phosphoinositide biosynthesis

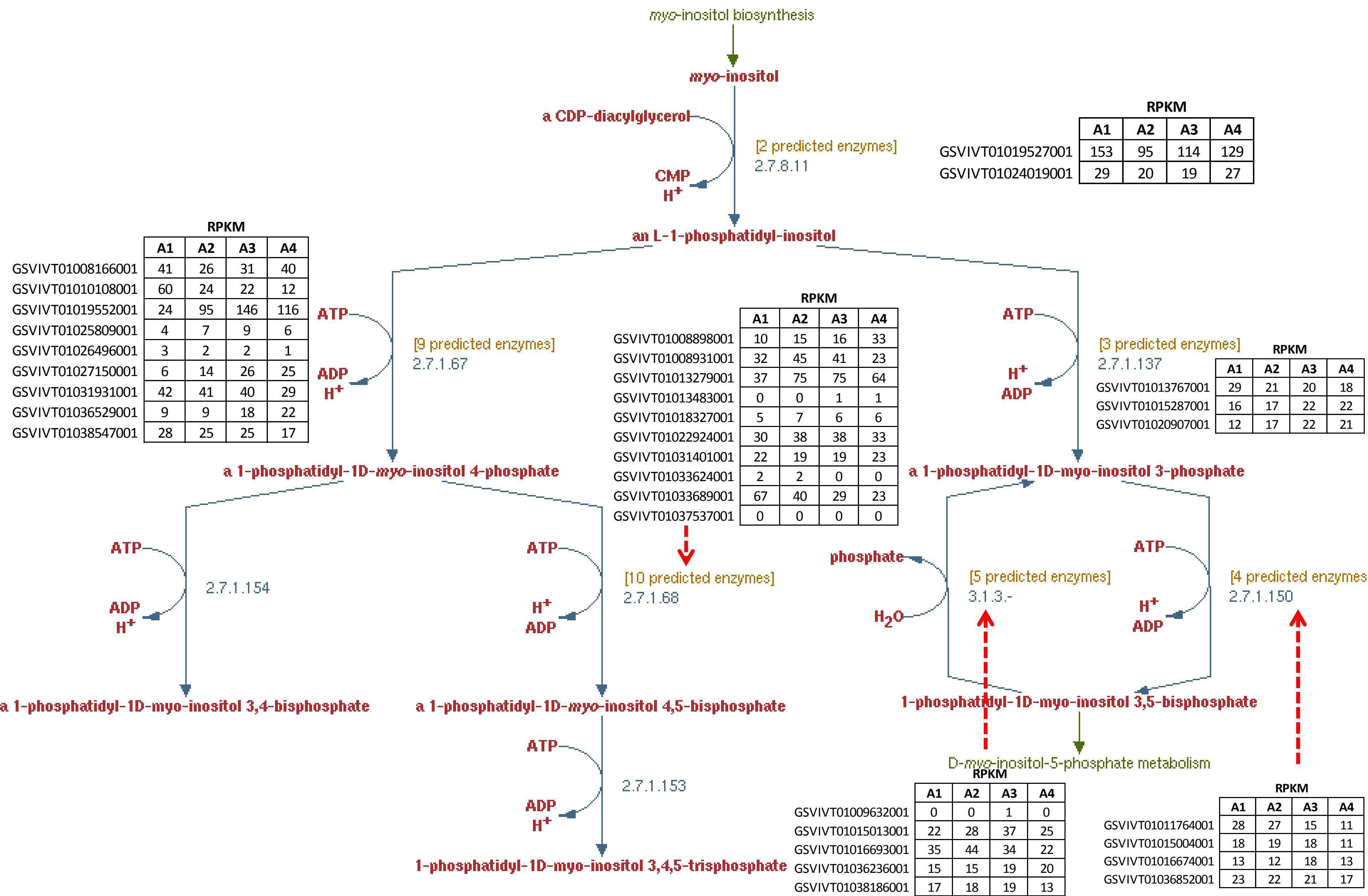

RPKM

	A1	A2	A3	A4
GSVIVT01019527001	153	95	114	129
GSVIVT01024019001	29	20	19	27

RPKM

	A1	A2	A3	A4
GSVIVT01008166001	41	26	31	40
GSVIVT01010108001	60	24	22	12
GSVIVT01019552001	24	95	146	116
GSVIVT01025809001	4	7	9	6
GSVIVT01026496001	3	2	2	1
GSVIVT01027150001	6	14	26	25
GSVIVT01031931001	42	41	40	29
GSVIVT01036529001	9	9	18	22
GSVIVT01038547001	28	25	25	17

RPKM

	A1	A2	A3	A4
GSVIVT01008898001	10	15	16	33
GSVIVT01008931001	32	45	41	23
GSVIVT01013279001	37	75	75	64
GSVIVT01013483001	0	0	1	1
GSVIVT01018327001	5	7	6	6
GSVIVT01022924001	30	38	38	33
GSVIVT01031401001	22	19	19	23
GSVIVT01033624001	2	2	0	0
GSVIVT01033689001	67	40	29	23
GSVIVT01037537001	0	0	0	0

RPKM

	A1	A2	A3	A4
GSVIVT01013767001	29	21	20	18
GSVIVT01015287001	16	17	22	22
GSVIVT01020907001	12	17	22	21

RPKM

	A1	A2	A3	A4
GSVIVT01009632001	0	0	1	0
GSVIVT01015013001	22	28	37	25
GSVIVT01016693001	35	44	34	22
GSVIVT01036236001	15	15	19	20
GSVIVT01038186001	17	18	19	13

RPKM

	A1	A2	A3	A4
GSVIVT01011764001	28	27	15	11
GSVIVT01015004001	18	19	18	11
GSVIVT01016674001	13	12	18	13
GSVIVT01036852001	23	22	21	17

Pathway: diacylglycerol biosynthesis (PUFA enrichment in oilseed)

RPKM

	A1	A2	A3	A4
GSVIVT01017341001	2	2	2	1

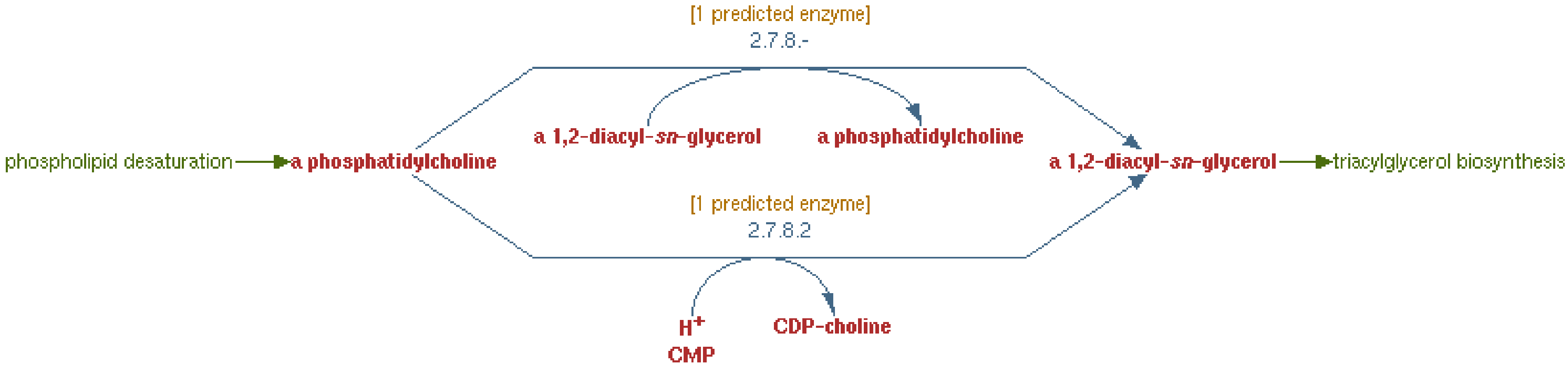

RPKM

	A1	A2	A3	A4
GSVIVT01010154001	66	68	63	53
