# Supplementary figures and images for: Transcriptome analysis during ripening of table grape berry cv. Thompson Seedless
Source: PLoS One. 2018 Jan 10;13(1):e0190087. doi: 10.1371/journal.pone.0190087 (PMC5761854; doi:10.1371/journal.pone.0190087)

# Pathway: pyruvate decarboxylation to acetyl CoA

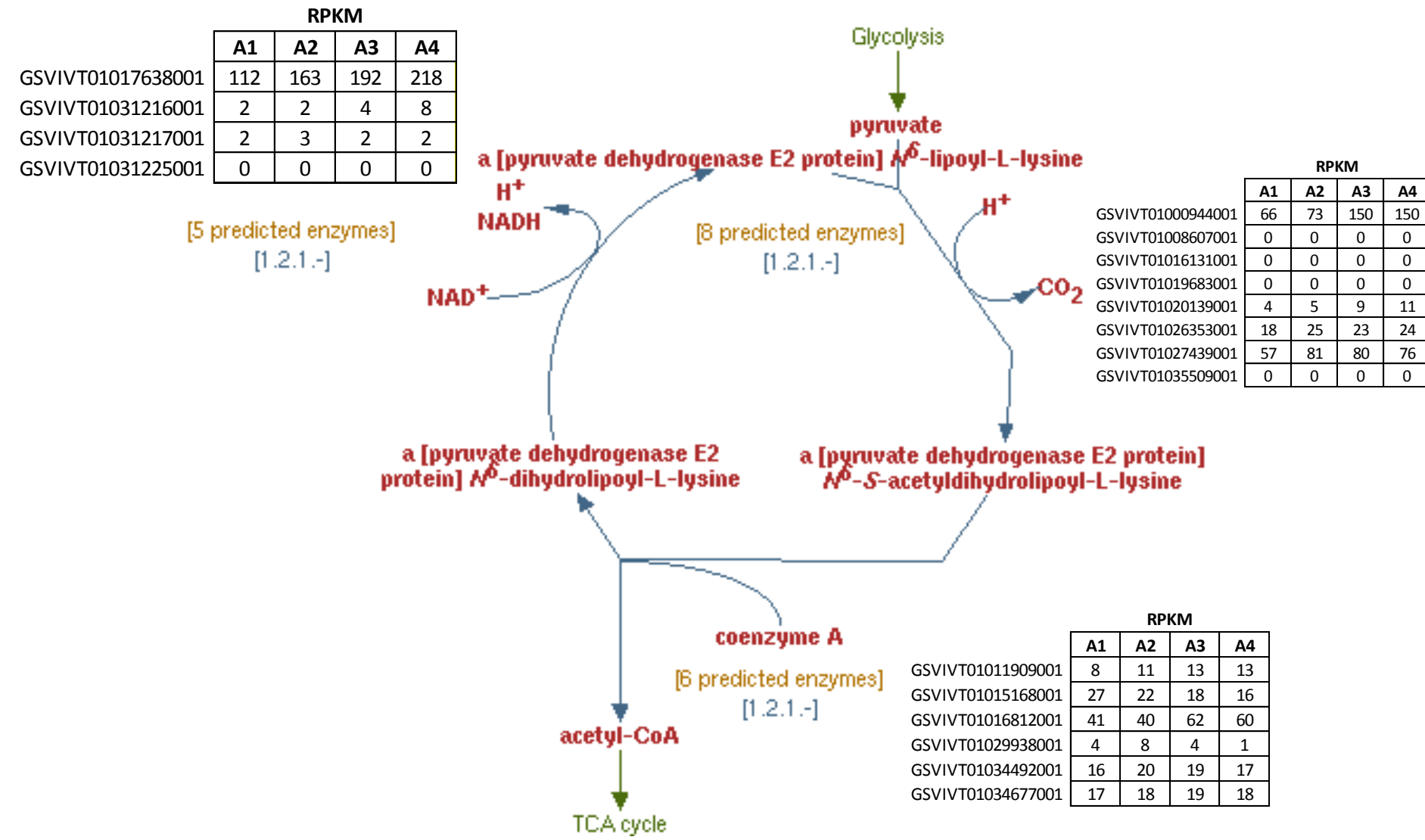

# Pathway: triacylglycerol biosynthesis

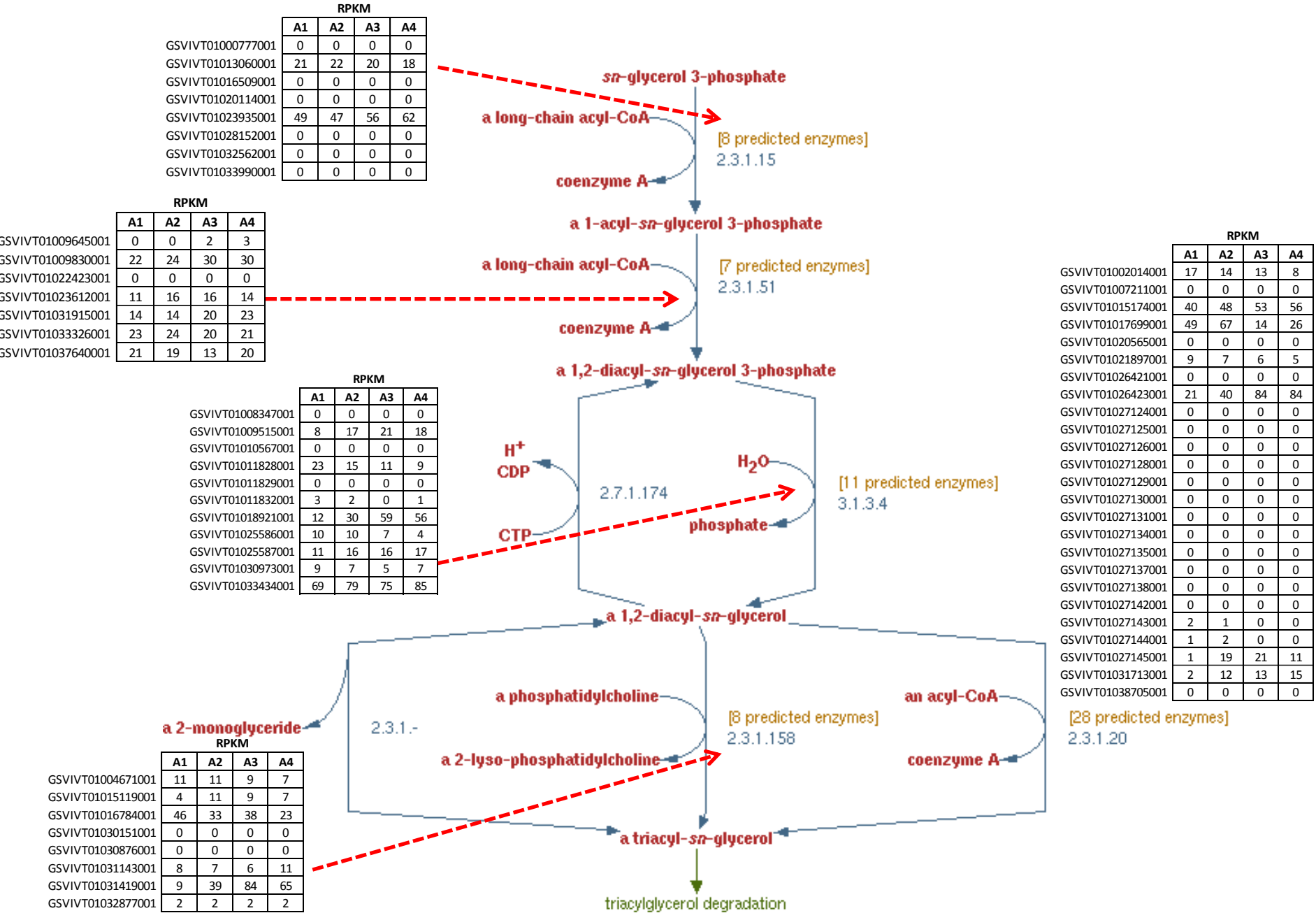

Supplement: S6 Fig — (PDF) [file pone.0190087.s006.pdf]
