# Supplementary material for: Transcriptome analysis during ripening of table grape berry cv. Thompson Seedless
Source: PLoS One. 2018 Jan 10;13(1):e0190087. doi: 10.1371/journal.pone.0190087 (PMC5761854; doi:10.1371/journal.pone.0190087)

# Pathway: serine racemization

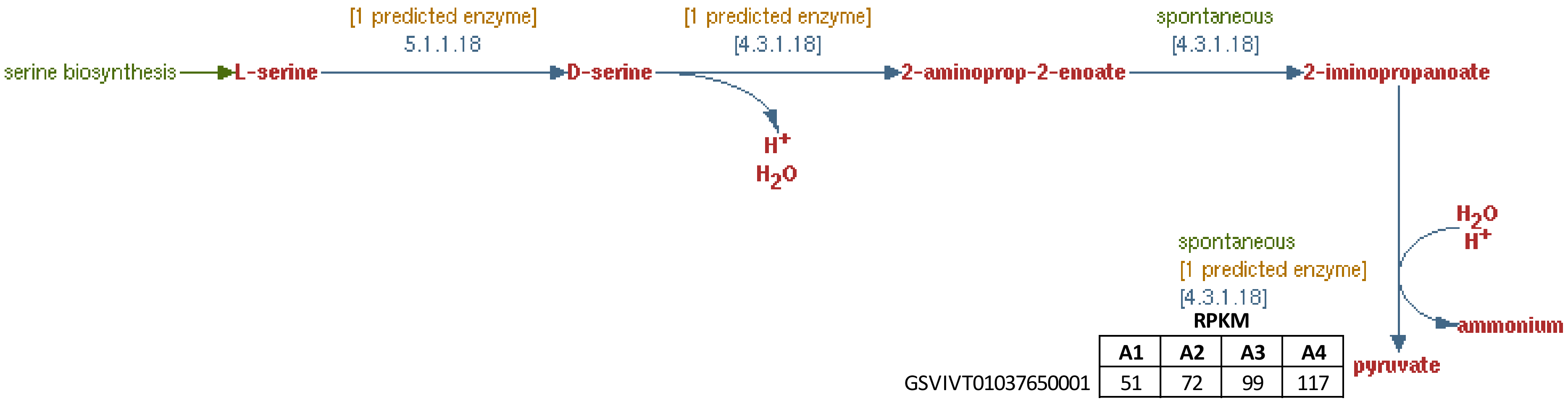


RPKM

| | A1 | A2 | A3 | A4 |
|---|---|---|---|---|
| GSVIVT01019772001 | 10 | 8 | 7 | 10 |

RPKM

| | A1 | A2 | A3 | A4 |
|---|---|---|---|---|
| GSVIVT01019772001 | 10 | 8 | 7 | 10 |

RPKM

| | A1 | A2 | A3 | A4 |
|---|---|---|---|---|
| GSVIVT01037650001 | 51 | 72 | 99 | 117 |

# Pathway: glutamate removal from folates

| | RPKM | | | |
|---|---|---|---|---|
| | A1 | A2 | A3 | A4 |
| GSVIVT01014167001 | 107 | 74 | 73 | 123 |

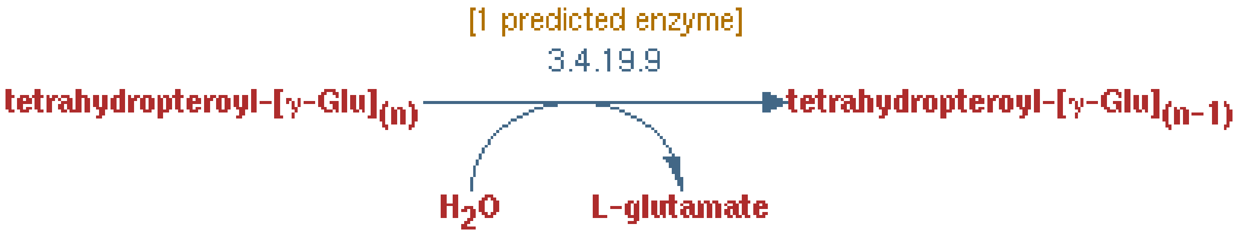

# Pathway: sulfite oxidation IV

| | RPKM | | | |
|---|---|---|---|---|
| | A1 | A2 | A3 | A4 |
| GSVIVT01033099001 | 110 | 94 | 92 | 109 |

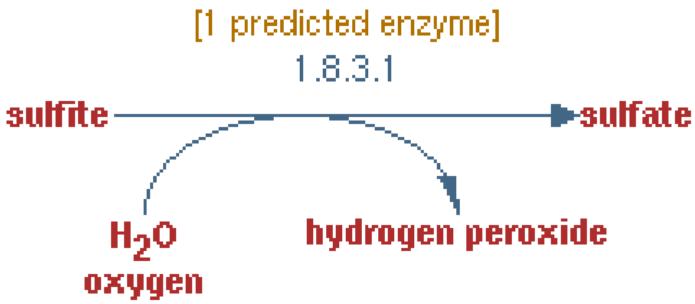

Supplement: S7 Fig — (PDF) [file pone.0190087.s007.pdf]
