# Supplementary material for: Transcriptome analysis during ripening of table grape berry cv. Thompson Seedless
Source: PLoS One. 2018 Jan 10;13(1):e0190087. doi: 10.1371/journal.pone.0190087 (PMC5761854; doi:10.1371/journal.pone.0190087)

Pathway: acyl-CoA hydrolysis

RPKM

	A1	A2	A3	A4
GSVIVT01000239001	12	25	30	32
GSVIVT01000240001	25	28	23	30
GSVIVT01002016001	0	0	0	0
GSVIVT01004130001	0	0	0	0
GSVIVT01004133001	22	23	24	26
GSVIVT01017055001	1	2	1	2
GSVIVT01024800001	59	57	37	42
GSVIVT01026913001	34	27	31	29
GSVIVT01027494001	15	12	11	11
GSVIVT01027495001	18	18	31	44
GSVIVT01027496001	3	2	1	2
GSVIVT01038195001	21	19	13	9

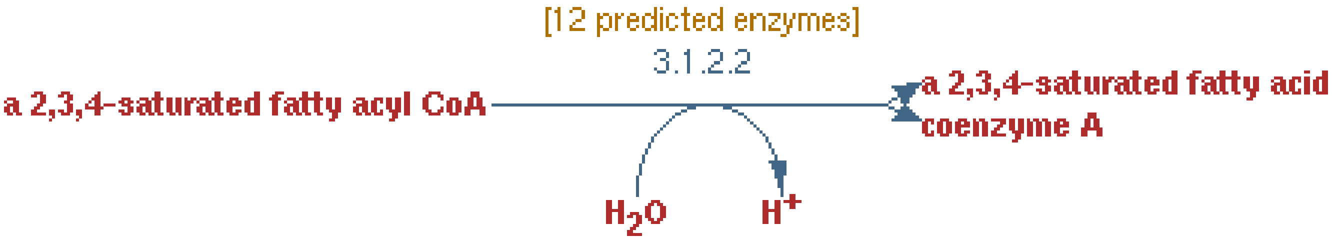

Supplement: S8 Fig — (PDF) [file pone.0190087.s008.pdf]
