# Supplementary material for: Transcriptome analysis during ripening of table grape berry cv. Thompson Seedless
Source: PLoS One. 2018 Jan 10;13(1):e0190087. doi: 10.1371/journal.pone.0190087 (PMC5761854; doi:10.1371/journal.pone.0190087)
Supplement: S9 Fig — (PDF) [file pone.0190087.s009.pdf]

Pathway: chlorophyll a degradation I

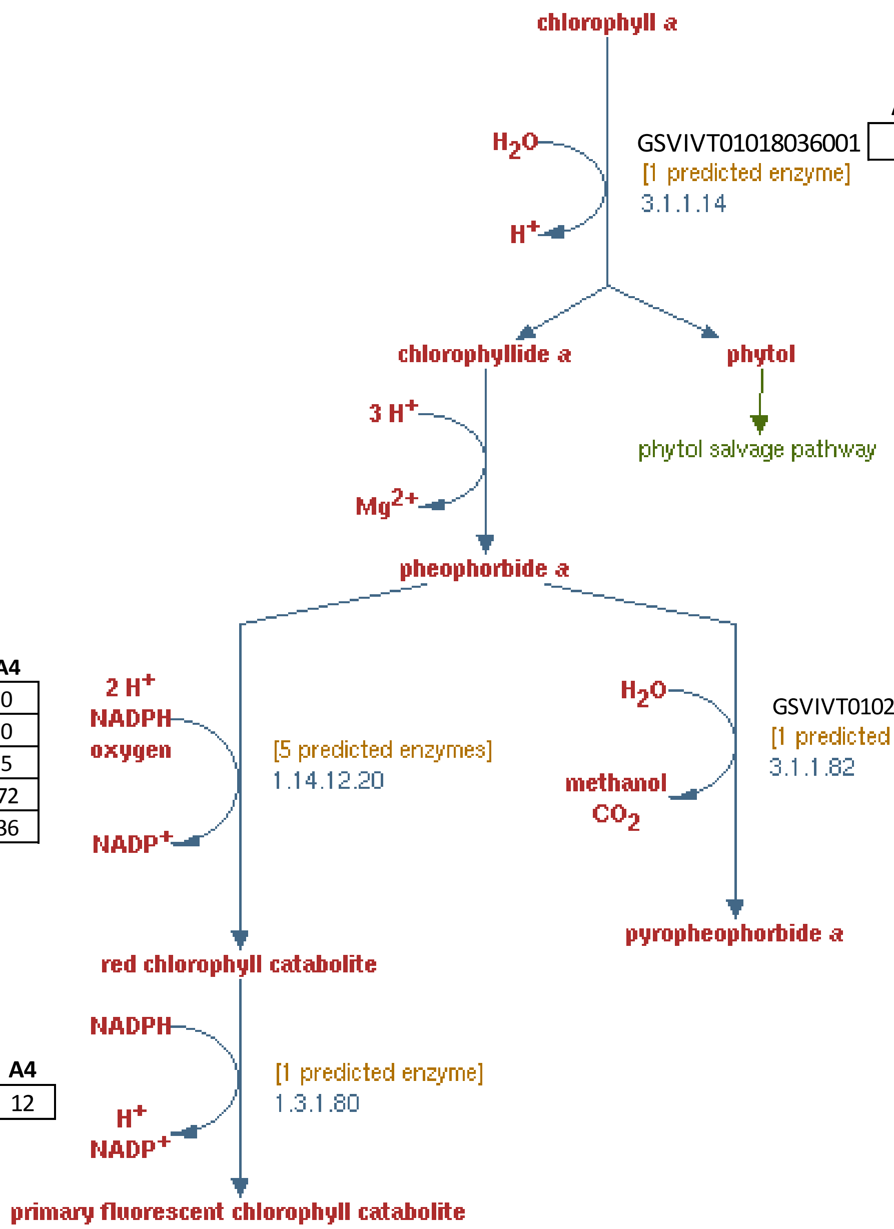

	A1	A2	A3	A4
GSVIVT01018036001	8	14	9	11

	A1	A2	A3	A4
GSVIVT01025441001	0	0	0	0
GSVIVT01025444001	1	0	0	0
GSVIVT01025446001	21	20	8	5
GSVIVT01031457001	81	93	91	72
GSVIVT01036007001	28	46	44	36

	A1	A2	A3	A4
GSVIVT01025948001	30	46	29	18

	A1	A2	A3	A4
GSVIVT01022135001	16	11	12	12

Pathway: chlorophyll a degradation II

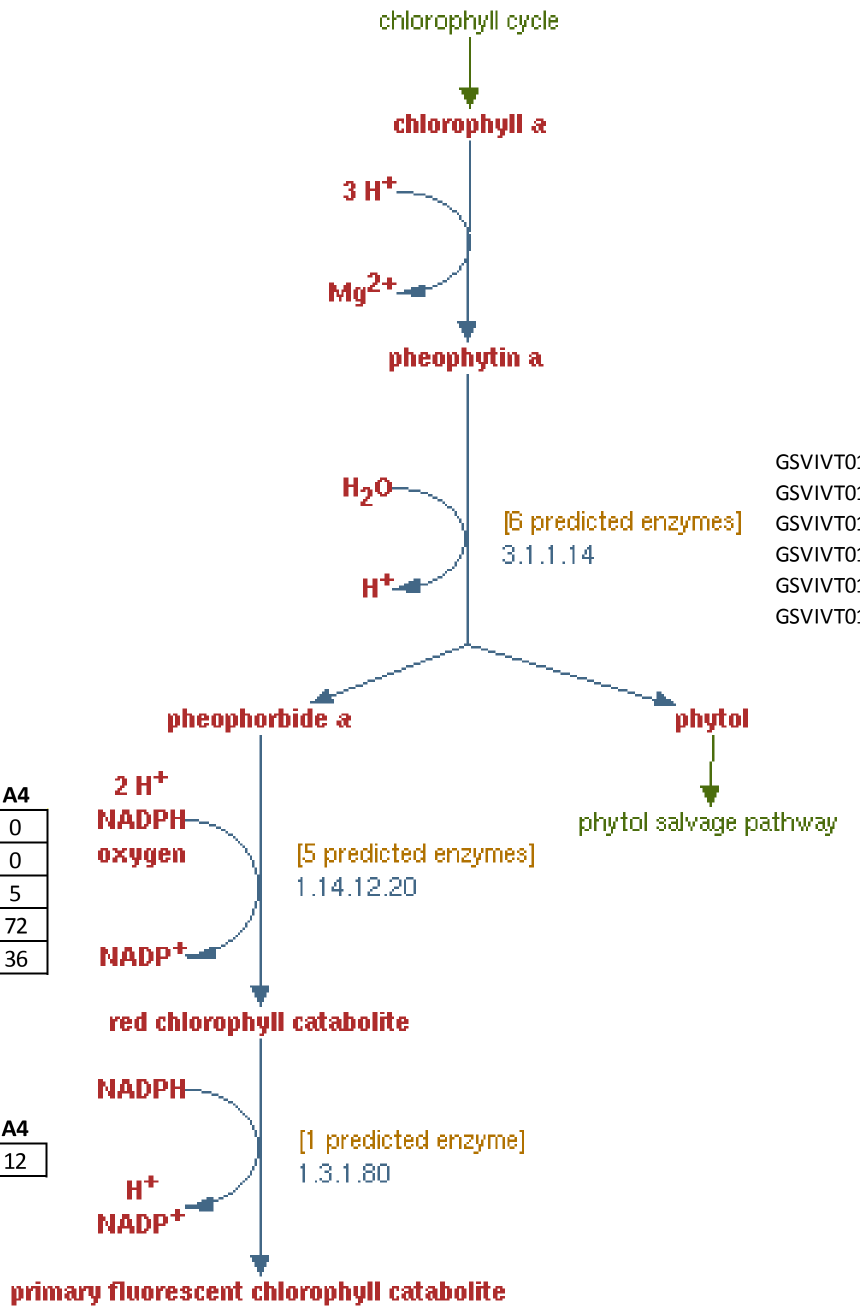

	A1	A2	A3	A4
GSVIVT01001445001	9	10	8	6
GSVIVT01018563001	9	8	8	7
GSVIVT01018934001	8	4	2	2
GSVIVT01025450001	5	5	4	4
GSVIVT01033023001	12	9	8	7
GSVIVT01037084001	8	9	4	3

	A1	A2	A3	A4
GSVIVT01025441001	0	0	0	0
GSVIVT01025444001	1	0	0	0
GSVIVT01025446001	21	20	8	5
GSVIVT01031457001	81	93	91	72
GSVIVT01036007001	28	46	44	36

	A1	A2	A3	A4
GSVIVT01022135001	16	11	12	12

Pathway: vitamin E biosynthesis (tocopherols)

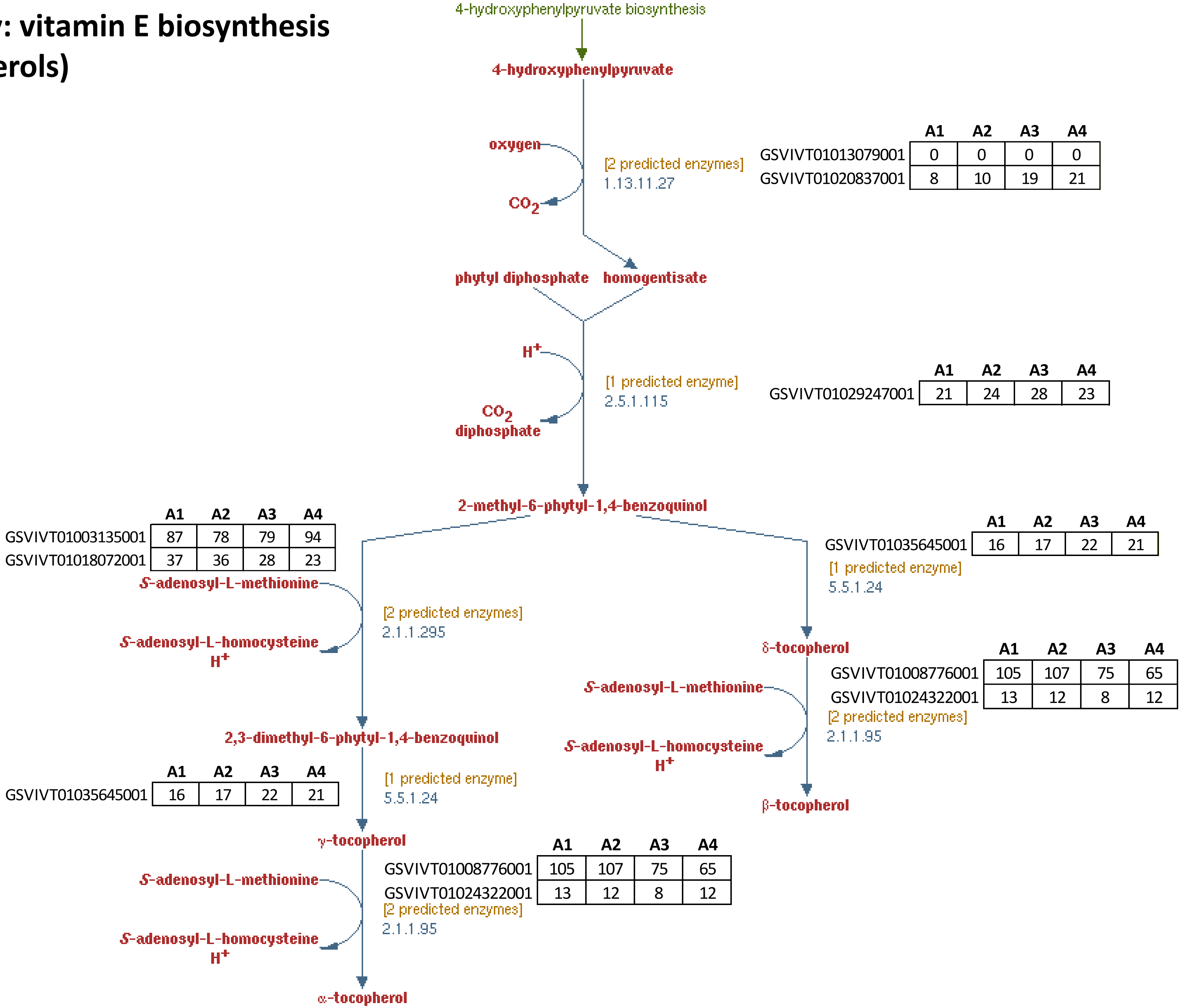

	A1	A2	A3	A4
GSVIVT01013079001	0	0	0	0
GSVIVT01020837001	8	10	19	21

	A1	A2	A3	A4
GSVIVT01029247001	21	24	28	23

	A1	A2	A3	A4
GSVIVT01003135001	87	78	79	94
GSVIVT01018072001	37	36	28	23

	A1	A2	A3	A4
GSVIVT01035645001	16	17	22	21

	A1	A2	A3	A4
GSVIVT01008776001	105	107	75	65
GSVIVT01024322001	13	12	8	12

	A1	A2	A3	A4
GSVIVT01035645001	16	17	22	21

	A1	A2	A3	A4
GSVIVT01008776001	105	107	75	65
GSVIVT01024322001	13	12	8	12

Pathway: fatty acid β-oxidation II (peroxisome)

	A1	A2	A3	A4
GSVIVT01000074001	19	18	17	10
GSVIVT01000075001	29	24	36	42
GSVIVT01017932001	23	36	48	44

	A1	A2	A3	A4
GSVIVT01015364001	32	33	43	32
GSVIVT01015482001	367	554	627	929
GSVIVT01017234001	8	6	8	10
GSVIVT01035128001	110	152	297	298
GSVIVT01035804001	881	1152	1303	1740
GSVIVT01035805001	108	197	282	329

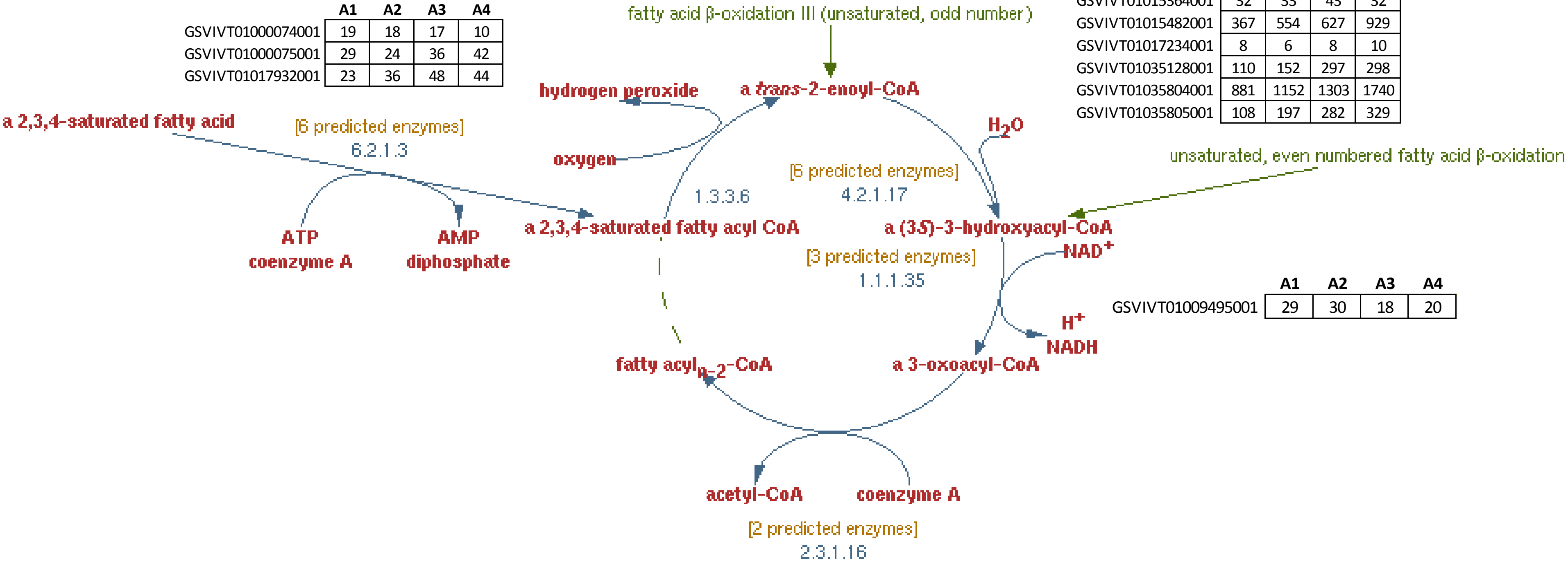

	A1	A2	A3	A4
GSVIVT01009495001	29	30	18	20

	A1	A2	A3	A4
GSVIVT01028557001	39	90	59	49
GSVIVT01029062001	106	172	252	257

sucrose biosynthesis II

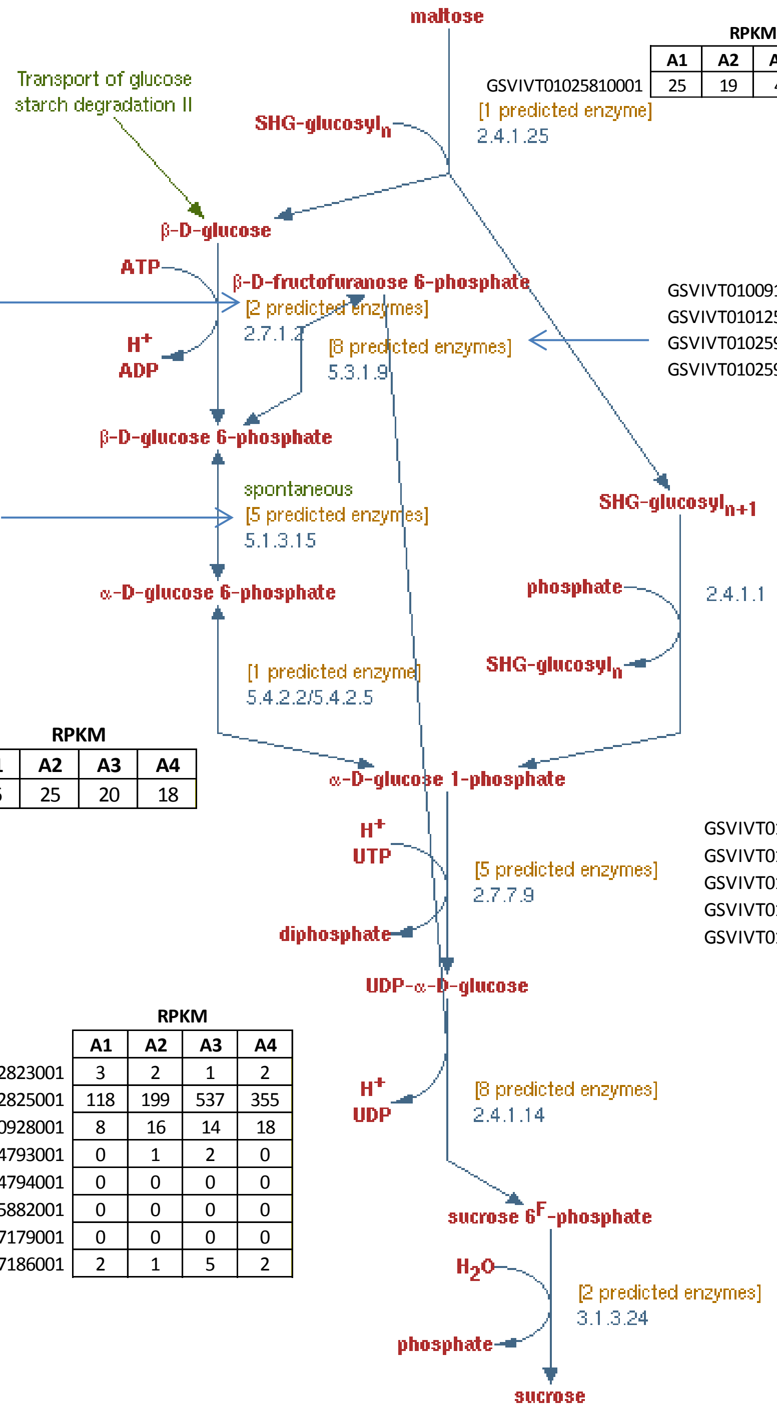

RPKM

	A1	A2	A3	A4
GSVIVT01025810001	25	19	4	4

RPKM

	A1	A2	A3	A4
GSVIVT01002667001	0	0	0	0
GSVIVT01006129001	0	0	0	0

RPKM

	A1	A2	A3	A4
GSVIVT01009147001	28	29	36	41
GSVIVT01012556001	0	0	0	0
GSVIVT01025959001	0	0	0	0
GSVIVT01025967001	0	0	0	0

RPKM

	A1	A2	A3	A4
GSVIVT01018938001	15	16	15	26
GSVIVT01019482001	13	5	3	3
GSVIVT01027042001	26	23	32	62
GSVIVT01029239001	177	139	136	139
GSVIVT01032393001	103	84	42	32

RPKM

	A1	A2	A3	A4
GSVIVT01018452001	26	25	20	18

RPKM

	A1	A2	A3	A4
GSVIVT01006086001	0	0	0	0
GSVIVT01008799001	124	157	266	215
GSVIVT01026561001	0	0	0	0
GSVIVT01026563001	437	562	994	1047
GSVIVT01032777001	9	8	5	6

RPKM

	A1	A2	A3	A4
GSVIVT01012823001	3	2	1	2
GSVIVT01012825001	118	199	537	355
GSVIVT01020928001	8	16	14	18
GSVIVT01034793001	0	1	2	0
GSVIVT01034794001	0	0	0	0
GSVIVT01035882001	0	0	0	0
GSVIVT01037179001	0	0	0	0
GSVIVT01037186001	2	1	5	2

RPKM

	A1	A2	A3	A4
GSVIVT01022530001	1	2	2	3
GSVIVT01029753001	11	6	10	12

Pathway: glyoxylate cycle

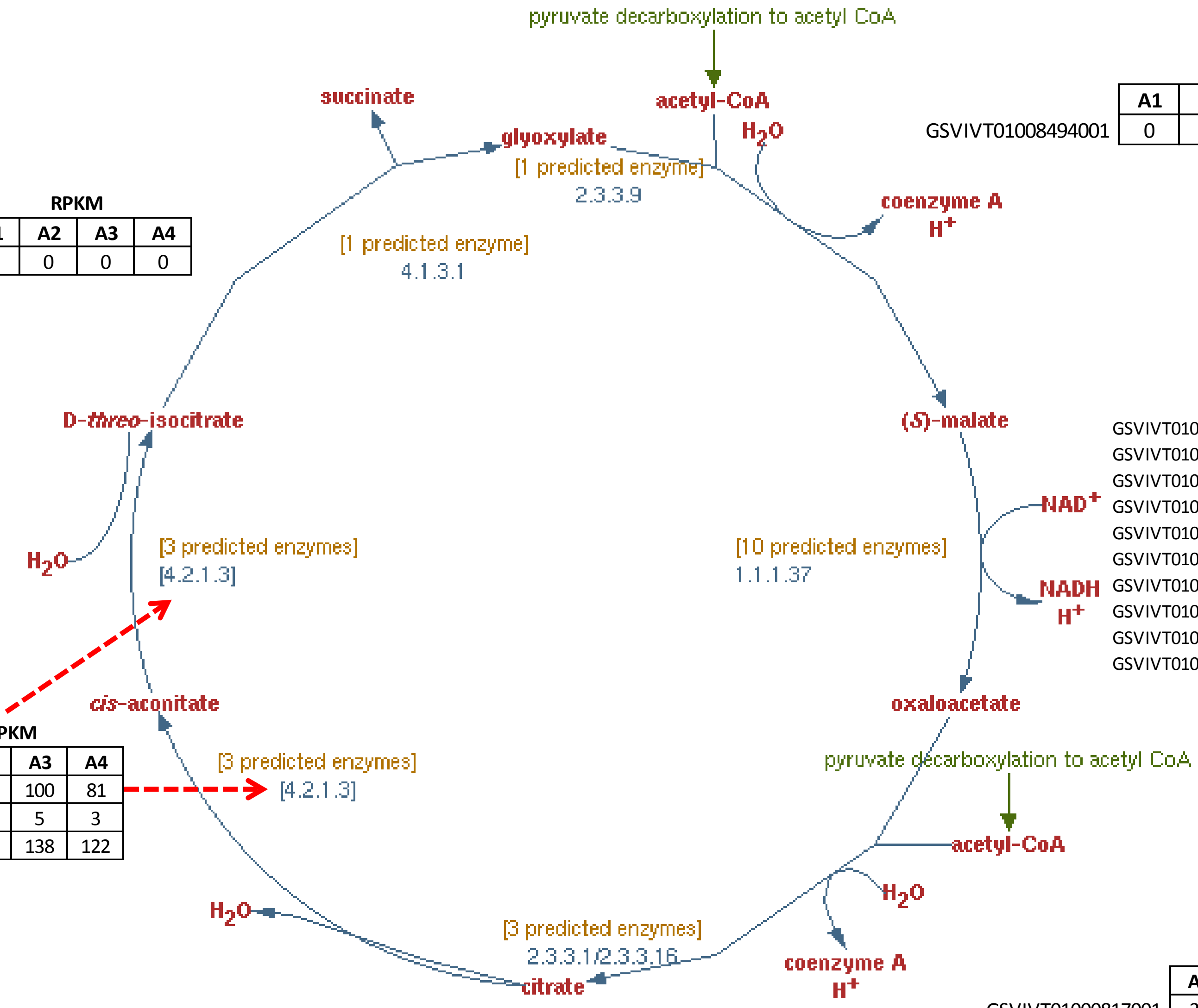

RPKM

	A1	A2	A3	A4
GSVIVT01008494001	0	0	0	0

RPKM

	A1	A2	A3	A4
GSVIVT01030608001	0	0	0	0

RPKM

	A1	A2	A3	A4
GSVIVT01006474001	53	39	60	62
GSVIVT01007997001	45	57	49	58
GSVIVT01010395001	2	3	2	3
GSVIVT01011422001	25	31	25	26
GSVIVT01014231001	31	24	45	54
GSVIVT01018190001	7	10	15	21
GSVIVT01021185001	160	186	270	344
GSVIVT01028330001	0	0	0	0
GSVIVT01028332001	0	0	0	0
GSVIVT01036965001	20	18	11	16

RPKM

	A1	A2	A3	A4
GSVIVT01005190001	122	108	100	81
GSVIVT01030588001	13	10	5	3
GSVIVT01037657001	90	94	138	122

RPKM

	A1	A2	A3	A4
GSVIVT01000817001	38	45	55	51
GSVIVT01001358001	71	77	98	98

pyruvate decarboxylation to acetyl CoA

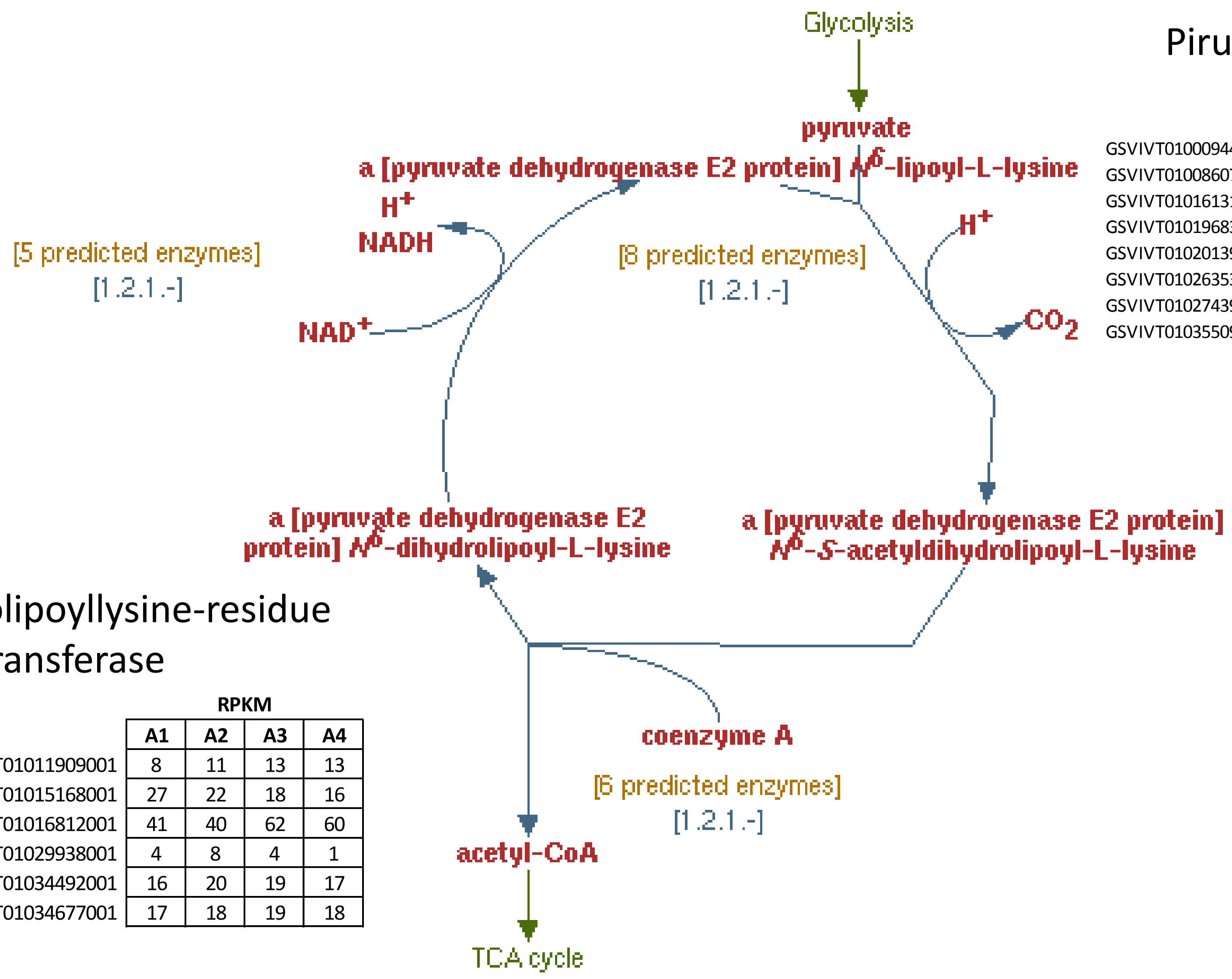

Piruvato DH

	RPKM			
	A1	A2	A3	A4
GSVIVT01000944001	66	73	150	150
GSVIVT01008607001	0	0	0	0
GSVIVT01016131001	0	0	0	0
GSVIVT01019683001	0	0	0	0
GSVIVT01020139001	4	5	9	11
GSVIVT01026353001	18	25	23	24
GSVIVT01027439001	57	81	80	76
GSVIVT01035509001	0	0	0	0

dihydrolipoyllysine-residue acetyltransferase

	RPKM			
	A1	A2	A3	A4
GSVIVT01011909001	8	11	13	13
GSVIVT01015168001	27	22	18	16
GSVIVT01016812001	41	40	62	60
GSVIVT01029938001	4	8	4	1
GSVIVT01034492001	16	20	19	17
GSVIVT01034677001	17	18	19	18

pyruvate decarboxylation to acetyl CoA

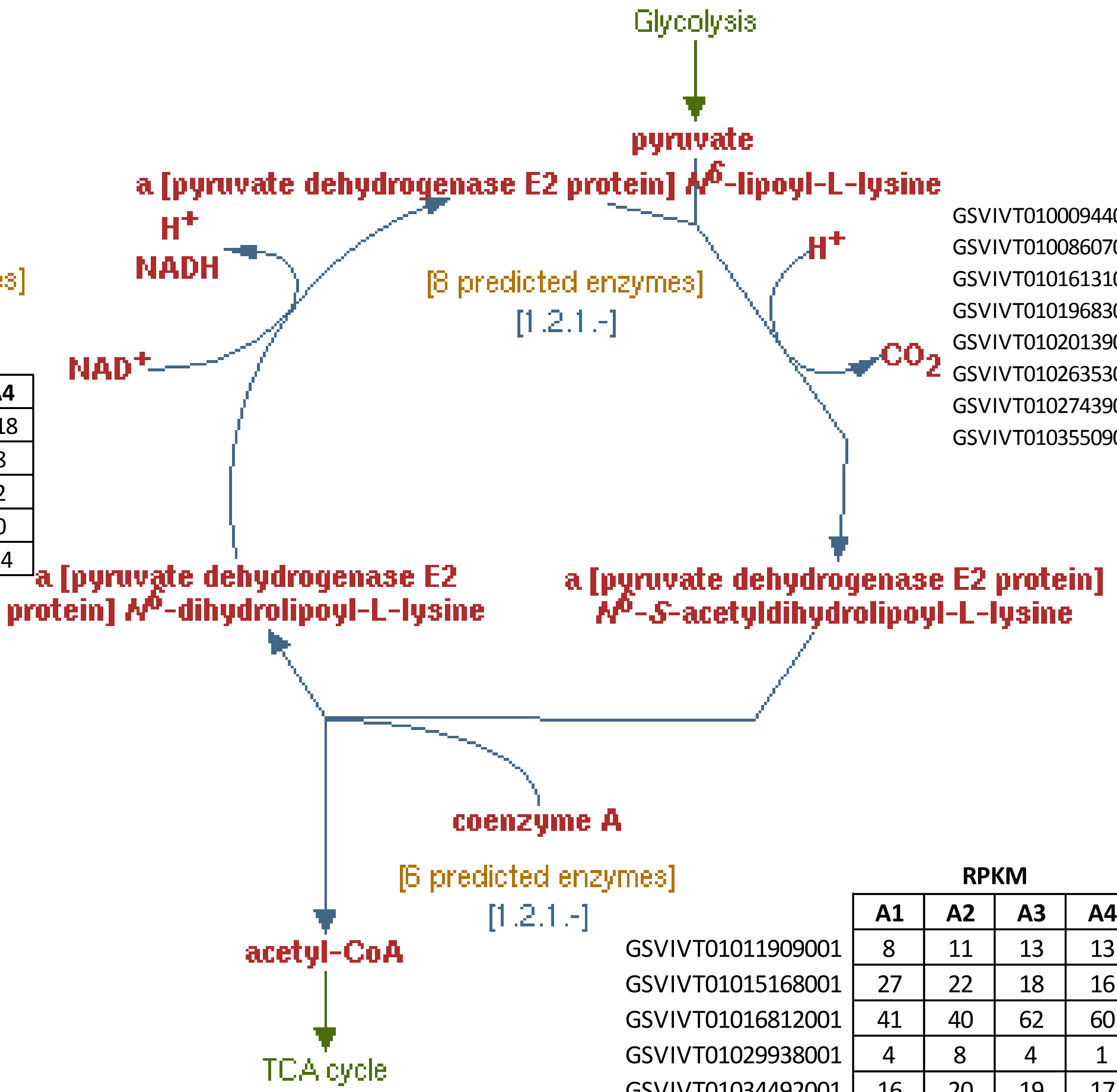

RPKM

	A1	A2	A3	A4
GSVIVT01017638001	112	163	192	218
GSVIVT01031216001	2	2	4	8
GSVIVT01031217001	2	3	2	2
GSVIVT01031225001	0	0	0	0
GSVIVT01035022001	46	48	29	24

RPKM

	A1	A2	A3	A4
GSVIVT01000944001	66	73	150	150
GSVIVT01008607001	0	0	0	0
GSVIVT01016131001	0	0	0	0
GSVIVT01019683001	0	0	0	0
GSVIVT01020139001	4	5	9	11
GSVIVT01026353001	18	25	23	24
GSVIVT01027439001	57	81	80	76
GSVIVT01035509001	0	0	0	0

RPKM

	A1	A2	A3	A4
GSVIVT01011909001	8	11	13	13
GSVIVT01015168001	27	22	18	16
GSVIVT01016812001	41	40	62	60
GSVIVT01029938001	4	8	4	1
GSVIVT01034492001	16	20	19	17
GSVIVT01034677001	17	18	19	18
